# Nonparametric Density Estimation of a Long-Term Trend from Repeated Semicontinuous Data

**DOI:** 10.1080/01621459.2025.2555054

**Published:** 2025-10-20

**Authors:** Félix Camirand Lemyre, Raymond J. Carroll, Aurore Delaigle

**Affiliations:** a Département de Mathématiques, Université de Sherbrooke, Sherbrooke, Canada; b Department of Statistics, Texas A&M University, College Station, TX; c School of Mathematics and Statistics, The University of Melbourne, Parkville, Australia

**Keywords:** Clumping at zero, Deconvolution, Episodically consumed food, Excess zeros, Measurement errors, Random effects, Repeated data, Two-part model, Zero-inflated

## Abstract

We consider nonparametric estimation of the density of the long-term trend of a semicontinuous variable observed repeatedly over time. These variables arise when measuring the intensity of an intermittent phenomenon, such as the intake of an episodically consumed nutrient or the concentration of an intermittent toxic substance: when the phenomenon is absent, the measurement is equal to zero; otherwise, it is positive. Semicontinuous data are usually represented by a two-part model describing the zeros and the nonzeros separately, often under parametric assumptions. Recently, Camirand Lemyre, Carroll, and Delaigle showed that it is possible to relax the distributional assumptions on the part that models the nonzeros, but like other existing work, they used a parametric model for the conditional probability H of observing a nonzero value. We develop a nonparametric estimator of H and of the density of the long-term trend. We illustrate our method on simulated examples and apply it to estimate the density of long-term fruit intake, using data from the Eating at America’s Table Study. Supplementary materials for this article are available online, including a standardized description of the materials available for reproducing the work.

## 1. Introduction

We consider nonparametric estimation of the density $f_{T}$ of the long-term trend T of the intensity of an intermittent phenomenon; this density is a useful tool for describing long-term behaviors of populations, such as the “usual” (i.e., long-term) intake of some nutrients often employed to monitor their eating behaviors. The long-term trend T is unobservable, and instead we collect several measurements of the short-term intensity for each individual. Specifically, for i=1,…,n and j=1,…,J, we observe the intensity $W_{ij}$ of the phenomenon (e.g., the nutrient intake) for the *i*th individual on the *j*th occasion. The variable $W_{ij}$ is semicontinuous because it is equal to zero if the phenomenon is absent on the *j*th occasion for the *i*th individual, and it is a positive continuous variable otherwise.

There is a literature modeling such semicontinuous data in the nutrition setting, resulting from the longstanding interest in the distribution of long-term food intake; see for example Tooze et al. (2006, 2010), Kipnis et al. (2009), Carroll (2014), and Kirkpatrick et al. (2017) for a review. There, the data are often obtained through national surveys (e.g., the Australian Health Survey or the National Health and Nutrition Examination Survey in the United States) using repeated 24-hour dietary recalls (24HRs). In a 24HR, individuals report their nutrient intakes of the last 24-hr period, and for i=1,…,n and j=1,…,J, $W_{ij}$ denotes the intake of a nutrient reported by the *i*th individual at the *j*th 24HR. For episodically consumed food such as fruits, $W_{ij}>0$ if the *i*th individual consumed the nutrient on day j and $W_{ij}=0$ otherwise.

Semicontinuous data also arise in a variety of other applications, for example when measuring the concentration of an intermittent toxic substance like pollution, drug or alcohol (Olsen and Schafer 2001), the intensity of/time spent at physical or relaxing activities not performed daily (Lopez et al. 2019), single cell gene expression rates (Finak et al. 2015), relative abundance of microbiomes (Chen and Li 2016) or health scores from self-reported health assessment questionnaires (Farewell et al. 2017); for general overviews, see Olsen and Schafer (2001), Min and Agresti (2002), and Liu et al. (2019). For other recent related works, see Klein, Kneib, and Lang (2015) and Zeng et al. (2023), who considered discrete data observed with excess zeros.

The excess zeros make it impossible to use the techniques commonly applied for analyzing continuous data measuring the intensity of non intermittent phenomena, such as the intake of daily consumed nutrients. In those simpler cases without excess zeros, the $W_{ij}$’s (or a transformed version of them) are often modeled by the classical measurement error model

(1.1) $$W_{ij}=X_{i}+U_{ij},$$

where $X_{i}∼f_{X}$ is the unobserved long-term trend and the $U_{ij}$’s are unobserved iid individual daily variations of zero mean, independent of $X_{i}$ and with density $f_{U}$. This model has generated a massive literature for estimating $f_{X}$; see Carroll et al. (2006) for a comprehensive review under parametric assumptions for $f_{X}$ and $f_{U}$. Carroll and Hall (1988) and Stefanski and Carroll (1990) proposed a more flexible nonparametric deconvolution kernel density estimator (DKDE)${\hat{f}}_{X,DKDE}$ of $f_{X}$ in the case where $f_{U}$ is known, which has been extended in various ways; see Delaigle (2014) and Delaigle and Van Keilegom (2021) for reviews and Delaigle and Van Keilegom (2021) for a review of the nonparametric literature where $f_{U}$ is estimated from data.

In the case with excess zeros that we study here, the zeros cannot be modeled by (1.1), so that the $W_{ij}$’s are usually represented by a two-part model: one part assumes that the positive $W_{ij}$’s (or a transformed version of them) follow the model at (1.1); a second part models the conditional probability *H* that $W_{ij}=0$, often using a logistic or probit model. Since the probability of observing a phenomenon is often related to the long-term trend of its intensity (e.g., high consumers are more likely to consume), it is common to connect the two parts through latent variables. Several versions of the two-part model have been suggested in the literature on semicontinuous data. They either use a complex model with two latent variables that requires parametric distributions for all variables of the model, which can be too restrictive in practice, or a model with one latent variable under which Camirand Lemyre, Carroll, and Delaigle (2022) developed a semiparametric estimator of the distribution of the long-term trend T using parametric assumptions only for H. In this work, we go one step further by proposing a flexible fully nonparametric estimator of $f_{T}$.

This article is organized as follows. We introduce the model and data in Section 2. We develop nonparametric estimators in Section 3, study asymptotic properties in Section 4, and discuss numerical implementation in Section 5. We investigate the practical performance of our procedure via simulation results in Section 6, and illustrate the method on data from the Eating at America’s Table Study in Section 7.

## 2. Model and Data

For i=1,…,n and j=1,…,J, we observe a short-term measurement $W_{ij}$ of the long-term trend $T_{i}$ of the intensity of an intermittent phenomenon for the *i*th individual, collected on the *j*th occasion which we refer to as day j. For the *i*th individual, if the phenomenon is not present on day j then $W_{ij}=0$; otherwise, $W_{ij}$ is a strictly positive continuous variable. As in Delaigle, Hall, and Meister (2008) and Camirand Lemyre, Carroll, and Delaigle (2022), the number *J* of replicates is such that 2≤J<∞.

As mentioned in Section 1, such data are usually represented by a two-part model that models separately the zeros and the positive $W_{ij}$’s. The latter, or often a version $h\left(W_{ij}\right)$ of them transformed by a known continuous, strictly increasing function h:(0,∞)→R, are commonly assumed to follow the model at (1.1). Accordingly, for all 1≤i≤n and 1≤j≤J such that $W_{ij}>0$, we assume that, conditional on $W_{ij}>0$,

(2.1) $${\tilde{W}}_{ij}=h\left(W_{ij}\right)=X_{i}+U_{ij},$$

where the $X_{i}$’s are iid with unknown continuous density $f_{X}$ and independent of the $U_{ij}$’s, and the $U_{ij}$’s are iid with a continuous density $f_{U}$ symmetric around 0. Throughout we use $f_{Z}$ to denote the density of any continuous random variable Z. For completeness, for i, j such that $W_{ij}=0$, we can also define $U_{ij}∼f_{U}$ independent of $X_{i}$ and let ${\tilde{W}}_{ij}=X_{i}+U_{ij}$, but ${\tilde{W}}_{ij}$ and $U_{ij}$ are irrelevant in this case since they do not correspond to any observed data; in particular, they are not such that ${\tilde{W}}_{ij}=h\left(W_{ij}\right)$. In applications where the data are not transformed, h in (2.1) is the identity function. When a transformation h is used, it is usually a log or (less often) a power function because the distribution of the raw positive $W_{ij}$’s is typically right-skewed. There also exist methods for choosing h more adaptively; see for example Eckert, Carroll, and Wang (1997). In real applications in statistics, it is common to apply methods and models to transformed data rather than raw data, even though data transformations are often not tracked explicitly in methodological developments. In the case of semicontinuous observations, we carefully keep track of the transformation h because it is only applied to the positive $W_{ij}$’s and impacts the distribution of the long-term trend in a less trivial way; see Section 3.

The $U_{ij}$’s represent zero mean daily fluctuations around $X_{i}$. They play the role of the errors in (1.1) and are assumed to be independent of the $I\left\{W_{ij}>0\right\}$’s. As usual in the literature, we assume that the repeated $W_{ij}$’s for the *i*th individual are taken far apart in time so that conditionally on $X_{i}$, they are independent. As common in the measurement error literature, we assume that

(2.2) $$\phi_{U}(t)\neq 0\;\text{for all}\;t,$$

where throughout we use $\phi_{f}$ to denote the Fourier transform of a function f or the characteristic function of a variable f. This is satisfied by the most commonly employed error distributions such as normal or Laplace. In some cases, $f_{U}$ may be known; otherwise it can be estimated from the replicates using Delaigle, Hall, and Meister’s (2008) approach extended to the excess zeros context, as in Camirand Lemyre, Carroll, and Delaigle (2022).

The second part of the model deals with the zeros. Following the one latent variable version of the model (e.g., Wang, Feng, and Song 2020; Camirand Lemyre, Carroll, and Delaigle 2022), we assume that, for 1≤i≤n and 1≤j≤J, there is a continuous function H:R→[0,1] such that

(2.3) $$P\left(W_{ij}>0|X_{i}=x\right)=P\left(I\left\{W_{ij}>0\right\}=1|X_{i}=x\right)=H(x).$$

The literature invariably assumes a monotone parametric form for H, often using a probit or logistic model. However, in real applications, it can be challenging to determine a suitable parametric form for H because it is the expectation of a binary variable conditional on an unobservable variable. To avoid misspecification bias, we make no parametric assumption on H and only assume that it is strictly increasing. This assumption is not necessary for constructing a nonparametric estimator of H, and in Section 3.2 we will derive an estimator $\hat{H}$ that is consistent without it. However, the monotonicity of H will enable us to construct a refined version of $\hat{H}$ that has better practical performance and whose invertibility is useful for estimating the density of long term intake. This assumption is quite natural; for example, using (2.4), it implies that the stronger the trend of a phenomenon, the higher the probability of observing it on a particular day. Nevertheless, and although it is milder than assuming that H follows a logistic or probit model, it may be still too restrictive in some applications.

The repeated observations $W_{ij}$ are short-term measurements of the long-term trend $T_{i}$ of the *i*th individual. The latter is obtained by averaging the $W_{ij}$’s over the random daily fluctuations of the *i*th individual, that is (see e.g., Kipnis et al. 2009)

(2.4) $$T_{i}=E\left(W_{ij}|X_{i}\right)=R\left(X_{i}\right),$$

where, denoting the inverse of a function f by $f^{\left(‐1\right)}$, the convolution product of two functions $f_{1}$ and $f_{2}$ by $f_{1}*f_{2}(x)=\int \;f_{1}(x‐y)f_{2}(y)dy$, and recalling that h is strictly increasing,

(2.5) $$R\left(x\right)=H\left(x\right)\cdot \left(h^{\left(‐1\right)}*f_{U}\right)\left(x\right).$$

If R is strictly increasing (which is satisfied under our assumptions that H and h are strictly increasing), it is invertible and under the mild additional assumption that R has a strictly positive continuous derivative, the density $f_{T}$ of $T_{i}$ takes a simple expression given at (3.1). Technically, it is not essential to assume that H is strictly increasing since it is possible for R to be strictly increasing under milder conditions on H; however, we will see in Section 3 that this assumption helps improve the performance of a nonparametric estimator. The monotonicity constraint on h is important: without it, R would take a more complex form that would be difficult to handle. Likewise, if R is not strictly monotone, $f_{T}$ would take a form that is more complex than (3.1), and which would be much more difficult to estimate: for example, it would require splitting the real line into consecutive intervals where R is strictly monotone. These complexities seem largely unnecessary in practice since it is often reasonable to assume that H and h, and thus R, are strictly increasing.

## 3. Methodology

Our goal is to estimate nonparametrically the density

(3.1) $$f_{T}(t)=f_{R(X)}(t)={\left\{R^{\left(‐1\right)}(t)\right\}}^{'}f_{X}\left\{R^{\left(‐1\right)}(t)\right\}$$

of $T_{i}=E\left(W_{ij}|X_{i}\right)$ from a sample of observations $W_{ij}$, i=1,…,n, j=1,…,J, that satisfy the model assumptions from Section 2. The task is challenging because it requires to estimate $f_{X},$ H, $R^{\left(‐1\right)}$ and ${\left\{R^{\left(‐1\right)}\right\}}^{'}$ nonparametrically. Camirand Lemyre, Carroll, and Delaigle (2022) studied the related problem of estimating the distribution $F_{T}$ of $T_{i}$, which is much easier because it only requires to estimate H and $f_{X}$. Moreover, they were only able to construct a semiparametric estimator of $F_{T}$ under parametric assumptions for H.

To simplify exposition, we introduce our method assuming that the density $f_{U}$ is known. In practice it is often estimated from the replicates, either parametrically or nonparametrically, as in Camirand Lemyre, Carroll, and Delaigle (2022). Then the only change to the procedure we introduce is to replace $f_{U}$ and its characteristic function $\phi_{U}$ by their estimators. See Appendix A.1 for details and Section 6 for numerical illustrations.

### 3.1. Estimating $f_{X}$

To see how to estimate $f_{X}$, we start by summarizing the steps for constructing Stefanski and Carroll’s (1990) DKDE of $f_{X}$ in the simpler case where the data come from the classical model at (1.1). There, the densities of $W_{ij}$, $X_{i}$, and $U_{ij}$ satisfy $f_{W}=f_{X}*f_{U}$, or, equivalently, $\phi_{W}=\phi_{X}\phi_{U}$, where, throughout, for two functions $f_{1}$, $f_{2}$ with matching domains, we use $f_{1}f_{2}$ or ($f_{1}f_{2}$) to represent the function equal to the product of $f_{1}$ and $f_{2}$. Now, by the Fourier inversion theorem (Theorem C.1 in Appendix C), under (2.2), if $f_{X}$ is continuous and has a piecewise continuous derivative, then for all x∈R,

(3.2) $$\begin{array}{rr} f_{X}(x) & \;=(2\pi {)}^{‐1}\int \;e^{‐itx}\phi_{X}(t)dt\\ & \;=(2\pi {)}^{‐1}\int \;e^{‐itx}\phi_{W}(t)/\phi_{U}(t)dt, \end{array}$$

where throughout, integrals with unspecified bounds denote Lebesgue integrals on R, except for inverse Fourier transform integrals, where for any Lebesgue integrable and continuous function f with a piecewise continuous derivative, we define $\int \;e^{‐itx}\phi_{f}(t)dt$ as the improper Riemann integral ${lim}_{T\to \infty }\;\int_{‐T}^{T}\;e^{‐itx}\phi_{f}(t)dt$ (see Appendix C).

Stefanski and Carroll (1990) estimate $\phi_{X}$ by ${\hat{\phi }}_{X}={\hat{\phi }}_{W}/\phi_{U}$, with ${\hat{\phi }}_{W}$ the empirical characteristic function of the $W_{ij}$’s, and then estimate $f_{X}(x)$ nonparametrically by

(3.3) $${\hat{f}}_{X,DKDE}(x)=\frac{1}{2\pi }\int_{‐\infty }^{\infty }\;e^{‐itx}\omega (t){\hat{\phi }}_{W}(t)/\phi_{U}(t)dt,$$

where ω is a weight function that dampens down the inaccurate tails of ${\hat{\phi }}_{W}$, to ensure that the integral at (3.3) exists. They take $\omega (t)=\phi_{K}(ht)$, with K a smooth even kernel function which integrates to 1, and h>0 a smoothing parameter called bandwidth, that dictates the amount of dampening applied to the tails.

Things are more complex in our case with excess zeros because the ${\tilde{W}}_{ij}$’s at (2.1) are only observed if $W_{ij}>0$. While (2.1) is similar to (1.1), $f_{\tilde{W}|W>0}$, $f_{X}$ and $f_{U}$ do not satisfy $f_{\tilde{W}|W>0}=f_{X}*f_{U}$. Instead, using (2.1), (2.3), the fact that H is strictly increasing, and the independence assumptions from Section 2, we have, as in Camirand Lemyre, Carroll, and Delaigle (2022),

(3.4) $$P\left(W>0\right)f_{\tilde{W}|W>0}\left(w\right)=\left(Hf_{X}\right)*f_{U}\left(w\right),$$

or equivalently, $\phi_{{\tilde{W}}_{+}}=\phi_{Hf_{X}}\phi_{U}$, with $\phi_{{\tilde{W}}_{+}}$ the Fourier transform of $P(W>0)f_{\tilde{W}|W>0}$. Using (2.2) and Theorem C.1 in Appendix C, if $Hf_{X}$ is continuous and has a piecewise continuous derivative, then for all x∈R,

(3.5) $$g(x)\equiv \left(Hf_{X}\right)(x)=(2\pi {)}^{‐1}\int \;e^{‐itx}\phi_{{\tilde{W}}_{+}}(t)/\phi_{U}(t)dt.$$

This motivated them to propose the following nonparametric kernel estimator of g(x):

(3.6) $$\hat{g}(x)\equiv \hat{\left(Hf_{X}\right)}(x)=\frac{1}{2\pi }\int_{‐\infty }^{\infty }\;e^{‐itx}{\hat{\phi }}_{{\tilde{W}}_{+}}(t)\phi_{K}(ht)/\phi_{U}(t)dt,$$

with K and h>0 as in (3.3), and where ${\hat{\phi }}_{{\tilde{W}}_{+}}(t)$ is an estimator of $\phi_{{\tilde{W}}_{+}}(t)$ defined by

(3.7) $${\hat{\phi }}_{{\tilde{W}}_{+}}\left(t\right)=\frac{1}{nJ}\sum_{k=1}^{n}\;\sum_{j=1}^{J}\;e^{it}{\tilde{W}}_{kj}I\left(W_{kj}>0\right).$$

Since $g=Hf_{X}$, we can deduce an estimator of $f_{X}$ by estimating the nuisance function $H(x)=P\left(W_{ij}>0|X_{i}=x\right)$ caused by the excess zeros. This is not easy: as the $X_{i}$’s are latent, we do not have access to the $\left(X_{i},I\left(W_{ij}>0\right)\right)$’s from which to compute a standard nonparametric regression estimator of $H(x)=E\left\{I\left(W_{ij}>0\right)|X_{i}=x\right\}$. Also, unlike classical measurement error problems without excess zeros, we cannot use the observed version $\left(W_{ij},I\left(W_{ij}>0\right)\right)$ of the $\left(X_{i},I\left(W_{ij}>0\right)\right)$’s to construct an alternative estimator of H: using (3.4), these data can be used to estimate $Hf_{X}$, but to deduce an estimator of H we would need to estimate $f_{X}$, whereas it is to estimate $f_{X}$ that we need to estimate H. To solve this circular argument, Camirand Lemyre, Carroll, and Delaigle (2022) imposed a parametric model for H, which they estimated using a method of moments construction. Using $\hat{g}$ at (3.6), they deduced a semiparametric estimator ${\hat{f}}_{X,SP}$ of $f_{X}$.

Next we show that, while the estimation difficulties for estimating $f_{X}$ nonparametrically in our model stem from the excess zeros, when exploited properly through repeated use of their relationship with H in (3.4), they can actually be leveraged to eliminate H from (3.6). Indeed, using a second replicate $W_{ik}$ for which $W_{ik}>0$, we can induce a squared version of H through the conditional joint density

(3.8) $$\begin{array}{rr} & f_{{\tilde{W}}_{ij},{\tilde{W}}_{ik}|W_{ij}>0,W_{ik}>0}(w,v)\\ & \;=\int \;P\left(W_{ij}>0,W_{ik}>0|X_{i}=x\right)f_{U}(w‐x)f_{U}(v‐x)f_{X}(x)\\ & \;\times dx/P\left(W_{ij}>0,W_{ik}>0\right)\\ & \;=\int \;H^{2}(x)f_{U}(w‐x)f_{U}(v‐x)f_{X}(x)dx/P\left(W_{ij}>0,W_{ik}>0\right). \end{array}$$

To compute (3.8), we used the model assumptions from Section 2; in particular, we exploited the fact that, since the $W_{ij}$’s are independent conditionally on $X_{i}$, then for 1≤i≤n and j≠k, we have, using (2.3),

(3.9) $$P\left(W_{ij}>0,W_{ik}>0|X_{i}=x\right)=H^{2}(x),$$

which also implies that $P\left(W_{ij}>0,W_{ik}>0\right)>0$ since H is strictly increasing.

Taking v=w in (3.8), we deduce that

(3.10) $$\begin{array}{r} P\left(W_{ij}>0,W_{ik}>0\right)f_{{\tilde{W}}_{ij},{\tilde{W}}_{ik}|W_{ij}>0,W_{ik}>0}(w,w)\\ =\left(H^{2}f_{X}\right)*f_{U}^{2}(w). \end{array}$$

Proceeding as in (3.6), we can derive a nonparametric estimator of $H^{2}f_{X}$ from (3.10), plug it with $\hat{g}$ into the expression $f_{X}=g^{2}/\left(H^{2}f_{X}\right)$, and deduce a nonparametric estimator of $f_{X}$. However, we can bypass the estimation of the bivariate density $f_{{\tilde{W}}_{ij},{\tilde{W}}_{ik}|W_{ij}>0,W_{ik}>0}$ since, by integrating (3.8) with respect to v, we find

(3.11) $$P\left(W_{ij}>0,W_{ik}>0\right)f_{{\tilde{W}}_{ij}|W_{ij}>0,W_{ik}>0}(w)=\left(H^{2}f_{X}\right)*f_{U}(w).$$

Equivalently, using (2.2), $\phi_{H^{2}f_{X}}=\phi_{{\tilde{W}}_{++}}/\phi_{U}$, where $\phi_{{\tilde{W}}_{++}}$ is the Fourier transform of $P\left(W_{ij}>0,W_{ik}>0\right)f_{{\tilde{W}}_{ij}|W_{ij}>0,W_{ik}>0}$. Using Theorem C.1 again, if $H^{2}f_{X}$ is continuous and has a piecewise continuous derivative, then for all x∈R we have

(3.12) $$\left(H^{2}f_{X}\right)(x)=\frac{1}{2\pi }\int \;e^{‐itx}\phi_{{\tilde{W}}_{++}}(t)/\phi_{U}(t)dt.$$

Combining this with (3.5), Proposition C.2 in Appendix C establishes the identifiability of $f_{X}$ on R and of H(x) for all x∈R such that $f_{X}(x)>0$, from the joint distribution of two replicates $W_{i1}$ and $W_{i2}$.

Now we can estimate $\phi_{{\tilde{W}}_{++}}(t)$ by

$${\hat{\phi }}_{{\tilde{W}}_{++}}(t)=\frac{1}{nJ(J‐1)}\sum_{k=1}^{n}\;\sum_{j\neq j^{'}}\;e^{it{\tilde{W}}_{kj}}I\left(W_{kj}>0\right)I\left(W_{kj^{'}}>0\right).$$

Plugging this into (3.12) and regularizing the integral with a kernel weight $\phi_{K}(ht)$ as in (3.3), we get the following deconvolution kernel estimator of $\left(H^{2}f_{X}\right)(x)$:

(3.13) $$\hat{\left(H^{2}f_{X}\right)}(x)=\frac{1}{2\pi }\int \;e^{‐itx}{\hat{\phi }}_{{\tilde{W}}_{++}}(t)\phi_{K}(ht)/\phi_{U}(t)dt.$$

Recalling $\hat{g}$ at (3.6), we deduce a nonparametric estimator of $f_{X}(x)$ by taking

(3.14) $${\hat{f}}_{X}(x)={\hat{g}}^{2}(x)/\hat{\left(H^{2}f_{X}\right)}(x).$$

Since $f_{X}$ is a density, in practice we take $max\left\{{\hat{f}}_{X}(x),0\right\}$.

#### Remark 3.1.

Like usual deconvolution kernel estimators, since $f_{U}$ is even (as assumed below (2.1)), then as long as K is also even, which we assume in Assumption (A2), our deconvolution estimators are real functions; that is, their imaginary part vanishes. Indeed, under these assumptions, since the sine function is odd then $\phi_{K}$ and $\phi_{U}$ are real, even functions. Since $e^{‐it\left(x‐{\tilde{W}}_{kj}\right)}=cos\left\{t\left(x‐{\tilde{W}}_{kj}\right)\right\}‐i\;\text{sin}\left\{t\left(x‐{\tilde{W}}_{kj}\right)\right\}$, and exploiting again the parity of the sine function, it is straightforward to deduce that the imaginary part of the integrals at (3.6) and(3.13) vanishes.

### 3.2. Estimating H

A first nonparametric estimator of H(x) can be obtained by taking

(3.15) $$\hat{H}(x)=\hat{\left(H^{2}f_{X}\right)}(x)/\hat{g}(x).$$

$\hat{H}$ is consistent, but in small to moderate samples, it performs poorly in the tails of $f_{X}$ due to the data sparsity in the tails of $f_{\tilde{W}|W>0}$, and is rarely strictly increasing, whereas H is. We can strictly increasing estimator. This not only makes the estimator address both issues simultaneously by transforming $\hat{H}$ into a strictly increasing estimator. This not only makes the estimator satisfy the same shape constraint as H, but also makes it less variable, which improves its practical performance near the tails.

We could modify $\hat{H}$ into a strictly increasing estimator by using a tilting method of the type in Carroll, Delaigle, and Hall (2011). Adapted to our context, it would consist in incorporating individual weights $p_{kj}$ in the sum of the estimator ${\hat{\phi }}_{{\tilde{W}}_{++}}$ used in (3.13), in such a way that the weighted estimator is strictly increasing. However, that estimator is not naturally strictly increasing and we found that we could get better practical results by using an improved version of Dette, Neumeyer, and Pilz’s (2006) monotonization procedure. Adapted to the estimation of H on an interval $\left[a_{H},b_{H}\right]$, their method uses the fact that the density $f_{V}$ of V=H(Υ), with $\Upsilon ∼U\left[a_{H},b_{H}\right]$, satisfies

(3.16) $$\begin{array}{r} H^{\left(‐1\right)}(t)=a_{H}+\left(b_{H}‐a_{H}\right)\int_{H\left(a_{H}\right)}^{t}\;\;f_{V}(z)dz\\ \;\text{for all}\;t\in \left[H\left(a_{H}\right),H\left(b_{H}\right)\right]. \end{array}$$

For $t\in \left[m_{\hat{H}},M_{\hat{H}}\right]\equiv \left[{min}_{x\in \left[a_{H},b_{H}\right]}\;\hat{H}(x),{max}_{x\in \left[a_{H},b_{H}\right]}\;\hat{H}(x)\right]$, that procedure estimates $H^{\left(‐1\right)}(t)$ by $\hat{H^{\left(‐1\right)}}(t)$ obtained by estimating, in (3.16), $H\left(a_{H}\right)$ by $m_{\hat{H}}$, $H\left(b_{H}\right)$ by $M_{\hat{H}}$, and $f_{V}$ by a standard kernel density estimator (KDE) $\hat{f_{V}}.^{KDE}$. This KDE is computed from the sample $V_{1}=\hat{H}\left(v_{1}\right),\ldots ,V_{n^{+}}=\hat{H}\left(v_{n^{+}}\right)$, where the $v_{i}$’s are equispaced on $\left[a_{H},b_{H}\right]$ and $n^{+}=\sum_{i=1}^{n}\;\sum_{j=1}^{J}\;I\left(W_{ij}>0\right)$ is the number of ${\tilde{W}}_{ij}$’s used to compute $\hat{H}$.

Following Dette, Neumeyer, and Pilz (2006), we can obtain a strictly increasing estimator of H by numerically inverting $\hat{H^{\left(‐1\right)}}$. However, that estimator is significantly biased near $a_{H}$ and $b_{H}$ because $f_{V}$ is not continuous at the endpoints $H\left(a_{H}\right)$ and $H\left(b_{H}\right)$ of its support, and standard KDEs perform poorly in a relatively large neighborhood of the endpoints in that case. We can reduce this bias significantly by estimating $f_{V}$ using an approach specifically designed for compactly supported densities. We use Geenens (2014) probit local likelihood estimator ${\hat{f}}_{V}^{LL}$ with standard normal kernel, which seems to outperform otherexisting boundary-adjusted kernel estimators in practice; see Appendix A.2.3 for how we adapted that estimator to our problem. Because of numerical issues discussed in Appendix A.2.3, a drawback of ${\hat{f}}_{V}^{LL}$ is that is not defined at the endpoints of the support themselves, and can be unreliable in a small neighborhood of those points, whereas to estimate (3.16) we need an estimator of $f_{V}$ on the whole $\left[m_{\hat{H}},M_{\hat{H}}\right]$. This motivates us to use ${\hat{f}}_{V}^{LL}$ only on $\left[m_{\hat{H}}+\Delta ,M_{\hat{H}}‐\Delta \right]$, where $\Delta =\lambda \left(M_{\hat{H}}‐m_{\hat{H}}\right)$ with λ∈(0,1/2) a small number (see Sections 4 and 5.3). Then we extend ${\hat{f}}_{V}^{LL}$ to [$m_{\hat{H}},M_{\hat{H}}$] by extrapolation by taking, for $z\in \left[m_{\hat{H}},M_{\hat{H}}\right]$,

(3.17) $${\overset{ˇ}{f}}_{V}^{LL}(z)=\left\{\begin{array}{ll} c_{1}(z) & \;\text{if}\;m_{\hat{H}}\leq z\leq m_{\hat{H}}+\Delta \\ {\hat{f}}_{V}^{LL}(z) & \;\text{if}\;m_{\hat{H}}+\Delta \leq z\leq M_{\hat{H}}‐\Delta \\ c_{2}(z) & \;\text{if}\;M_{\hat{H}}‐\Delta \leq z\leq M_{\hat{H}}, \end{array}\right.$$

where $c_{1}$ and $c_{2}$ are bounded positive constant or linear extrapolation functions. Finally, for $t\in \left[m_{\hat{H}},M_{\hat{H}}\right]$, we estimate $H^{\left(‐1\right)}(t)$ by

(3.18) $$\hat{H^{\left(‐1\right)}}\left(t\right)=a_{H}+\left(b_{H}‐a_{H}\right)\int_{m_{\hat{H}}}^{t}\;{\tilde{f}}_{V}^{LL}\left(z\right)dz,$$

where ${\tilde{f}}_{V}^{LL}$ is a rescaled version of ${\overset{ˇ}{f}}_{V}^{LL}$ so that $\int_{m_{\hat{H}}}^{M_{\hat{H}}}\;{\tilde{f}}_{V}^{LL}=1$. Our final estimator of H is the strictly increasing version of ${\hat{H}}_{M}$ of $\hat{H}$ on $\left[a_{H},b_{H}\right]$ obtained by numerically inverting $\hat{H^{\left(‐1\right)}}$. More details of practical implementation will be provided in Section 5.

### 3.3. Estimating $f_{T}$

To estimate $f_{T}$ at (3.1), it remains to estimate $R^{\left(‐1\right)}$ and ${\left\{R^{\left(‐1\right)}\right\}}^{'}$. Estimating $R^{\left(‐1\right)}$ is simple. Recalling (2.5), we start by estimating R, on the interval [$a_{H},b_{H}$] where we computed ${\hat{H}}_{M}$ by

(3.19) $${\hat{R}}_{M}={\hat{H}}_{M}\cdot \left(h^{\left(‐1\right)}*f_{U}\right).$$

Then, we obtain an estimator ${\hat{R}}_{M}^{\left(‐1\right)}$ of $R^{\left(‐1\right)}$ on the interval $\left[{\hat{R}}_{M}\left(a_{H}\right),{\hat{R}}_{M}\left(b_{H}\right)\right]$ by numerically inverting ${\hat{R}}_{M}$.

Estimating ${\left\{R^{\left(‐1\right)}\right\}}^{'}$ on $\left[{\hat{R}}_{M}\left(a_{H}\right),{\hat{R}}_{M}\left(b_{H}\right)\right]$ is less simple. One approach is to use arguments similar to those used tomonotonize $\hat{H}$ in Section 3.2, as follows. Since R is strictly increasing on $\left[a_{H},b_{H}\right]$, then similar to (3.16), for any $t\in \left[R\left(a_{H}\right),R\left(b_{H}\right)\right]$ we have $R^{\left(‐1\right)}(t)=a_{H}+\left(b_{H}‐a_{H}\right)\int_{R\left(a_{H}\right)}^{t}\;f_{V}$, where $f_{V}$ is the density of V=R(Υ), with $\Upsilon ∼U\left[a_{H},b_{H}\right]$. Taking the derivative with respect to t, we get ${\left\{R^{\left(‐1\right)}\right\}}^{'}(t)=\left(b_{H}‐a_{H}\right)f_{V}(t)$. For $t\in \left[{\hat{R}}_{M}\left(a_{H}\right),{\hat{R}}_{M}\left(b_{H}\right)\right]$, we deduce a smooth estimator of ${\left\{R^{\left(‐1\right)}\right\}}^{'}(t)$ by taking

(3.20) $${\left\{\hat{R_{S}^{\left(‐1\right)}}\right\}}^{'}\left(t\right)=\left(b_{H}‐a_{H}\right){\overset{ˇ}{f}}_{V}^{LL}\left(t\right).$$

Here, ${\overset{ˇ}{f}}_{V}^{LL}$ is a version of the estimator of $f_{V}$ at (3.17) adapted to the estimation of the density of V=R(Υ). It is obtained by replacing everywhere, in (3.17) and in the calculations leading to (3.17), $\hat{H}$ by ${\hat{R}}_{M}$, and thus $m_{\hat{H}}$ and $M_{\hat{H}}$ by ${\hat{R}}_{M}\left(a_{H}\right)$ and ${\hat{R}}_{M}\left(b_{H}\right)$, respectively. See Section 5and Appendix A.3 for more details of implementation.

Finally, we can define a nonparametric estimator of $f_{T}$ on $\left[{\hat{R}}_{M}\left(a_{H}\right),{\hat{R}}_{M}\left(b_{H}\right)\right]$ by

(3.21) $${\hat{f}}_{T}={\left\{\hat{R_{S}^{\left(‐1\right)}}\right\}}^{'}\cdot {\hat{f}}_{X}\left\{{\hat{R}}_{M}^{\left(‐1\right)}\right\}.$$

In Section 5, we will discuss some tail corrections that can be applied in finite samples to improve the practical performance when the data are too sparse.

## 4. Theory

As usual in nonparametric deconvolution problems, the rates of convergence of our estimators depend on the rate of decay of $\left|\phi_{U}(t)\right|$ as |t|→∞. It is standard to distinguish two cases: ordinary smooth, where $\left|\phi_{U}(t)\right|$ decays like $|t{|}^{‐\alpha }$ for some α>1, and supersmooth, where $\left|\phi_{U}(t)\right|$ decays exponentially fast to zero as |t| increases; see for example Stefanski and Carroll (1990) and Fan (1991b). As in Camirand Lemyre, Carroll, and Delaigle (2022), since our theoretical derivations are already very long, we derive convergence rates only in the ordinary smooth case, which is arguably the most interesting theoretically. Indeed, it is well known in the deconvolution literature that in the supersmooth case, the convergence rates of nonparametric estimators are very slow (logarithmic). Unsurprisingly, the rates of our estimators in the supersmooth case are those usual logarithmic rates, but proving this in detail is a purely technical exercise that uses arguments similar to the ordinary smooth case, except that it requires deriving a sequence of rough upper bounds of various terms that are difficult to assess precisely, see Delaigle and Van Keilegom (2021). However, as in Camirand Lemyre, Carroll, and Delaigle (2022), we will see in our numerical work in Section 6 that our estimators work well in practice even in the supersmooth case, despite those slow rates; see Delaigle and Van Keilegom (2021) for a discussion about this in general deconvolution problems.

Likewise, since our proofs are already very long, we derive theoretical properties of our estimators only in the case where $f_{U}$ is known; we will investigate the practical performance of our estimators in the case where $f_{U}$ is unknown in our numerical work in Section 6. Proving consistency of our estimators in the case where $f_{U}$ is unknown and estimated is also a long and purely technical exercise that can be done using our arguments combined with those in Camirand Lemyre, Carroll, and Delaigle (2022). In particular, it requires deriving an almost sure bound for the difference between the non-monotonized versions of our estimators with known and estimated $f_{U}$, which can be done using fairly standard, but tedious, theoretical arguments similar to those used by Camirand Lemyre, Carroll, and Delaigle (2022). As in the standard deconvolution problems studied by Delaigle, Hall, and Meister (2008), in the supersmooth case, estimating $f_{U}$ has a negligible impact on the convergence rates of our estimators; in the ordinary smooth case, the impact is negligible if the functions to estimate are sufficiently smooth.

We establish consistency of the estimator at (3.21), which requires several bandwidths: h used by ${\hat{f}}_{X}$ at (3.14) and $\hat{H}$ at (3.15), a bandwidth for computing ${\hat{f}}_{V}^{LL}$ (see Appendix A.2.3) used to monotonize H, denoted hereafter by $h_{S,H}$, and another one for computing ${\hat{f}}_{V}^{LL}$ used by ${\left\{\hat{R_{S}^{\left(‐1\right)}}\right\}}^{'}$ at (3.20), denoted hereafter by $h_{S,R}$.

In addition to the conditions from Section 2, we use the following assumptions to establish our theoretical results.

### Assumption A

(A1) $\phi_{U}$ has two continuous and bounded derivatives and there exist constants $c_{U}>0$ and α>1 such that ${lim}_{|u|\to \infty }\;|u{|}^{\alpha }\phi_{U}(u)=c_{U}^{‐1}$ and ${lim}_{|u|\to \infty }\;|u{|}^{\alpha +1}\phi_{U}^{'}(u)=‐\alpha c_{U}^{‐1}$.
(A2) K is real, continuous and even, $\phi_{K}$ is supported on [−1, 1] and has four continuous and bounded derivatives. Moreover, $\phi_{K}(0)=1$ and $\phi_{K}(u)=1+O\left(|u{|}^{2}\right)$ as u→0.
(A3) $g=f_{X}H$ and $M=f_{X}H^{2}$ are bounded and have three bounded derivatives.
(A4) As n→∞, (log⁡n)h→0 and $nh^{3+2\alpha }/(\text{log}n{)}^{5}\to \infty$, with α in (A1). For $h_{S}=h_{S,H}$ and $h_{S}=h_{S,R}$, as n→∞, $h_{S}=o\left(h/n^{\epsilon }\right)$ for some ϵ>0 and $nh_{S}^{3}/(\text{log}n{)}^{1/2}\to \infty$; Moreover, λ in the definition of Δ in (3.17) is such that $\lambda \geq {\left\{\text{log}\left(nh_{S}\right)\right\}}^{1/2}/\left(nh_{S}^{3}\right)$, and $\lambda =O\left(h^{2}\right)$ as n→∞.
(A5) ${\left(h^{\left(‐1\right)}*f_{U}\right)}^{'}$ is continuous and bounded away from zero on an open interval on which $f_{X}$ is also bounded away from zero.

Assumption (A1) is used in Camirand Lemyre, Carroll, and Delaigle (2022) and is similar to the one used in Fan (1991a) and Masry (1993); it is satisfied by many distributions. Assumption (A2) is fairly standard in the deconvolution literature; it implies that K is a so-called second-order kernel, which is the most commonly employed type of kernels in practice (higher order kernels tend to produce wiggly estimators). The conditions imposed on g and M in Assumption (A3) are similar to those imposed on $f_{X}$ in Fan (1991a, 1991b) and Masry (1993). Assumption (A4) is similar to conditions usually imposed in the literature on kernel estimators. Reflecting the usual bias-variance tradeoff of those estimators, it states that as n increases, each bandwidth should tend to zero sufficiently fast, but not too fast. As usual for the bandwidth of deconvolution estimators, the rate at which h tends to zero depends on the distribution of U through the parameter α. Assumption (A4) also imposes restrictions on the relative magnitudes of those bandwidths because the nonparametric estimators are combined to compute ${\hat{f}}_{T}$. Finally, in order for the extension of ${\hat{f}}_{V}^{LL}$ to ${\overset{ˇ}{f}}_{V}^{LL}$ be asymptotically negligible, λ needs to be sufficiently small, but not too small. Assumption (A5) is used to derive consistency of our nonparametric estimators of R, $R^{\left(‐1\right)}$ and ${\left\{R^{\left(‐1\right)}\right\}}^{'}$.

The next proposition establishes a uniform strong consistency result for the monotonized estimator ${\hat{H}}_{M}$ of H. Its proof is provided in Appendix E.3.

#### Proposition 4.1.

If Conditions (A1)–(A4) hold, then for any [a,b]⊂I, where I is an open interval on which $f_{X}$ is bounded away from zero, we have, as n→∞,

(4.1) $$\underset{x\in [a,b]}{sup}\;\left|{\hat{H}}_{M}(x)‐H(x)\right|=O_{a.s.}\left\{(\text{log}n{)}^{1/2}{\left(nh^{2\alpha +1}\right)}^{‐1/2}+h^{2}\right\}.$$

Under the conditions of the proposition, ${\hat{H}}_{M}$ has the same uniform strong consistency rate as the standard deconvolution kernel density estimator computed with a second-order kernel in the ordinary smooth case (see e.g., Masry 1993). Like there, the fastest convergence rate in the proposition is of order $(\text{log}n/n{)}^{2/(2\alpha +5)}$ which is obtained by taking $h∼(\text{log}n/n{)}^{1/(2\alpha +5)}$ and any $h_{S,H}$, $h_{S,R}$, and λ in the ranges satisfying Condition (A4), for example, $h_{S,H}∼n^{‐b_{H}},$, $h_{S,R}∼n^{‐b_{R}}$ and $\lambda ∼n^{‐b_{\lambda }}$, for any $b_{H},b_{R}\in (1/(2\alpha +5),(2\alpha +3)/\{3(2\alpha +5)\})$, and any $b_{\lambda }\in \left(2/(2\alpha +5),1‐3\;max\left(b_{H},b_{R}\right)\right)$.

The next theorem establishes a uniform strong consistency result for $f_{T}$. The proof is given in Appendix E.4.

#### Theorem 4.2.

If Conditions (A1)–(A5) hold, then, for any [a,b]⊂I, where I is an open interval on which Condition (A5) is satisfied, we have, as n→∞,

$$\begin{array}{rr} & \;\underset{t\in \left[{\hat{R}}_{M}(a),{\hat{R}}_{M}(b)\right]}{sup}\;\left|{\hat{f}}_{T}(t)‐f_{T}(t)\right|\\ & \;=O_{a.s.}\left[(\text{log}n{)}^{2}\left\{(\text{log}n{)}^{1/2}{\left(nh^{2\alpha +3}\right)}^{‐1/2}+h^{2}\right\}\right]. \end{array}$$

We learn from Theorem 4.2 that the convergence rate of ${\hat{f}}_{T}$ toward $f_{T}$ is slower than that of ${\hat{H}}_{M}$ toward H. This is because estimating $f_{T}$ requires estimating a derivative, ${\left\{R^{\left(‐1\right)}\right\}}^{'}$, and nonparametric estimators of derivatives of a curve usually have slower convergence rates than estimators of the curve itself; see for example Delaigle, Fan, and Carroll (2009) for related errors-in-variables local polynomial estimators of derivatives of a regression curve. The fastest convergence rate in the theorem is of order $(\text{log}n{)}^{2}(\text{log}n/n{)}^{2/(2\alpha +7)}$, which is obtained by taking $h∼(\text{log}n/n{)}^{1/(2\alpha +7)}$ and any $h_{S,H},$ $h_{S,R}$ and λ in the ranges satisfying Condition (A4), for example, $h_{S,H}∼n^{‐b_{H}}$, $h_{S,R}∼n^{‐b_{R}}$ and $\lambda ∼n^{‐b_{\lambda }}$, for any $b_{H},b_{R}\in (1/(2\alpha +7),(2\alpha +5)/\{3(2\alpha +7)\})$, and any $b_{\lambda }\in \left(2/(2\alpha +7),1‐3max\left(b_{H},b_{R}\right)\right)$.

## 5. Numerical Implementation

### 5.1. Outline

Since nonparametric curve estimators are based on local fits, in finite samples they can only be computed reliably where the data are not too sparse. Therefore, it is necessary to make tail adjustments and/or use semiparametric estimators near the tails, neither of which is nonparametrically consistent. In Section 5.2, we define the intervals where we compute our nonparametric estimators and discuss adjustments we use near the endpoints of those intervals. In Section 5.3, we describe our choice of the tuning parameters used by the nonparametric estimators. We summarize all the steps for computing our nonparametric estimators in Algorithms 1 to 6 given in Section 5.3 and Appendices A.2 to A.5. Finally, in Section 5.4, we describe semiparametric estimators we use when estimators are required in the tails themselves.

### 5.2. Estimation Intervals and Endpoints Adjustments

In this section we define the intervals where we compute our nonparametric estimators of $f_{X},$ H, R, $R^{\left(‐1\right)},$ ${\left\{R^{\left(‐1\right)}\right\}}^{'}$ and $f_{T}$, and introduce corrections near the endpoints of those intervals. We use the notation $q_{\tilde{W},\alpha }$ for the α empirical quantile of the ${\tilde{W}}_{ij}$’s.

We start with our estimator ${\hat{f}}_{X}$ of $f_{X}$. Since the regions where it is unreliable usually correspond to those where the ${\tilde{W}}_{ij}$’s are sparse, we compute ${\hat{f}}_{X}$ on the interval $\left[a_{X},b_{X}\right]$ defined as the largest interval containing $\left[q_{\tilde{W},0.025},q_{\tilde{W},0.975}\right]$ where ${\hat{f}}_{X}$ does not take implausibly large values; such values are detected with an approach described in Appendix A.4. Algorithm 5 in Appendix A.4 provides the steps to compute $\left[a_{X},b_{X}\right]$.

Since the monotonization procedure usually smoothes out the fluctuations of the estimator $\hat{H}$ of H, we compute $\hat{H}$ and its monotonized version ${\hat{H}}_{M}$ over a larger interval, $\left[a_{H},b_{H}\right]=\left[q_{\tilde{W},0.001},q_{\tilde{W},0.995}\right]$. We choose a less extreme quantile for $b_{H}$ compared to $a_{H}$ because when x is too large, $\hat{H}(x)$ can repeatedly take the value 1, which can significantly deteriorate the accuracy of ${\hat{H}}_{M}$. Moreover, before computing ${\hat{H}}_{M}$, if $\hat{H}(x)\notin [0,1]$ for some $x\in \left[a_{H},b_{H}\right]$, as can happen near $a_{H}$ and $b_{H}$, we replace $\hat{H}$ by a version $\tilde{H}$ truncated to [0, 1]; see Appendix A.2 and Algorithm 2 there.

We compute ${\hat{R}}_{M}={\hat{H}}_{M}\cdot \left(h^{\left(‐1\right)}*f_{U}\right)$ on $\left[a_{H},b_{H}\right]$ and ${\hat{R}}_{M}^{\left(‐1\right)}$ on $\left[a_{T},b_{T}\right]=\left[{\hat{R}}_{M}\left(a_{H}\right),{\hat{R}}_{M}\left(b_{H}\right)\right]$. We make an endpoint adjustment when estimating ${\left\{R^{\left(‐1\right)}\right\}}^{'}(t)$ on $\left[a_{T},b_{T}\right]$, because ${\left\{R^{\left(‐1\right)}\right\}}^{'}(t)$ is usually quite smooth for t away from the left tail of $f_{T}$, but is less smooth and takes very large values for smaller t. Therefore, we use the first derivative of ${\hat{R}}_{M}^{\left(‐1\right)}(t)$ for t small, and use the smooth estimator ${\left\{\hat{R_{S}^{\left(‐1\right)}}\right\}}^{'}(t)$ at (3.20) for t larger. To transition smoothly between ${\left\{{\hat{R}}_{M}^{\left(‐1\right)}\right\}}^{'}$ for t small and ${\left\{\hat{R_{S}^{\left(‐1\right)}}\right\}}^{'}$ for t larger, we use a weighted sum of these two estimators in a small region $\left[a_{T}^{'},a_{T}^{''}\right]$, where $a_{T}^{'}={\hat{R}}_{M}\left(q_{\tilde{W},0.01}\right)$ and $a_{T}^{''}={\hat{R}}_{M}\left(q_{\tilde{W},0.02}\right)$; letting w be a smooth increasing function such that $w\left(a_{T}^{'}\right)=0$ and $w\left(a_{T}^{''}\right)=1$, we define our nonparametric estimator of ${\left\{R^{\left(‐1\right)}\right\}}^{'}(t)$ for $t\in \left[a_{T},b_{T}\right]=\left[{\hat{R}}_{M}\left(q_{\tilde{W},0.001}\right)\right.$, $\left.{\hat{R}}_{M}\left(q_{\tilde{W},0.995}\right)\right]$ by

(5.1) $${\left\{\hat{R^{\left(‐1\right)}}\right\}}^{'}\left(t\right)=\left\{\begin{array}{ll} {\left\{{\hat{R}}_{M}^{\left(‐1\right)}\right\}}^{'}(t) & \;\text{if}\;t<a_{T}^{'}\\ w(t){\left\{R_{S}^{\left(‐1\right)}\right\}}^{'}(t) & \\ +\{1‐w(t)\}{\left\{{\hat{R}}_{M}^{\left(‐1\right)}\right\}}^{'}(t) & \;\text{if}\;a_{T}^{'}<t<a_{T}^{''}\\ {\left\{\hat{R_{S}^{\left(‐1\right)}}\right\}}^{'}(t) & \;\text{if}\;t\geq a_{T}^{''}. \end{array}\right.$$

In our numerical implementation we took $w(t)=\left\{{\left(a_{T}^{''}‐a_{T}^{'}\right)}^{2}‐\right.{\left.{\left(t‐a_{T}^{''}\right)}^{2}\right\}}^{2}/{\left(a_{T}^{''}‐a_{T}^{'}\right)}^{4}$.

Computing ${\hat{f}}_{T}(t)$ for all $t\in \left[a_{T},b_{T}\right]$ from the formula in (3.21) would require computing the value of ${\hat{f}}_{X}\left\{{\hat{R}}_{M}^{\left(‐1\right)}(t)\right\}$ for those t. This may not always be feasible since ${\hat{f}}_{X}$ is only computed over $\left[a_{X},b_{X}\right]$. To account for cases where ${\hat{R}}_{M}^{\left(‐1\right)}(t)$ falls outside of this interval, we adjust ${\hat{f}}_{T}(t)$ as follows:

(5.2) $${\hat{f}}_{T}(t)=\left\{\begin{array}{ll} {\left\{\hat{R^{\left(‐1\right)}}\right\}}^{'}(t){\hat{f}}_{X}\left\{{\hat{R}}_{M}^{\left(‐1\right)}(t)\right\} & \;\text{if}\;{\hat{R}}_{M}^{\left(‐1\right)}(t)\in \left[a_{X},b_{X}\right]\\ {\left\{\hat{R^{\left(‐1\right)}}\right\}}^{'}(t){\hat{f}}_{X,SP}\left\{{\hat{R}}_{M}^{\left(‐1\right)}(t)\right\} & \text{otherwise}, \end{array}\right.$$

where ${\hat{f}}_{X,SP}$ denotes Camirand Lemyre, Carroll, and Delaigle’s (2022) semiparametric estimator of $f_{X}$. Finally, in some cases, ${\left\{\hat{R^{\left(‐1\right)}}\right\}}^{'}(t)$ can be unreliable for values of t near $a_{T}$ or $b_{T}$, making ${\hat{f}}_{T}$ take implausibly large values. To deal with this issue, for any $t<{\hat{R}}_{M}\left(q_{\tilde{W},0.025}\right)$ and $t>{\hat{R}}_{M}\left(q_{\tilde{W},0.975}\right)$ where ${\hat{f}}_{T}(t)$ (computed as in (5.2)) is implausibly large, we replace ${\left\{\hat{R^{\left(‐1\right)}}\right\}}^{'}(t)$ in (5.2) by ${\left\{\hat{R_{S}^{\left(‐1\right)}}\right\}}^{'}(t)$ or ${\left\{{\hat{R}}_{SP}^{\left(‐1\right)}\right\}}^{'}(t)$ from Section 5.4; see Algorithm 6 in Appendix A.5 for details.

### 5.3. Selection of Tuning Parameters

Our nonparametric estimators $\hat{H}$ and ${\hat{f}}_{X}$ require the choice of a bandwidth h. We use the 2-stage plug-in bandwidth $h_{PI}$ of Delaigle and Gijbels (2002, 2004) applied to the observed $W_{ij}$’s. We tried a more sophisticated SIMEX approach similar to that in Delaigle, Hall, and Meister (2008), adapted to the estimation of $f_{T}$, but it was much more time consuming while not improving the results.

To compute ${\left\{\hat{R_{S}^{\left(‐1\right)}}\right\}}^{'}$ and the monotonized version ${\hat{H}}_{M}$ of $\hat{H}$, we need to compute ${\overset{ˇ}{f}}_{V}^{LL}$ at (3.20) and (3.17). While this estimator only has second order impact on asymptotic results, to compute it in practice we need to choose a data-driven bandwidth. In the case of ${\left\{\hat{R_{S}^{\left(‐1\right)}}\right\}}^{'}$, we used Geenens (2014) WLSCV1 nearest neighbor (NN) bandwidth. In the case of ${\hat{H}}_{M}$, we found that bandwidth too small and we developed an alternative NN bandwidth called L2KDE; see Appendices A.2-A.3 for details. To compute ${\overset{ˇ}{f}}_{V}^{LL}$ at (3.17), we need to extend ${\hat{f}}_{V}^{LL}$ to a boundary region determined by a parameter λ. Our theoretical results show that we can choose λ within a broad range, reflecting the relative robustness of the procedure to the choice of λ. Therefore, to avoid introducing an additional data-driven criterion that would add unnecessary complexity, we set λ=0.01, making the length of the boundary region 2% that of the support of ${\overset{ˇ}{f}}_{V}^{LL}$. See Algorithms 3 and 4 in Appendices A.2-A.3, where we provide step by step instructions for implementing ${\hat{f}}_{V}^{LL}$ and ${\overset{ˇ}{f}}_{V}^{LL}$ in practice. Finally, to compute ${\hat{f}}_{X}$ and $\hat{H}$, we take the kernel K with Fourier transform $\phi_{K}(t)={\left(1‐t^{2}\right)}^{3}$. I{t∈[‐1,1]}, which is commonly used in the deconvolution literature.

We wrote Matlab code to compute our estimators, except for the monotization procedure, for which we adapted Geenens (2014) R code to our case, using approaches similar to him to deal with numerical issues; see Appendix A.2.3.

Algorithm 1 summarizes the steps for computing our nonparametric estimators. It uses Algorithms 3-6 provided in Appendices A.2-A.5.

**Algorithm 1:** Details for computing nonparametric estimators.

1. Compute ${\hat{f}}_{X}$ at (3.14) on $\left[a_{X},b_{X}\right]$ obtained by Algorithm 5, with Delaigle and Gijbels (2002, 2004) plug-in $h=h_{PI}$ applied to the ${\tilde{W}}_{ij}$’s; replace ${\hat{f}}_{X}(x)<0$ by 0. 2. On $\left[a_{H},b_{H}\right]=\left[q_{\tilde{W},0.001},q_{\tilde{W},0.995}\right]$, using $h=h_{PI}$, compute $\hat{H}$ at (3.15), its monotonized version ${\hat{H}}_{M}$ as in Algorithm 3, and take ${\hat{R}}_{M}={\hat{H}}_{M}\cdot \left(h^{\left(‐1\right)}*f_{U}\right)$. 3. Let $a_{T}={\hat{R}}_{M}\left(a_{H}\right),$ $b_{T}={\hat{R}}_{M}\left(b_{H}\right),$ $a_{T}^{'}={\hat{R}}_{M}\left(q_{\tilde{W},0.01}\right)$ and $a_{T}^{''}={\hat{R}}_{M}\left(q_{\tilde{W},0.02}\right)$; on $\left[a_{T},b_{T}\right]$, compute RM ${\hat{R}}_{M}^{\left(‐1\right)},$ ${\left\{{\hat{R}}_{M}^{\left(‐1\right)}\right\}}^{'}$, ${\left\{\hat{R_{S}^{\left(‐1\right)}}\right\}}^{'}$ as in Algorithm 4, and let ${\left\{\hat{R^{\left(‐1\right)}}\right\}}^{'}(t)$ as at (5.1), with $w(t)={\left\{{\left(a_{T}^{''}‐a_{T}^{'}\right)}^{2}‐{\left(t‐a_{T}^{''}\right)}^{2}\right\}}^{2}/{\left(a_{T}^{''}‐a_{T}^{'}\right)}^{4}$. 4. For $t\in \left[a_{T},b_{T}\right]$, compute ${\hat{f}}_{T}(t)$ as at (5.2), with ${\hat{f}}_{X,SP}$ the semiparametric estimator of Camirand Lemyre, Carroll, and Delaigle (2022). 5. On $\left[a_{T},b_{T}\right]\setminus \left[{\hat{R}}_{M}\left(q_{\tilde{W},0.025}\right),{\hat{R}}_{M}\left(q_{\tilde{W},0.975}\right)\right]$, if ${\hat{f}}_{T}$ is implausibly large, replace ${\left\{\hat{R^{\left(‐1\right)}}\right\}}^{'}$ in (5.2) by ${\left\{\hat{R_{S}^{\left(‐1\right)}}\right\}}^{'}$ or ${\left\{{\hat{R}}_{SP}^{\left(‐1\right)}\right\}}^{'}$ at (5.3), as described in Algorithm 6.

### 5.4. Semiparametric Estimators in the Tails

If we need to estimate $f_{T}(t)$ for $t\notin \left[a_{T},b_{T}\right]$, we use a semiparametric approach. For $t<a_{T}$, we estimate $R^{\left(-1\right)}(t)$ by ${\hat{R}}_{SP}^{\left(‐1\right)}(t)$, the inverse of ${\hat{R}}_{SP}={\hat{H}}_{SP}\cdot \left(h^{\left(‐1\right)}*f_{U}\right)$, where ${\hat{H}}_{\text{SP}\;}$ denotes Camirand Lemyre, Carroll, and Delaigle’s (2022) semiparametric estimator, which uses a parametric model for H; then we estimate ${\left\{R^{\left(‐1\right)}\right\}}^{'}(t)$ by

(5.3) $${\left\{{\hat{R}}_{SP}^{\left(‐1\right)}\right\}}^{'}(t)=1/{\hat{R}}_{SP}^{'}\left\{{\hat{R}}_{SP}^{\left(‐1\right)}(t)\right\}$$

and define ${\hat{f}}_{T}(t)$ as in (5.2) but replacing there ${\hat{R}}_{M}^{\left(‐1\right)}$ and ${\left\{\hat{R^{\left(‐1\right)}}\right\}}^{'}$ by ${\hat{R}}_{SP}^{\left(‐1\right)}$ and ${\left\{{\hat{R}}_{SP}^{\left(‐1\right)}\right\}}^{'}$, respectively. For $t>b_{T}$, since $f_{T}(t)$ and (5.2) are close to zero, we simply extrapolate the estimators ${\hat{R}}_{M}^{\left(‐1\right)}$ and ${\left\{\hat{R^{\left(‐1\right)}}\right\}}^{'}(t)$ linearly from $\left[a_{T},b_{T}\right]$, and define ${\hat{f}}_{T}(t)$ as in (5.2) but replacing there ${\hat{R}}_{M}^{\left(‐1\right)}$ and ${\left\{\hat{R^{\left(‐1\right)}}\right\}}^{'}$ by their extrapolated versions.

## 6. Simulations

We applied our nonparametric estimator of ${\hat{f}}_{T}$ of $f_{T}$ to various configurations. To illustrate the advantages of nonparametric techniques, we compared it with Camirand Lemyre, Carroll, and Delaigle’s (2022) semiparametric estimator ${\hat{f}}_{T,SP}$ from Section 5.4, and with a parametric maximum likelihood (ML) estimator ${\hat{f}}_{T,ML}$ introduced in Appendix A.6.

Following (3.1) and (2.5), $f_{T}$ depends on $f_{X},$ H, $f_{U}$, and h. For h we used the log transform, which is the most common in applications. For H, we considered two models where H was monotone: $H_{1}\left(x;\beta_{0},\beta_{1}\right)=\text{exp}\left(\beta_{0}+\beta_{1}x\right)/\left\{1+\text{exp}\left(\beta_{0}+\beta_{1}x\right)\right\}$ and $H_{2}\left(x;\mu_{1},\sigma_{1},\mu_{2},\sigma_{2}\right)=0.35+0.35\Phi_{\mu_{1},\sigma_{1}}(x)+0.3\Phi_{\mu_{2},\sigma_{2}}(x)$, where $\Phi_{\mu ,\sigma }$ denotes the cdf of a $N\left(\mu ,\sigma^{2}\right).H_{1}$ is a standard logistic curve often employed in applications and can approximate reasonably well many probability curves corresponding to unimodal distributions; it can itself often be well approximated by $\Phi_{\mu ,\sigma }$, another standard curve. Let ${\hat{f}}_{T,{SP}_{H_{1}}}$ (resp., ${\hat{f}}_{T,{SP}_{\Phi }}$ ) denote ${\hat{f}}_{T,\text{SP}}$ computed under the assumption that H is modeled by $H_{1}\left(\cdot ;\beta_{0},\beta_{1}\right)$ (resp., $\Phi_{\mu ,\sigma }$). In the case where $H=H_{1}$, we computed two versions of ${\hat{f}}_{T,SP}:{\hat{f}}_{T,{SP}_{H_{1}}}$ with unknown $\beta_{0}$ and $\beta_{1}$, based on the correct model for H, and ${\hat{f}}_{T,{SP}_{\Phi }}$ with unknown μ and σ, based on a misspecified model for H. The curve $H_{2}$ has more complex features which are difficult to guess in applications, where one would more often use standard functions such as $H_{1}$. Therefore, when $H=H_{2}$ we computed only ${\hat{f}}_{T,{SP}_{H_{1}}}$ with unknown $\beta_{0}$ and $\beta_{1}$, based on a misspecified model for H.

To examine the performance of ${\hat{f}}_{T}$ when monotonicity of H is slightly violated, we also considered two non-monotone variations of $H_{1}$ and $H_{2}:H_{3}\left(x;\beta_{0},\beta_{1},\alpha_{1},\alpha_{2}\right)=H_{1}\left(x;\beta_{0},\beta_{1}\right)\cdot g\left(x,\alpha_{1},\alpha_{2}\right)$ and $H_{4}\left(x;\mu_{1},\sigma_{1},\mu_{2},\sigma_{2},\alpha_{1},\alpha_{2}\right)=H_{2}\left(x;\mu_{1},\sigma_{1},\mu_{2},\sigma_{2}\right)\cdot g\left(x,\alpha_{1},\alpha_{2}\right)$, where $g\left(x,\alpha_{1},\alpha_{2}\right)=1+\alpha_{1}exp\left\{‐{\left(x‐\alpha_{2}\right)}^{2}\right\}$ is a function that is close to 1, except in a small region around $\alpha_{2}$ where it introduces a small positive (if $\alpha_{1}>0$) or negative (if $\alpha_{1}<0$ ) dip. When $H=H_{3}$ and $H=H_{4}$, the version of ${\hat{f}}_{T,SP}$ we computed was ${\hat{f}}_{T,{SP}_{H_{1}}}$ with unknown $\beta_{0}$ and $\beta_{1}$, based on a misspecified model for H.

We considered the following models:

(1) $f_{X}=pf_{1}+(1‐p)f_{2}$ where, for $j=1,2,f_{j}$ is the density of $\text{log}G_{j}$, with $G_{1}∼\Gamma (9,0.5),$ $G_{2}∼\Gamma (3.5,0.4),$ p=0.3,Γ(α,β) is a Gamma distribution with shape and scale parameters α and β, and $H=H_{1}(\cdot ;2,1),$ $H_{2}(\cdot ;‐1.7,0.4,1.4,0.5)$ or $H_{3}(\cdot ;2,1,‐0.1,2)$;

(2) $f_{X}=pf_{1}+(1‐p)f_{2}$ where, for j=1,2, $f_{j}$ is the density of $N_{j}$, with $N_{1}∼N\left(1.3,0.4^{2}\right),$ $N_{2}∼N\left(3,0.4^{2}\right),$ p=0.3, and $H=H_{1}(\cdot ;0,1),$ $H_{2}(\cdot ;‐0.2,0.4,2.4,0.5),$ $H_{3}(\cdot ;0,1,0.2,1)$ or $H_{4}(\cdot ;‐0.2,\;0.4,\;2.4,\;0.5,‐0.05,\;1.6)$;

(3) $X∼N\left(3,0.75^{2}\right)$ and $H=H_{1}(\cdot ;‐2,1),$ $H_{2}(\cdot ;0.8,0.4,3.4,0.5)$ or $H_{4}(\cdot ;0.8,\;0.4,\;3.4,\;0.5,\;0.05,1.6)$.

The first two densities of X are bimodal and the third is a simpler unimodal density. All three cases have a right-skewed density $f_{T}$. We considered U with either a normal (when $H=H_{1}$ or $H_{2}$) or a Laplace (when $H=H_{1},H_{2},H_{3}$ or $H_{4}$) distribution, and with a noise to signal ratio NSR=varU/varX equal to 10% or 20%. When computing the estimators, we assumed that $f_{U}$ and $\phi_{U}$ were unknown and estimated them as in Appendix A.1.

For α∈[0,1], let $q_{X,\alpha }$ denote the α quantile of the distribution of X.. We estimated $f_{T}$ on the grid $G_{T}$ of 5000 points in the interval $\left[t_{L},t_{U}\right]$ obtained by taking t=R(x) for each x in a grid $G_{X}$ of 5000 equispaced points in the interval $\left[x_{L},x_{U}\right]=\left[q_{X,0.001},q_{X,0.999}\right]$. In each case we computed our nonparametric estimator $f_{T}$ of $f_{T}$ implemented as in Section 5.3, and compared it with Camirand Lemyre, Carroll, and Delaigle’s (2022) semiparametric estimator ${\hat{f}}_{T,SP}$. We also computed the parametric estimator ${\hat{f}}_{T,ML}$ from Appendix A.6, assuming in each case that $∼N\left(\mu_{X},\sigma_{X}^{2}\right),$ $H=H_{1}\left(\cdot ;\beta_{0},\beta_{1}\right)$, and $U∼N\left(0,\sigma_{U}^{2}\right)$ with $\sigma_{U}^{2}$ estimated by half the variance of the ${\tilde{W}}_{ij}‐{\tilde{W}}_{ij}$’s for which $W_{ij}>0$ and $W_{ij^{'}}>0$. We intentionally took the intervals [$x_{L},x_{U}$] and $\left[t_{L},t_{U}\right]$ very large to illustrate the data sparsity problem near the tails discussed earlier; we will see that ${\hat{f}}_{X}$ and ${\hat{f}}_{T,SP}$ are often unreliable in the left tail.

For each combination of $f_{X},$ $f_{U},$ H and NSR, we generated 1000 samples of sizes n=250,500, or 1000, and took J=2 or 4 replications per individual. Let ${\overset{˘}{f}}_{T}$ denote any of the estimators of $f_{T}$ we considered. For each estimator and each sample, we t computed the integrated squared error $ISE\;=\int_{t_{L}}^{t_{U}}\;\left\{{\overset{˘}{f}}_{T}(t)‐\right.{\left.f_{T}(t)\right\}}^{2}\;dx$. In the tables, for each configuration, we report the first three quartiles of these 1000 ISEs.

Tables 1–3 (for $H_{1}$ and $H_{2}$), and B.1 to B.3 in Appendix B (for $H_{3}$ and $H_{4}$) show that overall, when $H=H_{1}$ or its slightly nonmonotone version $H_{3}$, our nonparametric estimator ${\hat{f}}_{T}$ gave results similar to Camirand Lemyre, Carroll, and Delaigle’s (2022) semiparametric estimator ${\hat{f}}_{T,SP}$. An exception is the simpler model (3) with $H=H_{1}$ and J=2, where ${\hat{f}}_{T,\text{SP}}$ worked markedly better, even if the parametric model for H was misspecified to $\Phi_{\mu ,\sigma }$. This is because $\Phi_{\mu ,\sigma }$ can be a good approximation to $H_{1}$, and so ${\hat{f}}_{T,{SP}_{H_{1}}}$ and ${\hat{f}}_{T,{SP}_{\Phi }}$ usually performed similarly. For the more complex function $H=H_{2}$ and its slightly nonmonotone version $H_{4}$, only the main trend of H could be captured by the incorrectly specified $H_{1}\left(\cdot ;\beta_{0},\beta_{1}\right)$, and our nonparametric estimator ${\hat{f}}_{T}$ significantly outperformed ${\hat{f}}_{T,{SP}_{H_{1}}}$ in all models. Unsurprisingly, in model (3) with $H=H_{1}$ where the full parametric model used by the parametric estimator was correctly specified, ${\hat{f}}_{T,\text{ML}}$ outperformed all other estimators. The estimator ${\hat{f}}_{T,\text{ML}}$ also performed reasonably well in model (1) when $H=H_{2}$ or $H_{3}$, but was considerably outperformed by our nonparametric estimator ${\hat{f}}_{T}$ in all other cases. ${\hat{f}}_{T,\text{ML}}$ performed very poorly in the more complex model (2), but in model (1) when $H=H_{2}$ or $H_{3}$ and model (3) with $H=H_{2}$ or H_4_, it outperformed Camirand Lemyre, Carroll, and Delaigle’s (2022) estimator ${\hat{f}}_{T,{SP}_{H_{1}}}$. Overall, ${\hat{f}}_{T}$ was quite competitive with the other estimators in all cases, and except for model (3) with $H=H_{1}$ discussed above, it worked either comparably to, or considerably better than, the other estimators. In model (2), it even outperformed the semiparametric estimator ${\hat{f}}_{T,SP}$ with correctly specified model for H, perhaps owing to beneficial cancellations from the nonparametric estimators of the numerator and the denominator of ${\hat{f}}_{T}$, a common phenomenon in nonparametric curve estimation problems involving a ratio. The good performance ${\hat{f}}_{T}$ in the non monotone examples $H_{3}$ and $H_{4}$ highlights its flexibility compared to the other approaches which not only assume monotonicity of H but also impose a parametric form on it.

**Figure 1.**
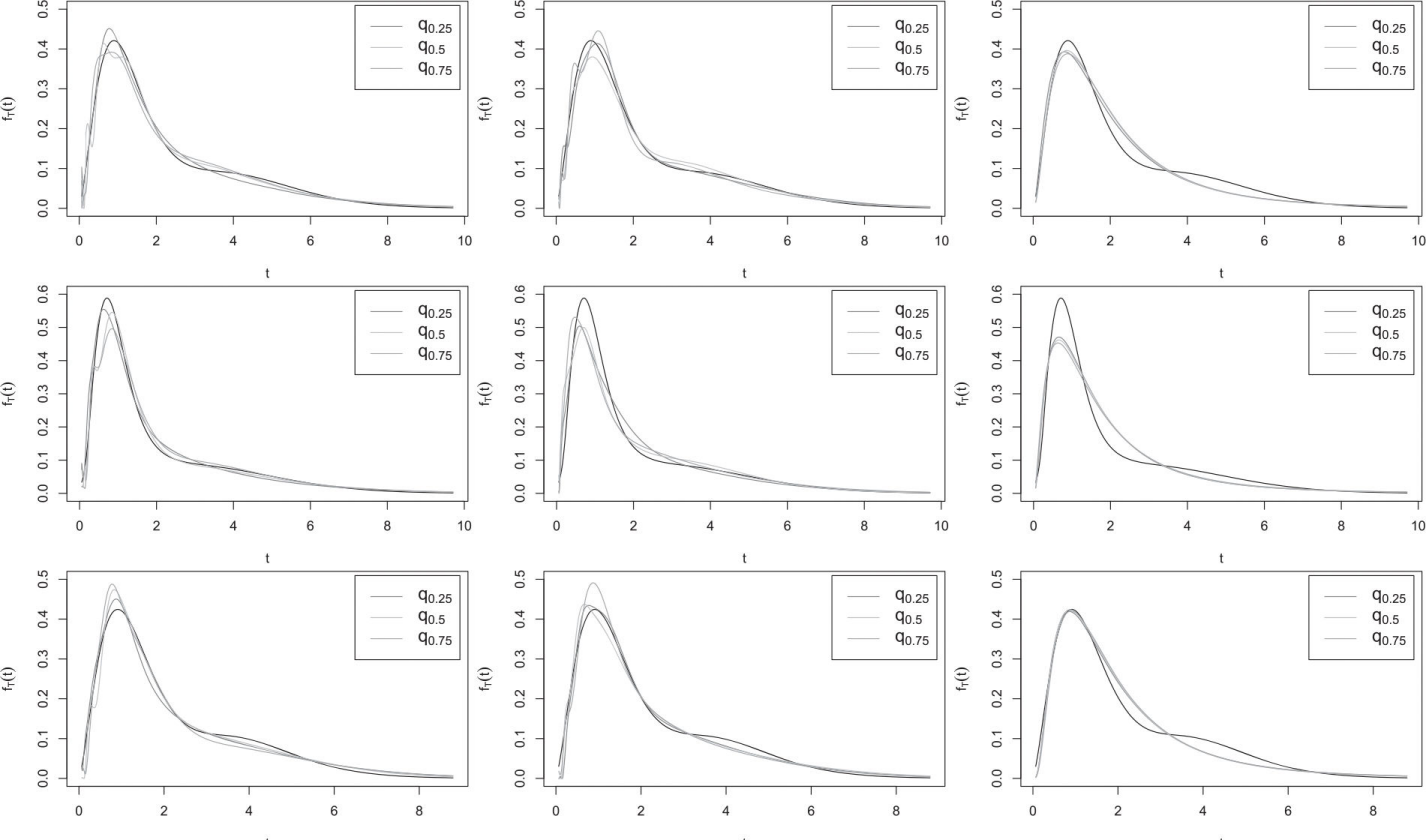
Estimated curves corresponding to the quantiles $q_{0.25},$ $q_{0.5}$, and $q_{0.75}$ of the ISE of ${\hat{f}}_{T}$ (left), ${\hat{f}}_{SP,H_{1}}$ (middle) or ${\hat{f}}_{ML}$ (right) computed from 1000 samples of size n=1000 from model (1) $H=H_{1}$ and normal $f_{U}$ (row 1), $H=H_{2}$ and normal $f_{U}$ (row 2) or $H=H_{3}$ and Laplace $f_{U}$ (row 3), with NSR=10% and J=2. The solid line shows the true $f_{T}$.

**Figure 2.**
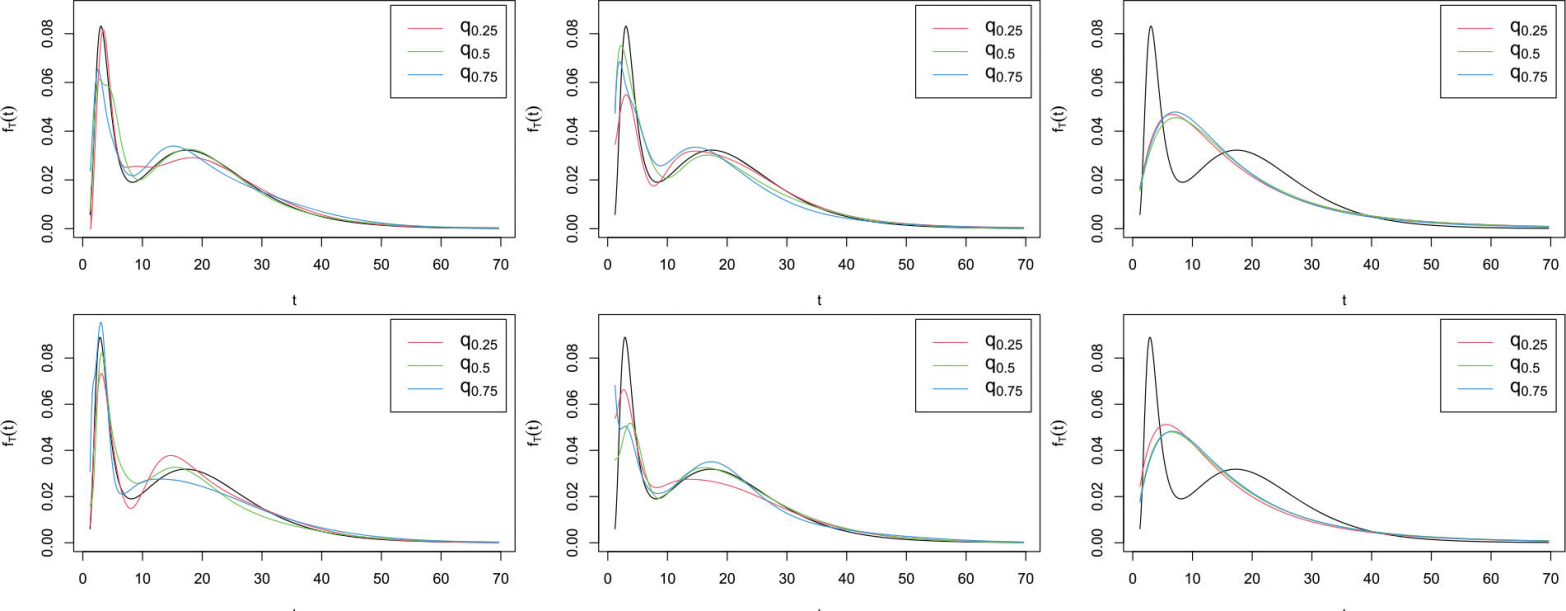
Estimated curves corresponding to the quantiles $q_{0.25},$ $q_{0.5}$, and $q_{0.75}$ of the ISE of ${\hat{f}}_{T}$ (left), ${\hat{f}}_{{SP}_{H_{1}}}$ (middle) or ${\hat{f}}_{ML}$ (right) computed from 1000 samples of size n=500 from model (2) with $H=H_{2}$ (row 1) or $H_{4}$ (row 2), NSR=20% and Laplace $f_{U}$, when J=4. The solid line shows the true $f_{T}$.

**Figure 3.**
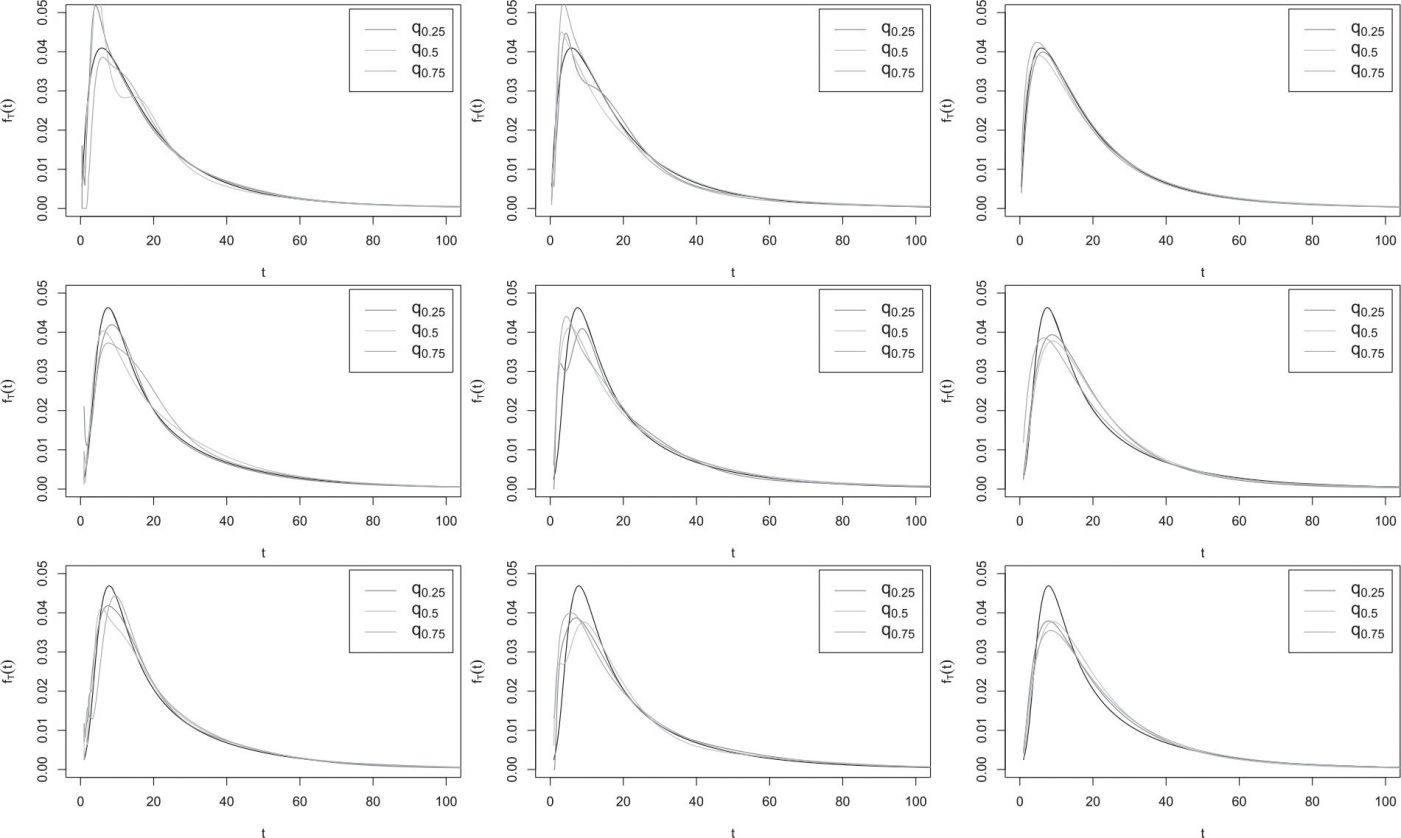
Estimated curves corresponding to the quantiles $q_{0.25},$ $q_{0.5}$, and $q_{0.75}$ of the ISE of ${\hat{f}}_{T}$ (left), ${\hat{f}}_{{SP}_{H_{1}}}$ (middle) or ${\hat{f}}_{ML}$ (right) computed from 1000 samples of size n=500 from model (3) with $H=H_{1}$ and normal $f_{U}$ (row 1), $H=H_{2}$ and normal $f_{U}$ (row 2), or $H=H_{4}$ and Laplace $f_{U}$ (row 3), with NSR=10% and J=2. The solid line shows the true $f_{T}$.

To illustrate the results visually, in Figures 1–3, we compare, in a few cases, our nonparametric estimator ${\hat{f}}_{T}$ with the semiparametric and parametric estimators ${\hat{f}}_{T,{SP}_{H_{1}}}$ and ${\hat{f}}_{T,ML}$, both computed under the assumption that the model for H was $H_{1}\left(\cdot ;\beta_{0},\beta_{1}\right)$. For each estimator depicted in the figures, we show the estimated curves corresponding to the first, second and third quartiles ($q_{0.25},$ $q_{0.5}$ and $q_{0.75}$) of the 1000 ISEs obtained from the simulations. In general, when the true model for H was $H_{1}$ or its slightly nonmonotone version $H_{3},$ ${\hat{f}}_{T}$ performed reasonably well compared to ${\hat{f}}_{T,S_{H_{1}}}$. When the true model for H was $H_{2}$ or its slightly nonmonotone version $H_{4},$ ${\hat{f}}_{T,{SP}_{H_{1}}}$ was usually able to capture the main features of $f_{T}$ but tended to be much more biased than ${\hat{f}}_{T}$. For example, in Figure 2, when $H=H_{2}$ or $H_{4}$, ${\hat{f}}_{T,{SP}_{H_{1}}}$ appears to underestimate both the height and the location of the left-most peak of $f_{T}$, to the extent that the left side of the estimated peak is not captured in the figures; see Figure 3 for similar results. As expected, the parametric estimator ${\hat{f}}_{T,ML}$ performed more poorly in many cases, but considerably outperformed all other estimators in model (3) with $H=H_{1}$ (first row of Figure 3), where the parametric assumptions were all correctly specified.

To summarize, in our simulation study, the nonparametric estimator ${\hat{f}}_{T}$ generally performed well across all scenarios and was competitive with both the semiparametric estimator ${\hat{f}}_{T,SP}$ and the parametric estimator ${\hat{f}}_{T,ML}$, particularly when the parametric assumptions used by ${\hat{f}}_{T,SP}$ and ${\hat{f}}_{T,ML}$ deviated significantly from the simulated models. In such cases, ${\hat{f}}_{T,SP}$ and ${\hat{f}}_{T,ML}$ sometimes performed poorly, while ${\hat{f}}_{T}$ maintained reasonable performance. This suggests that large discrepancies between ${\hat{f}}_{T}$ and either ${\hat{f}}_{T,\text{SP}}$ or ${\hat{f}}_{T,\text{ML}}$ may indicate issues with model specification in the latter estimators. However, as expected, when parametric assumptions were all correctly specified, the parametric estimator ${\hat{f}}_{T,ML}$ outperformed all other estimators.

## 7. Application to EATS Data

We applied our technique to fruit consumption data from the Eating at America’s Table Study (EATS, Subar et al. 2001). In this dataset, there are J=4 repeated measurements of W, the servings of total fruit consumption standardized by energy intake (i.e., divided by 1000 kilocalories) obtained from four 24 hr recalls for n=965 individuals. Our goal was to estimate the density $f_{T}$ of long term standardized intake, where as often in nutrition, we took h=log.

We estimated $f_{T}$ using the three estimators considered in Section 6: our new nonparametric estimator ${\hat{f}}_{T}$ with $f_{U}$ and $\phi_{U}$ estimated as in Appendix A.1, ${\hat{f}}_{T,S_{H_{1}}}$ from the previous section, that is, Camirand Lemyre, Carroll, and Delaigle’s (2022) semiparametric estimator ${\hat{f}}_{T,\text{SP}}$ computed under the parametric assumption that H is logistic, and with $f_{U}$ and $\phi_{U}$ estimated as in Appendix A.1, and the parametric estimator ${\hat{f}}_{T,ML}$ described in Appendix A.6 assuming that H is logistic, $X∼N\left(\mu_{X},\sigma_{X}^{2}\right)$ and $U∼N\left(0,\sigma_{U}^{2}\right)$ with $\sigma_{U}^{2}$ estimated by half the variance of the ${\tilde{W}}_{ij}‐{\tilde{W}}_{ij^{'}}$’s for which $W_{ij}>0$ and $W_{ij^{'}}>0$.

As noted in Camirand Lemyre, Carroll, and Delaigle (2022), the ML estimator relies heavily on the transformed data and the errors to be both normally distributed. Like them, to make the transformed data closer to normal, we followed common practice in nutritional epidemiology and censored values of (non standardized) servings of total fruit consumption that were implausibly small (smaller than 0.02) to zero, which resulted in a more reasonable estimator.

**Figure 4.**
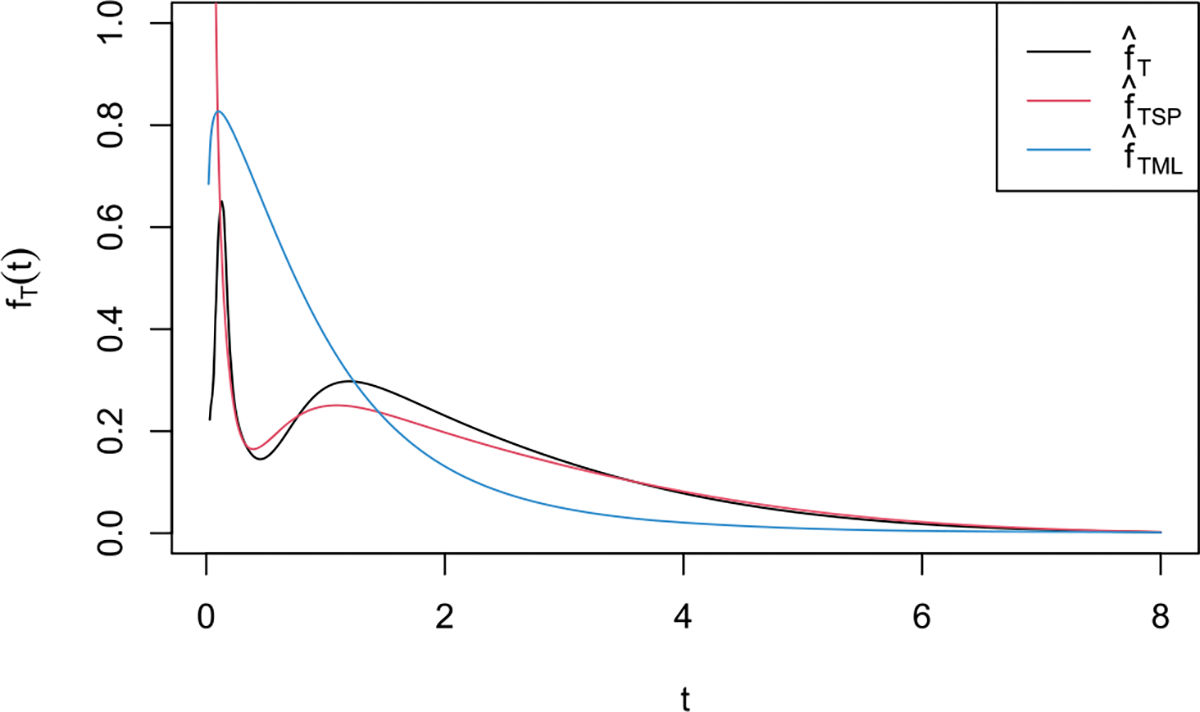
Estimators ${\hat{f}}_{T},$ ${\hat{f}}_{T,SP}$, and ${\hat{f}}_{T,P}$ of the density of usual total fruit intake standardised by energy intake.

The three estimators of $f_{T}$ are shown in Figure 4. As discussed earlier, ${\hat{f}}_{T}$ and ${\hat{f}}_{T,\text{SP}}$ are not reliable for t very small. Reflecting this, ${\hat{f}}_{T}$ became unreliable for t≤0.02, and so we only show the estimators for t>0.02. Things were worse for ${\hat{f}}_{T,SP}$, which took implausibly large values for t as large as 0.1 so that we truncated the vertical axis of the figure to 1.1 to make the figure readable. In this example, ${\hat{f}}_{T}$ detected a first mode that seems to correspond to individuals with a very small usual fruit intake, and a second more widespread mode that seems to correspond to individuals with larger usual fruit intake. The semiparametric estimator ${\hat{f}}_{T,\text{SP}}$ detected only one mode, and took on implausibly large values for t<0.1. Because it was restricted by the normality and logistic assumptions, the parametric estimator ${\hat{f}}_{T,\text{ML}}$ was only able to capture one mode, suggesting that the normality assumption on X is not a very good approximation to the truth in this case. This is similar to the results for our simulated model (2), where the true $f_{X}$ was bimodal but we pretended it was normal and ${\hat{f}}_{T,ML}$ behaved very poorly; see Figure 2. These results illustrate that the restrictions imposed by parametric models may fail to capture the overall behavior of the true density $f_{T}$, highlighting the importance of nonparametric alternatives.

## Supplementary Material

Supp 1

Supplementary materials for this article are available online. Please go to www.tandfonline.com/r/JASA.

The Supplementary Material includes an appendix with technical details, Matlab and R code for computing our estimator, and the EATS data analyzed in this article.

## Figures and Tables

**Table 1.** Simulation results for model (1).

True H	J	NSR=10%			NSR=20%			n=1000
		${\overset{ˇ}{f}}_{T}$	n=250	n=500	n=1000	n=250	n=500	

U Laplace								
$H_{1}$	2	NP	42[28,65]	30[20,41]	19[14,29]	47[33,72]	33[23,47]	23[16,33]
		${SP}_{\Phi }$	43[28,66]	28[19,41]	19[13,26]	48[33,73]	33[23,47]	22[16,30]
		${SP}_{H_{1}}$	44[28,66]	28[19,41]	18[13,26]	49[32,73]	33[23,47]	22[16,31]
		ML	50[43,60]	45[41,51]	42[40,46]	49[42,60]	44[39,50]	41[39,45]
	4	NP	40[27,60]	26[18,38]	17[12,24]	42[28,63]	29[19,42]	18[13,27]
		${SP}_{\Phi }$	44[29,68]	30[20,41]	18[13,25]	54[33,82]	37[25,52]	23[16,33]
		${SP}_{H_{1}}$	44[29,67]	29[20,40]	18[12,25]	54[33,80]	36[24,51]	23[16,32]
		ML	47[42,58]	43[40,49]	42[40,46]	46[41,56]	42[39,47]	41[39,44]
$H_{2}$	2	NP	107[66,160]	73[48,104]	50[34,69]	121[76,180]	85[57,123]	62[41,86]
		${SP}_{H_{1}}$	163[110,240]	137[99,183]	115[86,148]	174[117,253]	143[103,194]	122[91,159]
		ML	152[127,182]	144[128,163]	139[128,150]	149[124,179]	141[124,160]	135[124,147]
	4	NP	73[48,108]	47[30,70]	30[21,42]	83[53,117]	56[37,81]	37[26,51]
		${SP}_{H_{1}}$	181[116,279]	153[107,212]	138[94,179]	202[129,300]	173[122,238]	153[108,200]
		ML	138[122,159]	134[122,148]	130[122,139]	134[118,154]	130[118,144]	127[118,135]
U normal								
$H_{1}$	2	NP	45[31,66]	30[21,44]	22[16,31]	53[35,81]	37[26,55]	29[20,42]
		${SP}_{\Phi }$	46[31,66]	30[21,43]	21[15,30]	53[35,79]	38[27,54]	29[20,40]
		${SP}_{H_{1}}$	46[31,66]	30[21,44]	21[15,30]	54[35,81]	38[27,54]	28[20,40]
		ML	50[43,62]	44[41,51]	42[40,46]	49[42,62]	44[40,50]	41[39,45]
	4	NP	42[28,62]	28[19,40]	20[14,27]	48[30,70]	34[24,49]	24[17,35]
		${SP}_{\Phi }$	48[31,71]	32[23,47]	23[16,34]	69[45,104]	52[34,79]	40[28,58]
		${SP}_{H_{1}}$	47[32,70]	31[22,46]	23[16,32]	68[44,103]	50[34,76]	39[27,56]
		ML	47[42,57]	44[41,50]	41[40,45]	46[41,56]	42[39,49]	40[38,44]
$H_{2}$	2	NP	114[73,171]	81[54,113]	57[39,80]	135[88,202]	99[69,142]	78[54,109]
		${SP}_{H_{1}}$	177[118,255]	144[105,194]	122[93,159]	192[127,267]	152[110,208]	129[93,168]
		ML	152[129,182]	144[127,162]	140[128,153]	150[126,179]	141[124,159]	137[124,149]
	4	NP	78[50,113]	52[34,75]	35[25,48]	94[62,131]	67[46,94]	50[35,69]
		${SP}_{H_{1}}$	189[124,287]	160[112,229]	147[113,189]	235[154,337]	203[143,281]	183[137,240]
		ML	141[122,161]	133[121,147]	131[123,140]	137[119,159]	130[118,144]	128[119,137]

NOTE: The numbers show 10^4^ × median [1st quartile, 3rd quartile] of 1000 ISEs for our nonparametric estimator ${\hat{f}}_{T}$ (NP), the semiparametric estimator ${\hat{f}}_{T,SP_{H_{1}}}$ that assumes $H=H_{1}\left({SP}_{H_{1}}\right)$ and its version ${\hat{f}}_{T,{SP}_{\Phi }}$ that assumes $H=\Phi \left({SP}_{\Phi }\right)$, and the parametric estimator ${\hat{f}}_{T,ML}(ML)$

**Table 2.** Simulation results for model (2).

True H	J	NSR=10%			NSR=20%			n=1000
		${\overset{ˇ}{f}}_{T}$	n=250	n=500	n=1000	n=250	n=500	

U Laplace								
$H_{1}$	2	NP	98[66,141]	68[47,93]	44[31,61]	117[78,166]	84[58,115]	56[41,78]
		${SP}_{\Phi }$	96[64,139]	66[47,90]	43[29,58]	115[78,164]	82[58,114]	55[39,77]
		${SP}_{H_{1}}$	96[65,139]	67[47,92]	43[29,59]	116[78,167]	83[58,114]	55[39,77]
		ML	655[609,703]	651[620,687]	648[624,672]	637[590,688]	633[600,670]	630[606,654]
	4	NP	84[57,121]	53[36,74]	33[23,45]	96[65,138]	60[44,84]	40[29,56]
		${SP}_{\Phi }$	92[63,140]	59[41,81]	38[28,51]	121[79,177]	78[55,108]	54[39,73]
		$SP_{H_{1}}$	93[63,138]	60[41,82]	38[28,52]	120[78,174]	78[55,109]	53[39,73]
		ML	650[613,691]	645[619,673]	644[622,661]	633[594,672]	626[601,655]	626[604,644]
$H_{2}$	2	NP	162[105,239]	112[73,158]	73[51,105]	181[123,274]	136[91,199]	94[65,134]
		${SP}_{H_{1}}$	231[161,314]	187[139,246]	164[123,205]	247[172,328]	199[143,261]	172[125,218]
		ML	812[765,865]	809[777,849]	808[784,835]	785[740,843]	783[750,823]	783[757,809]
	4	NP	116[73,173]	80[53,110]	51[35,73]	128[84,188]	93[62,130]	63[41,88]
		${SP}_{H_{1}}$	217[153,297]	182[135,237]	156[122,195]	246[170,334]	208[153,270]	177[136,219]
		ML	833[791,878]	826[792,855]	824[804,846]	806[764,849]	798[766,830]	797[776,819]
U normal								
$H_{1}$	2	NP	117[78,171]	74[52,106]	53[38,73]	145[98,212]	99[70,143]	73[51,101]
		$SP_{\Phi }$	114[75,162]	75[52,101]	51[38,70]	145[94,203]	99[68,136]	70[48,98]
		${SP}_{H_{1}}$	114[76,165]	76[52,102]	52[38,71]	146[94,204]	99[68,136]	69[48,97]
		ML	651[610,703]	658[623,690]	648[625,673]	634[592,689]	639[604,672]	631[608,656]
	4	NP	91[62,128]	61[42,84]	41[30,54]	111[74,161]	75[51,107]	53[36,74]
		$SP_{\Phi }$	109[78,159]	79[53,109]	56[40,78]	159[108,235]	118[77,177]	88[58,130]
		$SP_{H_{1}}$	108[77,161]	79[54,108]	56[40,77]	158[105,232]	113[77,171]	86[55,127]
		ML	650[615,692]	646[619,675]	642[625,662]	632[598,674]	627[601,658]	624[606,644]
$H_{2}$	2	NP	174[115,258]	125[86,179]	91[61,125]	208[134,314]	156[97,234]	115[74,170]
		${SP}_{H_{1}}$	235[170,316]	196[142,254]	171[130,218]	259[177,354]	203[140,282]	170[118,235]
		ML	812[770,872]	812[779,852]	811[784,835]	788[743,850]	788[752,826]	785[760,810]
	4	NP	131[85,191]	88[59,124]	63[46,86]	152[100,227]	107[70,156]	80[56,115]
		${SP}_{H_{1}}$	240[167,324]	205[147,271]	182[139,226]	283[192,389]	240[166,322]	200[147,272]
		ML	832[791,885]	826[798,857]	828[805,851]	806[764,856]	800[772,830]	801[780,824]

NOTE: The numbers show 10^5^× median [1st quartile, 3rd quartile] of 1000 ISEs for our nonparametric estimator ${\hat{f}}_{T}$ (NP), the semiparametric estimator, ${\hat{f}}_{T,SP_{H_{1}}}$ that assumes $H=H_{1}\left({SP}_{H_{1}}\right)$ and its version ${\hat{f}}_{T,{SP}_{\Phi }}$ that assumes $H=\Phi \left({SP}_{\Phi }\right)$, and the parametric estimator ${\hat{f}}_{T,ML}(ML)$.

**Table 3.** Simulation results for model (3).

True H	J	NSR=10%			NSR=20%			n=1000
		${\overset{ˇ}{f}}_{T}$	n=250	n=500	n=1000	n=250	n=500	

U Laplace								
$H_{1}$	2	NP	117[69,188]	72[44,112]	52[31,76]	114[68,194]	80[48,123]	58[36,86]
		${SP}_{\Phi }$	62[37,103]	37[23,57]	22[14,34]	66[41,112]	41[25,65]	26[17,39]
		${SP}_{H_{1}}$	63[37,108]	38[23,58]	22[14,35]	67[42,114]	42[26,67]	27[17,41]
		ML	18[7,37]	10[4,19]	4[2,9]	19[8,37]	10[4,19]	4[2,10]
	4	NP	67[41,108]	42[27,64]	29[18,42]	66[42,105]	45[29,65]	31[20,46]
		${SP}_{\Phi }$	58[32,105]	35[22,56]	21[13,31]	64[35,116]	40[24,62]	24[15,38]
		$SP_{H_{1}}$	60[34,114]	37[23,60]	21[13,34]	66[37,123]	41[25,69]	26[16,41]
		ML	11[5,23]	6[2,12]	3[1,6]	11[4,24]	6[2,12]	3[1,6]
$H_{2}$	2	NP	63[35,109]	42[25,66]	24[15,39]	65[37,113]	49[29,77]	30[18,47]
		${SP}_{H_{1}}$	126[78,188]	109[78,152]	94[72,122]	129[78,188]	109[77,153]	94[70,121]
		ML	67[49,93]	63[51,79]	57[49,68]	66[48,91]	62[50,78]	56[48,67]
	4	NP	44[25,72]	27[16,40]	16[10,25]	46[27,74]	28[17,44]	18[12,28]
		${SP}_{H_{1}}$	134[85,199]	117[80,164]	105[82,138]	138[86,210]	122[82,171]	110[85,142]
		ML	56[43,73]	52[42,63]	49[43,57]	56[42,72]	51[41,62]	48[42,56]
*U* normal								
$H_{1}$	2	NP	118[67,201]	75[46,118]	51[33,76]	127[73,231]	91[54,147]	64[40,102]
		$SP_{\Phi }$	65[37,106]	38[24,63]	24[16,36]	75[45,129]	48[29,79]	32[20,50]
		${SP}_{H_{1}}$	66[37,110]	38[24,65]	25[16,37]	76[45,131]	49[30,80]	32[20,51]
		ML	17[7,38]	9[4,18]	4[2,9]	18[7,39]	9[4,18]	4[2,9]
	4	NP	67[41,104]	42[27,63]	27[18,42]	70[42,116]	49[31,78]	37[23,56]
		${SP}_{\Phi }$	59[33,105]	35[22,58]	21[13,35]	76[39,138]	47[30,82]	33[19,56]
		$SP_{H_{1}}$	61[34,110]	36[22,61]	22[14,36]	78[41,140]	49[30,86]	34[20,58]
		ML	11[5,23]	6[2,11]	3[1,6]	11[5,23]	6[2,11]	3[1,6]
$H_{2}$	2	NP	66[36,103]	42[25,65]	28[17,42]	73[42,118]	51[32,81]	38[24,57]
		${SP}_{H_{1}}$	123[83,184]	111[80,157]	97[75,124]	128[81,190]	112[79,162]	98[73,128]
		ML	67[48,91]	63[49,78]	58[49,69]	67[47,90]	62[48,77]	58[48,69]
	4	NP	44[26,70]	26[16,43]	18[11,26]	50[29,80]	32[19,52]	23[14,35]
		$SP_{H_{1}}$	135[82,206]	118[82,165]	114[86,146]	158[93,234]	136[96,185]	131[98,162]
		ML	55[43,72]	52[43,63]	50[43,57]	55[42,72]	51[42,62]	49[42,56]

NOTE: The numbers show 10^5^× median [1st quartile, 3rd quartile] of 1000 ISEs for our nonparametric estimator ${\hat{f}}_{T}$ (NP), the semiparametric estimator, ${\hat{f}}_{T,SP_{H_{1}}}$ that assumes $H=H_{1}\left({SP}_{H_{1}}\right)$ and its version ${\hat{f}}_{T,{SP}_{\Phi }}$ that assumes $H=\Phi \left({SP}_{\Phi }\right)$), and the parametric estimator ${\hat{f}}_{T,ML}(ML)$.
