# Supplementary material for: Nonparametric Density Estimation of a Long-Term Trend from Repeated Semicontinuous Data
Source: J Am Stat Assoc. Author manuscript; Available in PMC 2026 Jun 3. (PMC13229538; doi:10.1080/01621459.2025.2555054)
Supplement: Supp 1 [file NIHMS2120148-supplement-Supp_1.zip › acc_form.html]

acc\_form.knit


This form documents the artifacts associated with the article (i.e.,
the data and code supporting the computational findings) and describes
how to reproduce the findings.

# Part 1: Data

- This paper does not involve analysis of
  external data (i.e., no data are used or the only data are generated by
  the authors via simulation in their code).

- I certify that the author(s) of
  the manuscript have legitimate access to and permission to use the data
  used in this manuscript.

## Abstract

This paper introduces and studies a nonparametric estimator for the
density of the long-term trend of a semicontinuous variable observed
repeatedly over time. Data from the Eating at America’s Table Study,
consisting of fruit and energy consumption collected from four repeated
24-hour dietary recalls on \(n=965\)
individuals, is used to illustrate the application of this estimator in
assessing the density of long-term total fruit consumption standardised
by energy intake. The four recalls were collected three months apart
(one per season) via telephone interviews conducted by trained
interviewers between September 1997 and August 1998.

## Availability

- Data **are**
  publicly available.
- Data **cannot be made**
  publicly available.

If the data are publicly available, see the *Publicly available
data* section. Otherwise, see the *Non-publicly available
data* section, below.

### Publicly available data

- Data are available online at:
- Data are available as part of
  the paper’s supplementary material.
- Data are publicly available by request,
  following the process described here:
- Data are or will be made available
  through some other mechanism, described here:

### Non-publicly available data

## Description

### File format(s)

- CSV or other plain text.
- Software-specific binary format (.Rda,
  Python pickle, etc.): pkcle
- Standardized binary format (e.g., netCDF,
  HDF5, etc.):
- Other (please specify):

### Data dictionary

- Provided by authors in the
  following file(s): “ETASDataDictionary.pdf”
- Data file(s) is(are) self-describing (e.g.,
  netCDF files)
- Available at the following URL:

### Additional Information (optional)

# Part 2: Code

## Abstract

We provide Matlab code for computing our estimator and the other
estimators considered in the numerical work of our paper, as well as one
R script called by our Matlab code for the monotonisation procedure. We
also provide the R codes for reproducing the tables and figures of the
paper.

The file “Readme.Rmd” describes how to use the code. An “Example.m”
file runs the code for one synthetic example.

## Description

### Code format(s)

- Script files
  - R
  - Python
  - Matlab
  - Other:
- Package
  - R
  - Python
  - MATLAB toolbox
  - Other:
- Reproducible report
  - R Markdown
  - Jupyter notebook
  - Other:
- Shell script
- Other (please specify):

### Supporting software requirements

The file “Readme.Rmd” describes how to reproduce the simulation
results and the real data analysis, and how to apply the methods to a
new data set.

#### Version of primary software used

Matlab 2024a and R-4.4.0

#### Libraries and dependencies used by the code

- Matlab function bwsjpiSM and its dependencies
  (bwosSM,bwosSM,lbinrSM and rootfSM) from Steve Marron’s Smoothing Matlab
  package available at: https://github.com/jsmarron/MarronMatlabSoftware/tree/master/Matlab9/Smoothing
- outerop Matlab function at https://au.mathworks.com/matlabcentral/fileexchange/8370-outer-operation?focused=5065519&tab=function
- R packages: ks 1.14.2, MASS 7.3.60.2, locfit 1.5.9.9, lpdensity
  2.4, DescTools 0.99.54

### Supporting system/hardware requirements (optional)

### Parallelization used

- No parallel code used
- Multi-core parallelization on a single
  machine/node
  - Number of cores used:
- Multi-machine/multi-node parallelization
  - Number of nodes and cores used:

### License

- MIT License (default)
- BSD
- GPL v3.0
- Creative Commons
- Other: (please specify)

### Additional information (optional)

# Part 3: Reproducibility workflow

## Scope

The provided workflow reproduces:

- Any numbers provided in text in
  the paper
- The computational method(s)
  presented in the paper (i.e., code is provided that implements the
  method(s))
- All tables and figures in the
  paper
- Selected tables and figures in the paper,
  as explained and justified below:

## Workflow

### Location

The workflow is available:

- As part of the paper’s
  supplementary material.
- In this Git repository:
- Other (please specify):

### Format(s)

- Single master code file
- Wrapper (shell) script(s)
- Self-contained R Markdown file,
  Jupyter notebook, or other literate programming approach
- Text file (e.g., a readme-style file) that
  documents workflow
- Makefile
- Other (more detail in *Instructions*
  below)

### Instructions

- Instructions to run the code and reproduce the simulations
  results and real data analysis are given in the file
  “Readme.Rmd”.
- Matlab functions for computing estimators are in the folder
  “Functions”.
- A worked out synthetic example is in the folder “Synthetic
  Example”.
- Additional files needed for the simulations are in the folder
  “Simulations”.
- Additional files required for the real data analysis are in the
  folder “RealData”.

### Expected run-time

Approximate time needed to reproduce the analyses on a standard
desktop machine:

- < 1 minute
- 1-10 minutes
- 10-60 minutes
- 1-8 hours
- > 8 hours
- Not feasible to run on a desktop machine,
  as described here:

### Additional information

- Matlab and R codes are documented.
- The run time above is to reproduce the results for all considered
  estimators and all settings considered in our simulation section,
  obtained by combining

  - 3 densities \(f\_X\)
  - 2 densities \(f\_U\)
  - 2 error variances
  - 4 functions \(H\)
  - 3 sample sizes,

  each time with 1000 samples.
