# Supplementary material for: Nonparametric Density Estimation of a Long-Term Trend from Repeated Semicontinuous Data
Source: J Am Stat Assoc. Author manuscript; Available in PMC 2026 Jun 3. (PMC13229538; doi:10.1080/01621459.2025.2555054)
Supplement: Supp 1 [file NIHMS2120148-supplement-Supp_1.zip › ExcessZeroHNPRevision3_Appendix.pdf]

Supplementary Material for "Nonparametric density estimation of a long-term trend from repeated semicontinuous data"

Félix Camirand Lemyre
Département de mathématiques, Université de Sherbrooke, Sherbrooke, Qc, Canada, felix.camirand.lemyre@usherbrooke.ca

Raymond J. Carroll
Department of Statistics, Texas A&M University, 3143 TAMU, College Station, TX 77843-3143, and School of Mathematical and Physical Sciences, University of Technology Sydney, Broadway NSW 2007, Australia, carroll@stat.tamu.edu

Aurore Delaigle
School of Mathematics and Statistics, University of Melbourne, Parkville, VIC, 3010, Australia, aurored@unimelb.edu.au

A Additional methodological details

A.1 Estimating ϕ_U and f_U

In the case where the distribution of the U_{ij}'s is unknown, f_U and ϕ_U need to be estimated in order to compute our estimators (see e.g. (3.19), (3.6) and (3.13)). This can be done from the replicated $\widetilde{W}_{ij}$'s, using the parametric or nonparametric estimators proposed by Camirand Lemyre et al. (2022), which are based on the fact that $f_{\widetilde{W}_{ij}-\widetilde{W}_{ij'}|W_{ij}>0,W_{ij'}>0} = f_U * f_U$. For the readers' convenience, here we summarize their nonparametric estimators of ϕ_U and f_U, with the details as in their Matlab codes.

Following their approach, to estimate $\phi_U(t)$ in (3.6) and (3.13), we take $\widetilde{\phi}_U(t) = \widehat{\phi}_U(t)\mathbb{I}\{\widehat{\phi}_U(t) \geqslant \tau_n\} + \rho(t)\mathbb{I}\{\widehat{\phi}_U(t) < \tau_n\}$, where

$$\widehat{\phi}_U(t) = \left| \frac{\sum_{j=1}^n \sum_{k<k'}^J \cos\{t(\widetilde{W}_{jk} - \widetilde{W}_{jk'})\}\mathbb{I}\,(W_{jk} > 0, W_{jk'} > 0)}{\sum_{j=1}^n \sum_{k<k'}^J \mathbb{I}(W_{jk} > 0, W_{jk'} > 0)} \right|^{1/2}, \tag{A.1}$$

$\tau_n > 0$ is a threshold computed as Section 5.2 of Camirand Lemyre et al. (2022) and

ρ is the characteristic function of a Laplace random variable with variance estimated by half the empirical variance of the $\widetilde{W}_{ij} - \widetilde{W}_{ij'}$'s for which $W_{ij} > 0$ and $W_{ij'} > 0$.

To estimate $\mathfrak{h}^{(-1)} * f_U(x)$ (see e.g. (3.19)), following their procedure, we first estimate $f_U(x)$ by $\max\{\widehat{f}_U(x), 0\}$, where $\widehat{f}_U(x) = (2\pi)^{-1} \int e^{-itx} \phi_{\widetilde{K}}(h_U t) \widehat{\phi}_U(t)\, dt$, with $\widehat{\phi}_U$ as at (A.1), $\widetilde{K}$ a kernel function and $h_U > 0$ a bandwidth, all chosen like those authors. Then, like them, we estimate $\mathfrak{h}^{(-1)} * f_U(x)$ by $\int_{\widehat{q}_{U*U,0.01}}^{\widehat{q}_{U*U,0.99}} \mathfrak{h}^{(-1)}(u) \widetilde{f}_U(x-u)\, du$, where $\widehat{q}_{U*U,\alpha}$ denotes the empirical α quantile of $f_U * f_U$ computed from the $\widetilde{W}_{ij} - \widetilde{W}_{ik}$'s for which $W_{ij} > 0$ and $W_{ik} > 0$, and $\widetilde{f}_U(x) = \widehat{f}_U(x) / \int_{\widehat{q}_{U*U,0.01}}^{\widehat{q}_{U*U,0.99}} \widehat{f}_U(u)\, du$ is a rescaled version of $\widehat{f}_U$ that integrates to 1 on $[\widehat{q}_{U*U,0.01}, \widehat{q}_{U*U,0.99}]$.

A.2 Details of monotonization procedure

In this section we give the details of our implementation of Dette et al.'s (2006) procedure to compute the monotonized version $\widehat{H}_M$ of our nonparametric estimator $\widehat{H}$ at (3.15) on the interval $[a_H, b_H]$.

A.2.1 Replacing $\widehat{H}$ by a version $\widetilde{H}$ truncated to $[0, 1]$

Before describing the monotonization procedure, unlike H which is a probability, the estimator $\widehat{H}$ can take values outside $[0, 1]$. We found that the monotonization procedure performed better when applied to a modified version $\widetilde{H}$ of $\widehat{H}$ that takes values in $[0, 1]$. A simple way to define $\widetilde{H}(x)$ would be to take $\widetilde{H}(x) = \min[\max\{\widehat{H}(x), 0\}, 1]$. However, if $\widehat{H}(x) > 1$ for too many values of x, then defining $\widetilde{H}(x)$ in that way implies replacing $\widehat{H}(x)$ by 1 for many x's. This can make Geenens' (2014) estimator $\widehat{f}_V^{\rm LL}(v)$ of $f_V(v)$ in (3.16) put too much weight around $v = 1$, which in turn can introduce bias into the monotonized estimator of H. Therefore, and noting that apart from erratic behaviour of $\widehat{H}(x)$ at isolated x's, it is for x large that $\widehat{H}(x)$ can be repeatedly larger than 1, we take $\widetilde{H}(x) = \min[\max\{\widehat{H}(x), 0\}, 1]$, unless $\widehat{H}(x) > 1$ for too many x's in $[q_{\widetilde{W},0.8}, q_{\widetilde{W},0.99}]$, where we take $\widetilde{H}(x) = s(x)$, with s a smooth strictly increasing function; see Algorithm 2 below for details of implementation. The choice

of s does not matter much as long as it is smooth, close to 1 and increasing; it has no asymptotic impact and its only purpose is to avoid introducing multiple 1's when replacing $\widehat{H}$ by $\widetilde{H}$. In our numerical implementation we took a cubic polynomial increasing on the relevant interval; see Algorithm 2.

Algorithm 2: Computing the version $\widetilde{H}$ of $\widehat{H}$ truncated to $[0,1]$.

1. Let x_m be the smallest value in $[q_{\widetilde{W},0.8}, q_{\widetilde{W},0.99}]$ such that $\widehat{H}(x) > 1$ for at least 4 different $x \geqslant x_m$ on the grid of points where we compute $\widehat{H}$.
2. If x_m does not exist, for $x \in [a_H, b_H]$, let $\widetilde{H}(x) = \min[\max\{\widehat{H}(x), 0\}, 1]$.
3. If x_m exists:
   3.1. for $x \in [x_m, b_H]$, let $\widetilde{H}(x) = s(x)$, with s a cubic polynomial increasing on $[x_m, b_H]$ so that $s'(x_m) = s'(b_H) = 0$, $s(x_m) = 0.99$ and $s(b_H) = 1$.
   3.2. for $x \in [a_H, x_m - \Delta_x]$, with $\Delta_x = b_H - x_m$, let $\widetilde{H}(x) = \min[\max\{\widehat{H}(x), 0\}, 1]$;
   3.3. to transition smoothly between $\widetilde{H}$ in 3.1 and 3.2, for $x \in [x_m - \Delta_x, x_m]$, let $\widetilde{H}(x) = \widehat{H}(x)s(x)$, with $s(x) = s(2x_m - x)$, the reflection of s around x_m.

A.2.2 Monotonizing $\widetilde{H}$

To make $\widetilde{H}$ strictly increasing on the interval $[H(a_H), H(b_H)]$, we use the estimator (3.18) applied to $\widetilde{H}$. Specifically, let $n^+ = \sum_{i=1}^{n}\sum_{j=1}^{J} \mathbb{I}(W_{ij} > 0)$ be the number of $\widetilde{W}_{ij}$'s used to compute $\widehat{H}$, and let $V_1 = \widetilde{H}(\upsilon_1), \ldots, V_{n^+} = \widetilde{H}(\upsilon_{n^+})$, with the υ_i's equispaced on $[a_H, b_H]$. We estimate the density f_V of $V = H(\Upsilon)$, with $\Upsilon \sim U[a_H, b_H]$, by the estimator $\check{f}_V^{\mathrm{LL}}$ described in Appendix A.2.3. For $t \in [m_{\widetilde{H}}, M_{\widetilde{H}}] \equiv [\min_{x\in[a_H,b_H]} \widetilde{H}(x), \max_{x\in[a_H,b_H]} \widetilde{H}(x)]$, we estimate $H^{(-1)}(t)$ by

$$\widehat{H^{(-1)}}(t) = a_H + (b_H - a_H)\int_{m_{\widetilde{H}}}^{t} \tilde{f}_V^{\mathrm{LL}}(v)\,\mathrm{d}v\,,$$

where $\tilde{f}_V^{\mathrm{LL}}$ is a rescaled version of $\check{f}_V^{\mathrm{LL}}$ computed using Geenens' (2024) R function `myintegral` adapted to our problem and provided with our R code. We obtain a strictly increasing estimator $\widehat{H}_M$ on $[a_H, b_H]$ by numerical inversion of $\widehat{H^{(-1)}}$. Algorithm 3 in Appendix A.2.4 summarizes all the steps for computing $\widehat{H}_M$ on $[a_H, b_H]$.

A.2.3 Estimating f_V

Our estimator f_V is based on Geenens' (2014) second order probit local likelihood estimator. Since the latter is designed for estimating densities f_Y supported on $[0,1]$, to apply it in our case, we need to rescale the V_i's defined in Appendix A.2.2 to the interval $[0,1]$. Noting that these take their values in $[m_{\tilde{H}}, M_{\tilde{H}}]$, for $i = 1, \ldots, n^+$, we define their rescaled version by

$$Y_i = (V_i - m_{\tilde{H}})/(M_{\tilde{H}} - m_{\tilde{H}}) .$$

To estimate f_Y, Geenens (2014) uses a well known approach where the Y_i's, which take their values in $[0,1]$, are transformed into variables S_i that take values on the whole real line. Since the density f_S of the S_i's is supported on $\mathbb{R}$, it does not suffer from boundary issues and can be estimated by a standard nonparametric density estimator. An estimator of f_Y is obtained by back-transforming that estimator of f_S.

Geenens' (2014) main contribution was to prove that, in order for this approach to perform well, the transformation and the estimator of f_S need to be carefully selected. He showed that good performance can be obtained by taking $S_i = \Phi^{-1}(Y_i) \sim f_S$, with Φ the standard normal cumulative distribution function, and by estimating f_S with a local likelihood estimator $\widehat{f}_S^{\mathrm{LL}}$ (Loader, 1996), which he computes with a standard normal kernel. Then for any $y \in (0,1)$, since $f_Y(y) = f_S\{\Phi^{-1}(u)\}/\phi\{\Phi^{-1}(y)\}$, with ϕ the standard normal density, he suggested to estimate $f_Y(y)$ by $\widehat{f}_Y^{\mathrm{LL}}(y) = \widehat{f}_S^{\mathrm{LL}}\{\Phi^{-1}(y)\}/\phi\{\Phi^{-1}(y)\}$.

Since $\Phi^{-1}(y) \to \pm\infty$ as $y \to 0$ or 1, there are numerical issues when computing $\widehat{f}_Y^{\mathrm{LL}}(y)$ (resp., S_i) for y (resp., Y_i) too close to 0 or 1. Therefore, in practice, Geenens (2014) computes $\widehat{f}_Y^{\mathrm{LL}}(y)$ only for y not too close to 0 or 1, and truncates the Y_i's that are too close to 0 or 1, effectively replacing f_Y and $\widehat{f}_Y^{\mathrm{LL}}$ in the above definitions by, respectively, the density f_Z of the truncated data Z_i and its estimator $\widehat{f}_Z^{\mathrm{LL}}$. To apply his truncated approach in our case, for $i = 1, \ldots, n^+$, we would let $S_i = \Phi^{-1}\big(Z_i\big) \sim f_S$ and estimate f_S by the local likelihood estimator $\widehat{f}_S^{\mathrm{LL}}$. For $z \in [0.01, 0.99]$, we would

define an estimator of $f_Z(z)$ by

$$\widehat{f}_Z^{\mathrm{LL}}(z) = \widehat{f}_S^{\mathrm{LL}}\{\Phi^{-1}(z)\}/\phi\{\Phi^{-1}(z)\}\,. \tag{A.2}$$

A problem with Geenens' (2014) hard truncation of the Y_i's described in the previous paragraph is that it introduces ties, which are known to cause artificial sharp peaks in nonparametric curve estimators (see e.g. Chang et al., 2018); furthermore, there may also be some ties among the Y_i's themselves. We found that we could improve performance in two ways: break the ties (if any) among the Y_i's by adding a small random perturbation, and replace the hard truncation of the Y_i's that are too close to 0 or 1 by adding to them small random perturbations, as follows. For $i = 1, \ldots, n^+$, we break existing ties by taking $Y_i' = Y_i + \epsilon_i \cdot 1\{\text{if } V_i \text{ is tied}\}$, where $\epsilon_i \sim \pm U[\delta, 2\delta]$, with $\delta = 10^{-7}$ and taking $\pm$ with equal probability. Then for the Y_i's too close to 0 or 1 (which we define as being less than δ away from 0 or 1), instead of taking the Z_i's to be a hard truncated version of the Y_i's, we take $Z_i = Y_i' + 2|\epsilon_i| \cdot 1\{Y_i' < \delta\} - 2|\epsilon_i| \cdot 1\{Y_i' > 1-\delta\} \sim f_Z$, with ϵ_i defined as above. Then following Geenens (2014), we take $S_i = \Phi^{-1}(Z_i) \sim f_S$, estimate f_S by the local likelihood estimator $\widehat{f}_S^{\mathrm{LL}}$, and for $z \in [0.01, 0.99]$, we compute the estimator $\widehat{f}_Z^{\mathrm{LL}}(z)$ as at (A.2).

Like Geenens (2014), to compute $\widehat{f}_S^{\mathrm{LL}}(s)$ we use the R function `locfit` with a standard normal kernel. This requires choosing a bandwidth h_S; Geenens (2014) suggests using a nearest neighbour bandwidth $h_{NN}(s;k) = |s - S_{(k),s}|$, where $S_{(k),s}$ denotes the kth closest S_i to s, with k chosen by cross-validation (CV), using a so-called WLSCV1 criterion. However, we found this too unreliable for estimating H. Since the standard KDE $\widehat{f}_{Z,\mathrm{KDE}}(z)$ of $f_Z(z)$ computed with the established plug-in bandwidth, works well for z away from 0 and 1, we took instead $k = k_{NN}$, where

$$k_{NN} = \arg\min_k \int_{q_{Z,0.1}}^{q_{Z,0.9}} |\widehat{f}_Z^{\mathrm{LL}}(z;k) - \widehat{f}_{Z,\mathrm{KDE}}(z)|\,\mathrm{d}z\,, \tag{A.3}$$

$q_{Z,\beta}$ denotes the empirical β quantile of the Z_i's and $\widehat{f}_Z^{\mathrm{LL}}(z;k)$ is defined like $\widehat{f}_Z^{\mathrm{LL}}(z)$ in (A.2), with $\widehat{f}_S^{\mathrm{LL}}$ computed using the bandwidth $h_{NN}(\cdot;k)$. We refer to this approach

as L2KDE in the paper. For $v \in [m_{\widetilde{H}} + 0.01(M_{\widetilde{H}} - m_{\widetilde{H}}), M_{\widetilde{H}} - 0.01(M_{\widetilde{H}} - m_{\widetilde{H}})]$, we deduce an estimator of $f_V(v)$ by taking

$$\widehat{f}_V^{\text{LL}}(v) = \widehat{f}_Z^{\text{LL}}\big\{(v - m_{\widetilde{H}})/(M_{\widetilde{H}} - m_{\widetilde{H}})\big\}/(M_{\widetilde{H}} - m_{\widetilde{H}})\,.$$

To obtain an estimator $\check{f}_V^{\text{LL}}$ of f_V on the entire interval $[m_{\widetilde{H}}, M_{\widetilde{H}}]$, we use constant extrapolation of $\widehat{f}_V^{\text{LL}}$.

A.2.4 Full algorithm for monotonized estimator of H

Algorithm 3 below summarizes all the steps for computing the monotonized estimator $\widehat{H}_M$ on the interval $[a_H, b_H]$.

Algorithm 3: Computing the monotonized estimator $\widehat{H}_M$ on the interval $[a_H, b_H]$.

1. On $[a_H, b_H] = [q_{\widetilde{W},0.001}, q_{\widetilde{W},0.995}]$, compute $\widehat{H}$ using Algorithm 1 in Section 5.3, and its version $\widetilde{H}$ truncated to $[0,1]$ using Algorithm 2 in Appendix A.2.
2. For $i = 1, \ldots, n^+$, let $V_i = \widetilde{H}(v_i)$, with the v_i's equispaced on $[a_H, b_H]$, $Y_i = (V_i - m_{\widetilde{H}})/(M_{\widetilde{H}} - m_{\widetilde{H}}) + \epsilon_i \cdot 1\{\text{if } V_i \text{ is tied}\}$ and $Z_i = Y_i + 2|\epsilon_i| \cdot 1\{Y_i < \delta\} - 2|\epsilon_i| \cdot 1\{Y_i > 1 - \delta\} \sim f_Z$, where $[m_{\widetilde{H}}, M_{\widetilde{H}}] \equiv [\min_{x \in [a_H, b_H]} \widetilde{H}(x), \max_{x \in [a_H, b_H]} \widetilde{H}(x)]$ and $\epsilon_i \sim \pm U[\delta, 2\delta]$, with $\delta = 10^{-7}$ and taking $\pm$ with equal probability.
3. Let Φ (resp., ϕ) denote the standard normal cumulative distribution function (resp., density), and for $i = 1, \ldots, n^+$, let $S_i = \Phi^{-1}\big(Z_i\big) \sim f_S$.
4. Estimate f_S by the local likelihood estimator $\widehat{f}_S^{\text{LL}}$ using the R `locfit` function and bandwidth $h_{NN}(\cdot; k_{NN})$ with k_{NN} at (A.3). For $z \in [0.01, 0.99]$, estimate $f_Z(z)$ by
$$\widehat{f}_Z^{\text{LL}}(z) = \widehat{f}_S^{\text{LL}}\{\Phi^{-1}(z)\}/\phi\{\Phi^{-1}(z)\}\,.$$
5. For $v \in [m_{\widetilde{H}} + 0.01(M_{\widetilde{H}} - m_{\widetilde{H}}), M_{\widetilde{H}} - 0.01(M_{\widetilde{H}} - m_{\widetilde{H}})]$, estimate $f_V(v)$ by
$$\widehat{f}_V^{\text{LL}}(v) = \widehat{f}_Z^{\text{LL}}\big\{(v - m_{\widetilde{H}})/(M_{\widetilde{H}} - m_{\widetilde{H}})\big\}/(M_{\widetilde{H}} - m_{\widetilde{H}})\,.$$
6. Obtain an estimator $\check{f}_V^{\text{LL}}$ of f_V on $[m_{\widetilde{H}}, M_{\widetilde{H}}]$ by extrapolation of $\widehat{f}_V^{\text{LL}}$.

7. For $t \in [m_{\widetilde{H}}, M_{\widetilde{H}}]$ estimate $H^{(-1)}(t)$ by $\widehat{H^{(-1)}}(t) = a_H + (b_H - a_H) \int_{m_{\widetilde{H}}}^{t} \widetilde{f}_V^{\mathrm{LL}}(v)\,\mathrm{d}v$, where $\widetilde{f}_V^{\mathrm{LL}}$ is a rescaled version of $\check{f}_V^{\mathrm{LL}}$ computed with Geenens' (2024) R function `myintegral` adapted to our problem.
8. Obtain the monotone estimator $\widehat{H}_M$ on $[a_H, b_H]$ by numerical inversion of $\widehat{H^{(-1)}}$.

A.3 Computing the estimator $\widehat{\{\mathcal{R}_S^{(-1)}\}'}$ of $\{\mathcal{R}^{(-1)}\}'$

In this section we give the details for estimating $\mathcal{R}^{(-1)}$ by $\widehat{\{\mathcal{R}_S^{(-1)}\}'}$ at (3.20). Recall from Section 3.3 that for any $t \in [\mathcal{R}(a_H), \mathcal{R}(b_H)]$, we can write

$$\mathcal{R}^{(-1)}(t) = a_H + (b_H - a_H) \int_{\mathcal{R}(a_H)}^{t} f_V(v)\,\mathrm{d}v\,,$$

where f_V is the density of $V = \mathcal{R}(\Upsilon)$, with $\Upsilon \sim U[a_H, b_H]$. Taking the derivative of this expression with respect to t, we deduce

$$\{\mathcal{R}^{(-1)}\}'(t) = (b_H - a_H) f_V(t)\,.$$

Therefore, to estimate $\{\mathcal{R}^{(-1)}\}'(t)$ it suffices to estimate the density f_V. Similar to the procedure described in Appendices A.2.2 and A.2.3, since f_V has a compact support $[\mathcal{R}(a_H), \mathcal{R}(b_H)]$ and is not continuous at the endpoints of this support, to estimate f_V we use the second order probit local likelihood estimator developed by Geenens (2014) for estimating densities supported on $[0, 1]$. The procedure follows the same steps as the one described in Appendix A.2.3, with only three differences. First, for $i = 1, \ldots, n^+$, the V_i's are defined by $V_i = \widehat{\mathcal{R}}_M(v_i)$, with the v_i's equispaced on $[a_H, b_H]$. Second, we compute the estimator $\check{f}_V^{\mathrm{LL}}$ of f_V on the interval $[a_T, b_T] = [\widehat{\mathcal{R}}_M(a_H), \widehat{\mathcal{R}}_M(b_H)]$. Third, to compute Geenens' (2014) estimator we use his WLSCV1 nearest neighbour bandwidth defined in Appendix A.2.3. We summarize all the steps for computing the resulting estimator $\widehat{\{\mathcal{R}_S^{(-1)}\}'}$ in Algorithm 4.

Algorithm 4: Computing $\{\widehat{\mathcal{R}_S^{(-1)}}\}'$ on the interval $[a_T, b_T] = [\widehat{\mathcal{R}}_M(a_H), \widehat{\mathcal{R}}_M(b_H)]$.

1. For $i = 1, \ldots, n^+$, let $V_i = \widehat{\mathcal{R}}_M(v_i)$, with the v_i's equispaced on $[a_H, b_H]$, $Y_i = (V_i - a_T)/(b_T - a_T) + \epsilon_i \cdot 1\{\text{if } V_i \text{ is tied}\}$ and $Z_i = Y_i + 2|\epsilon_i| \cdot 1\{Y_i < \delta\} - 2|\epsilon_i| \cdot 1\{Y_i > 1-\delta\} \sim f_Z$, where $\epsilon_i \sim \pm U[\delta, 2\delta]$, with $\delta = 10^{-7}$ and taking $\pm$ with equal probability.
2. For $i = 1, \ldots, n^+$, let $S_i = \Phi^{-1}(Z_i) \sim f_S$.
3. Estimate f_S by the local likelihood estimator $\widehat{f}_S^{\mathrm{LL}}$ using the R `locfit` function and Geenens' (2014) WLSCV1 nearest neighbour bandwidth. For $z \in [0.01, 0.99]$, estimate $f_Z(z)$ by $\widehat{f}_Z^{\mathrm{LL}}(z) = \widehat{f}_S^{\mathrm{LL}}\{\Phi^{-1}(z)\}/\phi\{\Phi^{-1}(z)\}$.
4. For $t \in [a_T + 0.01(b_T - a_T), b_T - 0.01(b_T - a_T)]$, estimate $f_V(t)$ by
$$\widehat{f}_V^{\mathrm{LL}}(t) = \widehat{f}_Z^{\mathrm{LL}}\big\{(t - a_T)/(b_T - a_T)\big\}/(b_T - a_T)\,.$$
5. Get an estimator $\check{f}_V^{\mathrm{LL}}$ of f_V on $[a_T, b_T]$ by constant extrapolation of $\widehat{f}_V^{\mathrm{LL}}$.
6. For $t \in [a_T, b_T]$, compute $\{\widehat{\mathcal{R}_S^{(-1)}}\}'(t) = (b_H - a_H)\check{f}_V^{\mathrm{LL}}(t)$.

A.4 Interval $[a_X, b_X]$ where we compute our nonparametric estimator of f_X

As indicated in Section 5.2, since the $\widetilde{W}_{ij}$'s are sparse in the tails of their distribution, we can only compute our nonparametric estimator $\widehat{f}_X$ of f_X on an interval $[a_X, b_X]$ away from those tails, where $\widehat{f}_X$ does not take implausibly large values. Here we show how we chose $[a_X, b_X]$ in our simulations, by essentially taking the largest range of x's where $\widehat{f}_X(x)$ was not implausibly large. As in the errors-in-variables regression case discussed by Delaigle (2014), implausibly large values of $\widehat{f}_X$ are usually so much larger than the overall nearby values of $\widehat{f}_X$ that they can be detected by a crude method. We proceeded as follows.

Let $x_1 < x_2 < \ldots < x_G$ denote the grid of points at which we computed $\widehat{f}_X$ and recall that $q_{\widetilde{W},\alpha}$ denotes the empirical α quantile of the observed $\widetilde{W}_{ij}$'s. Since implausibly large values of $\widehat{f}_X(x)$ arise in the tails of the distribution of the $\widetilde{W}_{ij}$'s, we only

tracked them for $x < q_{\widetilde{W},0.025}$ and $x > q_{\widetilde{W},0.975}$. Namely, we regarded $\widehat{f}_X$ as implausibly large at $x_j < q_{\widetilde{W},0.025}$ if $\widehat{f}_X(x_j) > \sum_{q_{\widetilde{W},0.025}<x_k<q_{\widetilde{W},0.1}} \widehat{f}_X(x_k)/2$, and implausibly large at $x_j > q_{\widetilde{W},0.975}$ if $\widehat{f}_X(x_j) > \sum_{q_{\widetilde{W},0.9}<x_k<q_{\widetilde{W},0.975}} \widehat{f}_X(x_k)/2$. Let x_m (resp., x_M) denote the largest $x_j < q_{\widetilde{W},0.025}$ (resp., smallest $x_j > q_{\widetilde{W},0.975}$) where $\widehat{f}_X$ was implausibly large, and note that when $\widehat{f}_X$ is implausible large at a point x_j, it usually has a sharp peak around x_j (rather than $\widehat{f}_X(x_j)$ being an isolated outlier). Let the peak of $\widehat{f}_X$ around x_m (resp., x_M) correspond to an interval $[x_m - a_1, x_m + a_2]$ (resp., $[x_M - b_1, x_M + b_2]$), as computed by our Matlab function `hatfxTdecUnknownUMultilocHP`. We took $a_X = x_m + a_2$ and $b_X = x_M - b_1$. Algorithm 5 summarizes the steps used to construct $[a_X, b_X]$.

**Algorithm 5:** Computing $[a_X, b_X]$.
1. Let $x_1 < x_2 < \ldots < x_G$ denote the grid of points at which we compute $\widehat{f}_X$.
2. If it exists, let x_m denote the largest $x_j < q_{\widetilde{W},0.025}$ such that $\widehat{f}_X(x_j) > \sum_{q_{\widetilde{W},0.025}<x_k<q_{\widetilde{W},0.1}} \widehat{f}_X(x_k)/2$, let the peak of $\widehat{f}_X$ around x_m correspond to an interval $[x_m - a_1, x_m + a_2]$, and take $a_X = x_m + a_2$. Otherwise let $a_X = x_1$
3. If it exists, let x_M denote the smallest $x_j > q_{\widetilde{W},0.975}$ such that $\widehat{f}_X(x_j) > \sum_{q_{\widetilde{W},0.9}<x_k<q_{\widetilde{W},0.975}} \widehat{f}_X(x_k)/2$, let the peak of $\widehat{f}_X$ around x_M correspond to an interval $[x_M - b_1, x_M + b_2]$ and take $b_X = x_M - b_1$. Otherwise let $b_X = x_G$.

A.5 Tail corrections for nonparametric estimator of f_T

Recall from (5.2) that for $t \in [a_T, b_T] = [\widehat{\mathcal{R}}_M(q_{\widetilde{W},0.001}), \widehat{\mathcal{R}}_M(q_{\widetilde{W},0.995})]$, our nonparametric estimator of $f_T(t)$ is defined by

$$\widehat{f}_T(t) = \begin{cases} \{\widehat{\mathcal{R}^{(-1)}}\}'(t)\widehat{f}_X\{\widehat{\mathcal{R}}_M^{(-1)}(t)\} & \text{if } \widehat{\mathcal{R}_M^{(-1)}}(t) \in [a_X, b_X] \\ \{\widehat{\mathcal{R}^{(-1)}}\}'(t)\widehat{f}_{X,\mathrm{SP}}\{\widehat{\mathcal{R}}_M^{(-1)}(t)\} & \text{otherwise,} \end{cases} \tag{A.4}$$

with $\{\widehat{\mathcal{R}^{(-1)}}\}'$ as at (5.1). In some cases, $\{\widehat{\mathcal{R}^{(-1)}}\}'$ can be too unreliable in the tails of f_T, which causes $\widehat{f}_T$ to be implausibly large. Using an approach similar to that in Appendix A.4, we search for such implausibly large values of $\widehat{f}_T(t)$ for $t < \widehat{\mathcal{R}}_M(q_{\widetilde{W},0.025})$

and $t > \widehat{\mathcal{R}}_M(q_{\widetilde{W},0.0.975})$. If and where such values are detected, we make tail corrections by replacing $\{\widehat{\mathcal{R}^{(-1)}}\}'$ in (A.4) by $\{\widehat{\mathcal{R}_S^{(-1)}}\}'$ at (3.20); let $\widehat{f}_{T,2}$ denote the resulting tail-corrected estimator of f_T. If $\widehat{f}_{T,2}$ also takes implausibly large values in the tails, and if $\widehat{f}_{T,3}$, obtained by replacing $\{\widehat{\mathcal{R}^{(-1)}}\}'$ in (A.4) by $\{\widehat{\mathcal{R}}_{\mathrm{SP}}^{(-1)}\}'$ from Section 5.4, has better tail behaviour (see Algorithm 6 below for details), then we take our tail-corrected estimator equal to $\widehat{f}_{T,3}$. We implement these tail-corrections through Algorithm 6, which is similar to Algorithm 5 in Appendix A.4. We used our Matlab function `RemoveAberrantValues.m` to compute the intervals around the peaks in steps 3 and 4 of the algorithm.

Algorithm 6: Replacing $\widehat{f}_T$ by a tail-corrected estimator.

1. Let $t_1 < t_2 < \ldots < t_G$ denote the grid of points at which we compute $\widehat{f}_T$.
2. For $\widetilde{f}_T = \widehat{f}_T$ (resp., $\widetilde{f}_T = \widehat{f}_{T,2}$), let t_m^* (resp., $t_{m,2}^*$) denote the largest $t_j < q_{\widetilde{W},0.025}$ such that $\widetilde{f}_T(t_j) > \sum_{\widehat{\mathcal{R}}_M(q_{\widetilde{W},0.025})<t_k<\widehat{\mathcal{R}}_M(q_{\widetilde{W},0.1})} \widetilde{f}_T(t_k)/2$, and let t_M^* (resp., $t_{M,2}^*$) denote the smallest $t_j > q_{\widetilde{W},0.975}$ such that $\widetilde{f}_T(t_j) > \sum_{\widehat{\mathcal{R}}_M(q_{\widetilde{W},0.9})<t_k<\widehat{\mathcal{R}}_M(q_{\widetilde{W},0.975})} \widetilde{f}_T(t_k)/2$.
3. If t_m^* does not exist, do not change $\widehat{f}_T$ in the left tail. Otherwise:
   - 3.1. Let the peak of $\widehat{f}_T$ around t_m^* correspond to an interval $[t_m^* - a_1, t_m^* + a_2]$.
   - 3.2. For $t < t_m^* + a_2$ replace $\widehat{f}_T(t)$ by $\widehat{f}_{T,2}(t)$.
   - 3.3. If $t_{m,2}^*$ exists, let the peak of $\widehat{f}_{T,2}$ around $t_{m,2}^*$ correspond to an interval $[t_{m,2}^* - a_{1,2}, t_{m,2}^* + a_{2,2}]$; if $\max_{t_1\leqslant t\leqslant t_{m,2}^*+a_{2,2}} \widehat{f}_{T,3}(t) \leqslant \max_{t_1\leqslant t\leqslant t_{m,2}^*+a_{2,2}} \widehat{f}_{T,2}(t)$, in step 3.2, replace $\widehat{f}_{T,2}(t)$ by $\widehat{f}_{T,3}(t)$ for $t < t_{m,2}^* + a_{2,2}$.
4. If t_M^* does not exist, do not change $\widehat{f}_T$ in the right tail. Otherwise:
   - 4.1. Let the peak of $\widehat{f}_T$ around t_M^* correspond to an interval $[t_M^* - b_1, t_M^* + b_2]$.
   - 4.2. For $t > t_M^* - b_1$ replace $\widehat{f}_T(t)$ by $\widehat{f}_{T,2}(t)$.
   - 4.3. If $t_{M,2}^*$ exists, let the peak of $\widehat{f}_{T,2}$ around $t_{M,2}^*$ correspond to an interval $[t_{M,2}^* - b_{1,2}, t_{M,2}^* + b_{2,2}]$; if $\max_{t_{M,2}^*-b_{1,2}\leqslant t\leqslant t_G} \widehat{f}_{T,3}(t) \leqslant \max_{t_{M,2}^*-b_{1,2}\leqslant t\leqslant t_G} \widehat{f}_{T,2}(t)$, in step 4.2, replace $\widehat{f}_{T,2}(t)$ by $\widehat{f}_{T,3}(t)$ for $t > t_{M,2}^* - b_{1,2}$.

A.6 Maximum likelihood estimator of f_T

We computed a parametric estimator of f_T by maximum likelihood, assuming that $H = H_1(x;\beta_0,\beta_1)$, $X \sim \phi_{\mu_X,\sigma_X}$ and $U \sim \phi_{0,\sigma_U}$, where $\phi_{\mu,\sigma}$ denotes the density of a $N(\mu,\sigma^2)$. To reduce the number of parameters to estimate by ML, as in Appendix A.1, we estimated σ_U^2 by $\widehat{\sigma}_U^2$ defined as half the empirical variance of the $\widetilde{W}_{ij} - \widetilde{W}_{ij'}$'s for which $W_{ij} > 0$ and $W_{ij'} > 0$. Then we estimated μ_X, σ_X^2, β_0 and β_1 by maximising the joint log likelihood of the W_{ij}'s (see e.g. Tooze et al., 2010). That is, we estimated these parameters by

$$(\widehat{\mu}_{X,\mathrm{ML}}, \widehat{\sigma}^2_{X,\mathrm{ML}}, \widehat{\beta}_{0,\mathrm{ML}}, \widehat{\beta}_{1,\mathrm{ML}}) = \mathrm{argmax}_{(\mu_X,\sigma_X^2,\beta_0,\beta_1)} \log L(\beta_0,\beta_1,\mu_X,\sigma_X^2)\,,$$

where

$$\log L(\beta_0,\beta_1,\mu_X,\sigma_X^2) = \sum_{i=1}^n \log \int \Big[\prod_{j=1}^J \big\{\phi_{0,\widehat{\sigma}_U}(\widetilde{W}_{ij}-x)\big\}^{1\{W_{ij}>0\}}\Big] H_1(x;\beta_0,\beta_1)^{n_{i,+}}$$
$$\times \{1 - H_1(x;\beta_0,\beta_1)\}^{J-n_{i,+}} \phi_{\mu_X,\sigma_X}(x)\,dx\,,$$

with $n_{i,+} = \sum_{j=1}^J 1\{W_{ij} > 0\}$. Finally, recalling that

$$f_T(t) = \{\mathcal{R}^{(-1)}(t)\}' f_X\{\mathcal{R}^{(-1)}(t)\} \qquad \text{with} \qquad \mathcal{R}(x) = H(x)\cdot(\mathfrak{h}^{(-1)} * f_U)(x)\,,$$

we computed the ML estimator of $f_T(t)$ by taking

$$\widehat{f}_{T,\mathrm{ML}}(t) = \{\widehat{\mathcal{R}}^{(-1)}_{\mathrm{ML}}(t)\}' \phi_{\widehat{\mu}_{X,\mathrm{ML}},\widehat{\sigma}_{X,\mathrm{ML}}}\{\widehat{\mathcal{R}}^{(-1)}_{\mathrm{ML}}(t)\}$$

where

$$\widehat{\mathcal{R}}_{\mathrm{ML}}(x) = H_1(x;\widehat{\beta}_{0,\mathrm{ML}},\widehat{\beta}_{1,\mathrm{ML}})\cdot(\mathfrak{h}^{(-1)} * \phi_{0,\widehat{\sigma}_U})(x)\,.$$

B Additional simulation results

Tables B.1 to B.3 report the first three quartiles of the ISE values in the nonmonotone cases from Section 6 where $H = H_3$ or H_4.

Table B.1: Simulation results for model (1). The numbers show $10^4\times$ median [1st quartile, 3rd quartile] of 1000 ISEs for our nonparametric estimator $\widehat{f}_T$ (NP), the semiparametric estimator $\widehat{f}_{T,\mathrm{SP}_{H_1}}$ that assumes $H = H_1$ (SP_{H_1}) and the parametric estimator $\widehat{f}_{T,\mathrm{ML}}$ (ML).

		NSR=10%			NSR=20%		
True H	J $\breve{f}_T$	$n=250$	$n=500$	$n=1000$	$n=250$	$n=500$	$n=1000$
H_3	2 NP	49[34,74]	34[23,50]	24[18,35]	56[38,80]	39[27,57]	29[21,41]
	SP_{H_1}	48[32,69]	33[23,47]	24[18,33]	51[35,75]	36[25,52]	27[19,36]
	ML	46[37,61]	41[35,49]	38[35,43]	46[36,61]	41[35,49]	37[34,43]
	4 NP	47[32,67]	31[21,43]	20[14,29]	49[34,73]	34[23,49]	24[17,35]
	SP_{H_1}	55[36,83]	40[27,57]	29[20,41]	62[40,94]	46[31,66]	35[24,48]
	ML	42[35,54]	38[34,44]	36[33,40]	42[34,53]	38[33,44]	36[33,39]

Table B.2: Simulation results for model (2). The numbers show $10^5\times$ median [1st quartile, 3rd quartile] of 1000 ISEs for our nonparametric estimator $\widehat{f}_T$ (NP), the semiparametric estimator $\widehat{f}_{T,\mathrm{SP}_{H_1}}$ that assumes $H = H_1$ (SP_{H_1}) and the parametric estimator $\widehat{f}_{T,\mathrm{ML}}$ (ML).

		NSR=10%			NSR=20%		
True H	J $\breve{f}_T$	$n=250$	$n=500$	$n=1000$	$n=250$	$n=500$	$n=1000$
H_3	2 NP	91[62,128]	54[40,78]	36[26,50]	109[72,154]	72[50,99]	48[34,68]
	SP_{H_1}	90[60,128]	56[40,79]	38[27,53]	108[69,153]	72[49,97]	48[34,67]
	ML	582[543,624]	577[554,608]	578[559,599]	566[525,610]	560[536,593]	562[542,584]
	4 NP	75[51,108]	48[33,66]	30[21,42]	88[58,126]	58[39,82]	38[26,53]
	SP_{H_1}	101[66,151]	70[47,98]	49[33,69]	138[95,198]	102[70,144]	78[55,106]
	ML	585[550,619]	577[552,604]	575[556,593]	570[533,604]	562[534,587]	559[541,577]
H_4	2 NP	196[130,288]	136[92,198]	93[63,134]	224[144,336]	165[108,237]	120[79,172]
	SP_{H_1}	280[203,375]	233[174,306]	205[160,257]	292[207,389]	244[175,320]	216[159,270]
	ML	866[814,927]	859[821,901]	861[834,890]	836[784,899]	832[793,875]	834[806,862]
	4 NP	137[85,195]	87[58,125]	62[43,87]	152[98,223]	104[70,149]	74[50,105]
	SP_{H_1}	257[185,345]	223[168,284]	207[163,248]	282[205,382]	248[185,317]	224[176,271]
	ML	882[838,933]	878[844,912]	875[851,900]	853[809,905]	849[815,880]	846[822,870]

C Identifiability results

In this section we show that the pair (f_X, H) is identifiable from the joint distribution of an observed pair (W_{i1}, W_{i2}) generated as in Section 2. We assume throughout that the conditions from Section 2 are satisfied.

Recall that, in this work, integrals with unspecified bounds (other than inverse Fourier transform integrals) denote Lebesgue integrals over $\mathbb{R}$; we refer to a function f as integrable if it is Lebesgue integrable over $\mathbb{R}$. Recall too that for any

Table B.3: Simulation results for model (3). The numbers show $10^5\times$ median [1st quartile, 3rd quartile] of 1000 ISEs for our nonparametric estimator $\widehat{f}_T$ (NP), the semiparametric estimator $\widehat{f}_{T,\mathrm{SP}_{H_1}}$ that assumes $H = H_1$ (SP_{H_1}) and the parametric estimator $\widehat{f}_{T,\mathrm{ML}}$ (ML).

True H	J	$\check{f}_T$	NSR=10% $n=250$	$n=500$	$n=1000$	NSR=20% $n=250$	$n=500$	$n=1000$
H_4	2	NP	72[42,110]	42[26,66]	30[18,46]	75[43,115]	50[30,74]	37[22,55]
		SP_{H_1}	129[85,184]	108[76,148]	102[76,131]	130[85,190]	111[78,151]	101[75,135]
		ML	69[53,95]	64[52,80]	62[53,73]	69[52,94]	63[50,80]	61[52,71]
	4	NP	48[30,78]	29[18,44]	18[12,27]	51[32,81]	32[20,48]	22[13,31]
		SP_{H_1}	140[92,203]	121[85,165]	114[84,146]	148[94,212]	127[89,173]	118[88,152]
		ML	60[46,78]	55[46,66]	53[47,61]	60[45,77]	54[45,64]	52[46,60]

integrable and continuous function f with a piecewise continuous derivative, we define the inverse Fourier transform $\int e^{-itx}\phi_f(t)\,\mathrm{d}t$ as the improper Riemann integral $\lim_{T\to\infty}\int_{-T}^{T} e^{-itx}\phi_f(t)\,\mathrm{d}t$. Our identification result uses the following form of the Fourier inversion theorem:

Theorem C.1 (Fourier inversion theorem; Theorem 7.5 in Vretblad, 2003)**.** *For any integrable and continuous function f that has a piecewise continuous derivative, we have $(2\pi)^{-1}\int e^{-itx}\phi_f(t)\mathrm{d}t = f(x)$ for all $x \in \mathbb{R}$.*

Below we establish identifiability of the pair (f_X, H) on the class

$$\mathcal{F} = \big\{(\widetilde{f}, \widetilde{H}) \text{ s.t. } \widetilde{f} \text{ is a continuous density, } \widetilde{H} : \mathbb{R} \to [0,1] \text{ is strictly increasing and continuous, and for } k = 1, 2,\ \widetilde{H}^k\widetilde{f} \text{ has a piecewise continuous derivative}\big\}.$$

When we want to highlight that a probability or a characteristic function is computed when $(f_X, H) \equiv (\widetilde{f}, \widetilde{H}) \in \mathcal{F}$, we add the superscript $\widetilde{f}, \widetilde{H}$ to its arguments. For example, for $(\widetilde{f}, \widetilde{H}) \in \mathcal{F}$, we let $F^{\widetilde{f},\widetilde{H}}_{W_1,W_2}$ denote the cumulative distribution function of a random vector (W_{i1}, W_{i2}) generated as in Section 2 when $(f_X, H) \equiv (\widetilde{f}, \widetilde{H})$, use $F^{\widetilde{f},\widetilde{H}}_{W_1} = F^{\widetilde{f},\widetilde{H}}_{W_2}$ for the marginals, and let $f^{\widetilde{f},\widetilde{H}}_{\widetilde{W}_1|W_1>0}$ and $f^{\widetilde{f},\widetilde{H}}_{\widetilde{W}_1|W_1>0,W_2>0}$ denote, respectively, the densities $f_{\widetilde{W}|W>0}$ and $f_{\widetilde{W}_{i1}|W_{i1}>0,W_{i2}>0}$ from Section 3, when $(f_X, H) \equiv (\widetilde{f}, \widetilde{H})$.

The next proposition establishes identifiability of (f_X, H) on $\mathcal{F}$ from the distribution $F^{f_X,H}_{W_1,W_2}$ of the observed data. Of course, since $H(x)$ is a conditional probability

where the conditioning argument is $X_i = x$, we cannot distinguish two candidates for H outside the support of f_X, whence the weaker form of identifiability for H.

Proposition C.2. *Under the conditions and model assumptions in Section 2, for any* $(\widetilde{f}_1, \widetilde{H}_1)$ *and* $(\widetilde{f}_2, \widetilde{H}_2)$ *in* $\mathcal{F}$, *if* $F^{\widetilde{f}_1,\widetilde{H}_1}_{W_1,W_2}(w_1, w_2) = F^{\widetilde{f}_2,\widetilde{H}_2}_{W_1,W_2}(w_1, w_2)$ *for all* $w_1 \geqslant 0, w_2 \geqslant 0$, *then* $\widetilde{f}_1(z) = \widetilde{f}_2(z)$ *for all* $z \in \mathbb{R}$, *and* $\widetilde{H}_1(z) = \widetilde{H}_2(z)$ *for all* $z \in \mathbb{R}$ *such that* $\widetilde{f}_1(z) > 0$.

Proof of Proposition C.2. If $(\widetilde{f}, \widetilde{H}) \in \mathcal{F}$, then for $k = 1, 2$, $\widetilde{H}^k\widetilde{f}$ is continuous; it is also integrable, since $\int |(\widetilde{H}^k\widetilde{f})(t)| \, \mathrm{d}t \leqslant 1$, using the fact that $|\widetilde{H}(t)| \leqslant 1$ and $\widetilde{f}$ is a density. Therefore, for $k = 1, 2$, the Fourier transform of $\widetilde{H}^k\widetilde{f}$ exists, and using Theorem C.1, we have, for all $x \in \mathbb{R}$,

$$(\widetilde{H}^k\widetilde{f})(x) = \frac{1}{2\pi} \int e^{-itx} \phi_{\widetilde{H}^k\widetilde{f}}(t) \, \mathrm{d}t \, .$$

Now under the conditions in Section 2, using (3.4) and (3.11), $f^{\widetilde{f},\widetilde{H}}_{\widetilde{W}_1|W_1>0}$ and $f^{\widetilde{f},\widetilde{H}}_{\widetilde{W}_1|W_1>0,W_2>0}$ satisfy $P^{\widetilde{f},\widetilde{H}}(W_1 > 0) f^{\widetilde{f},\widetilde{H}}_{\widetilde{W}_1|W_1>0} = (\widetilde{H}\widetilde{f}) * f_U$ and $P^{\widetilde{f},\widetilde{H}}(W_1 > 0, W_2 > 0) f^{\widetilde{f},\widetilde{H}}_{\widetilde{W}_1|W_1>0,W_2>0} = (\widetilde{H}^2\widetilde{f}) * f_U$. Moreover, recalling that the Fourier transform of f_U is equal to ϕ_U and using the results from the previous paragraph, we have that, for $k = 1, 2$, the Fourier transform of $(\widetilde{H}^k\widetilde{f}) * f_U$ exists and is equal to $\phi_{\widetilde{H}^k\widetilde{f}} \cdot \phi_U$. Letting $\phi^{\widetilde{f},\widetilde{H}}_{\widetilde{W}_+}$ and $\phi^{\widetilde{f},\widetilde{H}}_{\widetilde{W}_{++}}$ denote the Fourier transforms of $P^{\widetilde{f},\widetilde{H}}(W_1 > 0) f^{\widetilde{f},\widetilde{H}}_{\widetilde{W}_1|W_1>0}$ and $P^{\widetilde{f},\widetilde{H}}(W_1 > 0, W_2 > 0) f^{\widetilde{f},\widetilde{H}}_{\widetilde{W}_1|W_1>0,W_2>0}$, respectively, we deduce that $\phi_{\widetilde{H}\widetilde{f}} = \phi^{\widetilde{f},\widetilde{H}}_{\widetilde{W}_+}/\phi_U$ and $\phi_{\widetilde{H}^2\widetilde{f}} = \phi^{\widetilde{f},\widetilde{H}}_{\widetilde{W}_{++}}/\phi_U$. As in (3.5) and (3.12) we deduce that, for all $x \in \mathbb{R}$,

$$(\widetilde{H}\widetilde{f})(x) = \frac{1}{2\pi} \int e^{-itx} \phi^{\widetilde{f},\widetilde{H}}_{\widetilde{W}_+}(t)/\phi_U(t) \, \mathrm{d}t \text{ and } (\widetilde{H}^2\widetilde{f})(x) = \frac{1}{2\pi} \int e^{-itx} \phi^{\widetilde{f},\widetilde{H}}_{\widetilde{W}_{++}}(t)/\phi_U(t) \, \mathrm{d}t \, . \tag{C.1}$$

If $(\widetilde{f}_1, \widetilde{H}_1)$ and $(\widetilde{f}_2, \widetilde{H}_2)$ in $\mathcal{F}$ are such that $F^{\widetilde{f}_1,\widetilde{H}_1}_{W_1,W_2}(w_1, w_2) = F^{\widetilde{f}_2,\widetilde{H}_2}_{W_1,W_2}(w_1, w_2)$ for all $w_1 \geqslant 0, w_2 \geqslant 0$, then for all $z \in \mathbb{R}$, we have $F^{\widetilde{f}_1,\widetilde{H}_1}_{\widetilde{W}_1|W_1>0}(z) = F^{\widetilde{f}_1,\widetilde{H}_1}_{W_1|W_1>0}\{\mathfrak{h}^{(-1)}(z)\} = F^{\widetilde{f}_2,\widetilde{H}_2}_{W_1|W_1>0}\{\mathfrak{h}^{(-1)}(z)\} = F^{\widetilde{f}_2,\widetilde{H}_2}_{\widetilde{W}_1|W_1>0}(z)$ and $F^{\widetilde{f}_1,\widetilde{H}_1}_{\widetilde{W}_1|W_1>0,W_2>0}(z) = F^{\widetilde{f}_1,\widetilde{H}_1}_{W_1|W_1>0,W_2>0}\{\mathfrak{h}^{(-1)}(z)\} = F^{\widetilde{f}_2,\widetilde{H}_2}_{W_1|W_1>0,W_2>0}\{\mathfrak{h}^{(-1)}(z)\} = F^{\widetilde{f}_2,\widetilde{H}_2}_{\widetilde{W}_1|W_1>0,W_2>0}(z)$, since $\mathfrak{h}$ is strictly increasing.

Therefore, $f^{\widetilde{f}_1,\widetilde{H}_1}_{\widetilde{W}_1|W_1>0} = f^{\widetilde{f}_2,\widetilde{H}_2}_{\widetilde{W}_1|W_1>0}$ and $f^{\widetilde{f}_1,\widetilde{H}_1}_{\widetilde{W}_1|W_1>0,W_2>0} = f^{\widetilde{f}_2,\widetilde{H}_2}_{\widetilde{W}_1|W_1>0,W_2>0}$, which implies that $\phi^{\widetilde{f}_1,\widetilde{H}_1}_{\widetilde{W}_+} = \phi^{\widetilde{f}_2,\widetilde{H}_2}_{\widetilde{W}_+}$ and $\phi^{\widetilde{f}_1,\widetilde{H}_1}_{\widetilde{W}_{++}} = \phi^{\widetilde{f}_2,\widetilde{H}_2}_{\widetilde{W}_{++}}$; combined with (C.1), this implies that

$$\widetilde{H}_1\widetilde{f}_1 = \widetilde{H}_2\widetilde{f}_2 \quad \text{and} \quad \widetilde{H}_1^2\widetilde{f}_1 = \widetilde{H}_2^2\widetilde{f}_2 \,. \tag{C.2}$$

Now, from (C.2), and since the fact that $\widetilde{H}_1$ and $\widetilde{H}_2$ are strictly increasing implies that $\widetilde{H}_1(z) > 0$ and $\widetilde{H}_2(z) > 0$ for all $z \in \mathbb{R}$, the supports of $\widetilde{f}_1$ and $\widetilde{f}_2$ must coincide, so that for $z \in \mathbb{R}$, $\widetilde{f}_1(z) = 0 \iff \widetilde{f}_2(z) = 0$. Moreover, if (C.2) holds then for $z \in \mathbb{R}$ such that $\widetilde{f}_1(z) > 0$, we have $\widetilde{f}_1(z) = \widetilde{f}_2(z)$, since

$$\widetilde{f}_1(z) = \{\widetilde{H}_1(z)\widetilde{f}_1(z)\}^2/\{\widetilde{H}_1^2(z)\widetilde{f}_1(z)\} = \{\widetilde{H}_2(z)\widetilde{f}_2(z)\}^2/\{\widetilde{H}_2^2(z)\widetilde{f}_2(z)\} = \widetilde{f}_2(z)\,.$$

Therefore, $\widetilde{f}_1(z) = \widetilde{f}_2(z)$ for all $z \in \mathbb{R}$. Exploiting (C.2) again, we deduce that $\widetilde{H}_1(z) = \widetilde{H}_2(z)$ for all z such that $\widetilde{f}_1(z) > 0$, which concludes the proof. □

D Notation used in the Appendix

We use the following notations throughout the proofs. For any function f and $\ell \in \{0,1,2,3\}$, we write $f^{(\ell)}$ to denote its ℓth derivative, i.e. $f^{(0)} = f$, $f^{(1)} = f'$ and so on. We write $\|f\|_\infty$ to denote $\sup_{x\in\mathbb{R}} |f(x)|$. We also let $\mathcal{M} = H^2 f_X$ (as in Condition (A3)) and $\widehat{\mathcal{M}} = \widehat{H^2 f_X}$.

E Proofs in the case where f_U is known

E.1 Results related to monotonized estimators of H, $\mathcal{R}^{(-1)}$ and $\{\mathcal{R}^{(-1)}\}'$

The following proposition establishes a uniform strong consistency result for the monotonized version $\widehat{H}_M$ of $\widehat{H}$.

Proposition E.1. *Assume that Conditions (A1) to (A4) are satisfied. Fix* $[a,b] \subset \mathcal{I}$*, where* $\mathcal{I}$ *is an open interval on which* f_X *is bounded away from zero. Then, with* $m_{\widehat{H}} = \min_{x\in[a,b]} \widehat{H}(x)$ *and* $M_{\widehat{H}} = \max_{x\in[a,b]} \widehat{H}(x)$*, we have as* $n \to \infty$ *that*

$$\max\Big\{ \sup_{t\in[m_{\widehat{H}},M_{\widehat{H}}]} \big|\widehat{H}_M^{(-1)}(t) - H^{(-1)}(t)\big|, \sup_{x\in[a,b]} \big|\widehat{H}_M(x) - H(x)\big|\Big\}$$
$$= O_{a.s.}\{(\log n)^{1/2}(nh^{2\alpha+1})^{-1/2} + h^2 + \lambda\}\,,$$

and

$$\sup_{x\in[a,b]} \big|\widehat{H}_M'(x) - H'(x)\big| = O_{a.s.}\{(\log n)^{3/2}(nh^{2\alpha+3})^{-1/2} + \log(n)h^2\}\,.$$

Proof of Proposition E.1: To prove the proposition, we use Propositions G.2 and G.3 with $m \equiv H$ and $\widehat{m} \equiv \widehat{H}$. For this, we need to verify that H, $\widehat{H}$, $h_{S,H}$ and λ satisfy Conditions (C1) to (C5) stated at the beginning of Section G.

The fact that H satisfies Condition (C1) follows directly from the fact that we have assumed in Section 2 that is strictly increasing, and from assumption (A3), which implies that it is three times continuously differentiable wherever $f_X > 0$. Conditions (C4) and (C5) are immediate consequences of assumption (A4). Therefore, it only remains to show that Conditions (C2) and (C3) are also satisfied.

To achieve this, we show that there exist sequences κ_n, a_n and b_n such that $\max(\kappa_n, b_n) \to 0$, $a_n \to \infty$ and $\max(\kappa_n, b_n)a_n \to 0$ as $n \to \infty$, such that, as $n \to \infty$, (i) for $\ell \in \{0,1\}$, $\sup_{z\in[a,b]} |\widehat{H}^{(\ell)}(z) - H^{(\ell)}(z)| = O_{a.s.}(\kappa_n a_n^\ell + b_n)$; and (ii) for $\ell \in \{0,1,2,3\}$, $\sup_{z\in[a,b]} |\widehat{H}^{(\ell)}(z)| = O_{a.s.}(1 + a_n^\ell \kappa_n)$.

To prove (i) and (ii), it suffices to observe that, in view of Lemma F.7, these are satisfied with respectively $\kappa_n = (\log n)^{1/2}(nh^{2\alpha+1})^{-1/2}$, $b_n = h^2$ and $a_n = h^{-1}$.

Thus, we can apply Propositions G.2 and G.3, and conclude that as $n \to \infty$,

$$\max\Big\{ \sup_{t\in[m_{\widehat{H}},M_{\widehat{H}}]} \big|\widehat{H}_M^{(-1)}(t) - H^{(-1)}(t)\big|, \sup_{x\in[a,b]} \big|\widehat{H}_M(x) - H(x)\big|\Big\}$$
$$= O_{a.s.}\{(\log n)^{1/2}(nh^{2\alpha+1})^{-1/2} + h^2 + \lambda\} + |\Phi^{(-1)}(\lambda)|\zeta_n(h, h_{S,H})\,,$$

and

$$\sup_{x\in[a,b]} \left|\widehat{H}'_M(x) - H'(x)\right|$$
$$= O_{a.s.}\{(\log n)^{3/2}(nh^{2\alpha+3})^{-1/2} + \log(n)h^2 + \lambda\} + |\Phi^{(-1)}(\lambda)|\zeta_n(h, h_{S,H}),$$

where $\zeta_n(h, h_{S,H}) = O_{a.s.}[h_{S,H}^2 h^{-3} e^{\gamma_n^H}\{h^3 \log(n) + (\log n)^{1/2}(nh^{2\alpha+1})^{-1/2}\}]$ with $\gamma_n^H = \sqrt{2}\log^{1/2}[nh_{S,H}^3\{\sqrt{2\pi}\log^{1/2}(nh_{S,H}^3)\}^{-1}]$. From there, to conclude the proof of the proposition, it suffices to show that

$$|\Phi^{(-1)}(\lambda)|\zeta_n(h, h_{S,H}) = O_{a.s.}\{(\log n)^{1/2}(nh^{2\alpha+1})^{-1/2} + h^2\} \quad \text{as } n \to \infty. \tag{E.1}$$

Since Condition (A4) implies that as $n \to \infty$ we have

$$h^{-1}(\log n)^{1/2}(nh^{2\alpha+1})^{-1/2} = (\log n)^{1/2}(nh^{2\alpha+3})^{-1/2} = o(1),$$

then, (E.1) is proved if we show that

$$|\Phi^{(-1)}(\lambda)|h_{S,H}^2 h^{-2} e^{\gamma_n^H} \log(n) \to 0 \quad \text{as } n \to \infty. \tag{E.2}$$

To this end, first note (see e.g. Section 7.1 in Feller, 1968) that we have for any $x > 1$ that $\Phi(-x) \leqslant (2\pi)^{-1/2}\exp(-x^2/2)$. Since Condition (A4) implies that $nh_{S,H}^3\log^{-1/2}(nh_{S,H}) \to \infty$ as $n \to \infty$, which implies that $\gamma_n^H \to \infty$ as $n \to \infty$, we deduce that as $n \to \infty$, $\Phi(-\gamma_n^H) \leqslant (nh_S^3)^{-1}\log^{1/2}(nh_S) \leqslant \lambda$ (see Condition (A4)), and hence that $|\Phi^{(-1)}(\lambda)| \leqslant \gamma_n^H \leqslant \log n$ (see again Condition (A4)).

Also, since we have established in the last paragraph that $\gamma_n^H \to \infty$ as $n \to \infty$, then, for any small but fixed $\widetilde{\epsilon} > 0$, it holds for n sufficiently large that

$$e^{\gamma_n^H} = [\exp\{(\gamma_n^H)^2/2\}]^{2/\gamma_n^H} \leqslant \{nh_{S,H}^3\}^{\widetilde{\epsilon}} \leqslant n^{\widetilde{\epsilon}}, \tag{E.3}$$

where we used the fact that, as $n \to \infty$, Condition (A4) implies that $h_{S,H} \leqslant 1$ and $\{\sqrt{2\pi}\log^{1/2}(nh_{S,H}^3)\}^{-1} \leqslant 1$. Consequently, as under Condition (A4) $h_{S,H}h^{-1} = o(n^{-\epsilon})$ as $n \to \infty$ for some $\epsilon > 0$, taking $\widetilde{\epsilon} < 2\epsilon$ at (E.3) proves (E.2), and therefore that (E.1) holds. This concludes the proof of the proposition. □

Recall from (3.19) that we estimate $\mathcal{R}(x)$ by

$$\widehat{\mathcal{R}}_M(x) = \widehat{H}_M(x)(\mathfrak{h}^{(-1)} * f_U)(x)\,,$$

and that to estimate $\{\mathcal{R}^{(-1)}\}'(t)$ we take

$$\{\widehat{\mathcal{R}_S^{(-1)}}\}'(t) = (b-a)\,\widetilde{f}_V^{\mathrm{LL}}(t)\,,$$

with $\widetilde{f}_V^{\mathrm{LL}}$ as in Section 3.2, but replacing H and $\widehat{H}$ by $\mathcal{R}$ and $\widehat{\mathcal{R}}_M$, see (3.20).

Proposition E.2. *Under Conditions (A1) to (A5), for any $[a, b] \subset \mathcal{I}$, where $\mathcal{I}$ is an open interval on which Condition (A5) is satisfied, we have, as $n \to \infty$,*

$$\sup_{x\in[a,b]} \big|\widehat{\mathcal{R}}_M(x) - \mathcal{R}(x)\big| = O_{a.s.}\{(\log n)^{1/2}(nh^{2\alpha+1})^{-1/2} + h^2 + \lambda\}\ , \tag{E.4}$$

$$\sup_{x\in[a,b]} \big|\widehat{\mathcal{R}}'_M(x) - \mathcal{R}'(x)\big| = O_{a.s.}\{(\log n)^{3/2}(nh^{2\alpha+3})^{-1/2} + \log(n)h^2 + \lambda\}\,, \tag{E.5}$$

and

$$\sup_{t\in[\widehat{R}_M(a),\widehat{R}_M(b)]} |\widehat{\mathcal{R}}_M^{(-1)}(t) - \mathcal{R}^{(-1)}(t)| = O_{a.s.}\{(\log n)^{1/2}(nh^{2\alpha+1})^{-1/2} + h^2 + \lambda\}\,. \tag{E.6}$$

Proof of Proposition E.2: Under Conditions (A1) to (A4), it follows from Proposition E.1 that, as $n \to \infty$,

$$\max\Big\{\sup_{t\in[m_{\widehat{H}},M_{\widehat{H}}]} \big|\widehat{H}_M^{(-1)}(t) - H^{(-1)}(t)\big|, \sup_{x\in[a,b]} \big|\widehat{H}_M(x) - H(x)\big|\Big\}$$
$$= O_{a.s.}\{(\log n)^{1/2}(nh^{2\alpha+1})^{-1/2} + h^2 + \lambda\}\,,$$

and

$$\sup_{x\in[a,b]} \big|\widehat{H}'_M(x) - H'(x)\big| = O_{a.s.}\{(\log n)^{3/2}(nh^{2\alpha+3})^{-1/2} + \log(n)h^2\}\,.$$

Hence, since $\mathfrak{h}^{(-1)} * f_U(x)$ is continuous (see the assumptions on $\mathfrak{h}$ and f_U in Section 2), and as from the definition of $\mathcal{I}$ in the proposition's statement $\{\mathfrak{h}^{(-1)} * f_U(x)\}'$ is continuous on $\mathcal{I}$, which implies both $\mathfrak{h}^{(-1)} * f_U(x)$ and $\{\mathfrak{h}^{(-1)} * f_U(x)\}'$ are bounded

on $[a,b] \subset \mathcal{I}$, we obtain from $\widehat{\mathcal{R}}_M(x) - \mathcal{R}(x) = \{\widehat{H}_M(x) - H(x)\}\mathfrak{h}^{(-1)} * f_U(x)$ and $\widehat{\mathcal{R}}'_M(x) - \mathcal{R}'(x) = \{\widehat{H}'_M(x) - H'(x)\}\mathfrak{h}^{(-1)} * f_U(x) + \{\widehat{H}_M(x) - H(x)\}\{\mathfrak{h}^{(-1)} * f_U(x)\}'$, together with the last equations, that (E.4) and (E.5) hold as $n \to \infty$.

To show (E.6), it suffices to observe that since $\mathcal{I}$ is open and contains $[a,b]$, and since the right-hand side of (E.4) converges to 0 as $n \to \infty$ (see Condition (A4)), we have as $n \to \infty$ that $\mathcal{R}^{(-1)}\{\widehat{\mathcal{R}}_M(x)\} \in \mathcal{I}$ a.s.. Consequently, it holds as $n \to \infty$ that

$$\begin{aligned}\sup_{t\in[\widehat{R}_M(a),\widehat{R}_M(b)]} |\widehat{\mathcal{R}}_M^{(-1)}(t) - \mathcal{R}^{(-1)}(t)| &= \sup_{x\in[a,b]} |x - \mathcal{R}^{(-1)}\{\widehat{\mathcal{R}}_M(x)\}| \\ &= \sup_{x\in[a,b]} |\mathcal{R}^{(-1)}\{\mathcal{R}(x)\} - \mathcal{R}^{(-1)}\{\widehat{\mathcal{R}}_M(x)\}| \\ &= \sup_{x\in\mathcal{I}}\{\mathcal{R}'(x)\}^{-1} \times \sup_{x\in[a,b]} |\widehat{\mathcal{R}}_M(x) - \mathcal{R}(x)| \quad \text{a.s.,}\end{aligned}$$

where, to obtain the last line, we used the mean-value theorem.

(E.6) follows from (E.4) and the fact that, under Conditions (A5) and (A3), $\mathcal{R}'$ is bounded below by a constant on $\mathcal{I}$. This concludes the proof of the proposition. □

Proposition E.3. *Under Conditions (A1) to (A5), for any $[a,b] \subset \mathcal{I}$, where $\mathcal{I}$ is an open interval on which Condition (A5) is satisfied, we have, as $n \to \infty$,*

$$\begin{aligned}&\sup_{t\in[\widehat{\mathcal{R}}_M(a),\widehat{\mathcal{R}}_M(b)]} \left|\{\widehat{\mathcal{R}}_S^{(-1)}\}'(t) - \{\mathcal{R}^{(-1)}\}'(t)\right| \\ &\qquad = (\log n)\, O_{a.s.}\Big\{\lambda + (\log n)^{3/2}(nh^{2\alpha+3})^{-1/2} + (\log n)h^2\Big\}.\end{aligned}$$

Proof of Proposition E.3: To prove the result, we apply Proposition G.1 with $\widehat{m} = \widehat{\mathcal{R}}_M$ and $m = \mathcal{R}$. To do this, we need to verify that Conditions (C1), (C2) and (C4) stated at the beginning of Section G are satisfied for this choice for $\widehat{m}$ and m.

The fact that Conditions (C1) and (C4) are satisfied is a direct consequence of, respectively, the fact that H is strictly increasing, and Conditions (A3) to (A5). Therefore, it only remains to prove that for $\ell \in \{0,1\}$, there exist sequences $\kappa_n \to 0$, $a_n \to \infty$ and $b_n \to 0$ as $n \to \infty$ such that $\widehat{\mathcal{R}}_M^{(\ell)} - \mathcal{R}^{(\ell)} = O_{a.s}(\kappa_n a_n^\ell + b_n)$ on $[a,b]$.

As the conditions of Proposition E.2 are satisfied, and in view of (E.4) and (E.5) in the statement of the latter Proposition, it suffices to take $\kappa_n = (\log n)^{1/2}(nh^{2\alpha+1})^{-1/2}$, $a_n = h^{-1}\log(n)$ and $b_n = \log(n)h^2 + \lambda$.

Application of Proposition G.1 ensures that

$$\sup_{t\in[\widehat{\mathcal{R}}_M(a),\widehat{\mathcal{R}}_M(b)]} \left|\{\widehat{\mathcal{R}}_S^{(-1)}\}'(t) - \{\mathcal{R}^{(-1)}\}'(t)\right| = (\log n)\, O_{a.s.}\left\{\lambda + (\log n)^{3/2}(nh^{2\alpha+3})^{-1/2} + (\log n)h^2\right\},$$

where $\gamma_n^{\mathcal{R}} = \sqrt{2}\log^{1/2}[nh_{S,\mathcal{R}}^3\{\sqrt{2\pi}\log^{1/2}(nh_{S,\mathcal{R}}^3)\}^{-1}]$. From there, to conclude the proof of the proposition, it suffices to show that $e^{\gamma_n^{\mathcal{R}}}h_{S,\mathcal{R}}^2 = o(h^2)$.

To prove that $e^{\gamma_n^{\mathcal{R}}}h_{S,\mathcal{R}}^2 = o(h^2)$, from Condition (A4), there exists a constant $\epsilon > 0$ such that $h_{S,\mathcal{R}}^2 n^{2\epsilon} = o(h^2)$ as $n \to \infty$. Note too that since Condition (A4) imposes identical constraints on $h_{S,\mathcal{R}}$ and $h_{S,H}$, (E.3) in the proof of Proposition 4.1 is true if γ_n^H and $h_{S,H}$ there are replaced with $\gamma_n^{\mathcal{R}}$ and $h_{S,\mathcal{R}}$, which implies that we have, for n sufficiently large that $e^{\gamma_n^H} \leqslant n^{2\epsilon}$. This proves that $e^{\gamma_n^{\mathcal{R}}}h_{S,\mathcal{R}}^2 = o(h^2)$. This concludes the proof of the proposition. □

E.2 Result related to $\widehat{f}_X$

Lemma E.4. *Under Conditions (A1) to (A4), for any $[a, b] \subset \mathcal{I}$, where $\mathcal{I}$ is an open interval on which f_X is bounded away from zero, we have as $n \to \infty$ that*

$$\sup_{z\in[a,b]} \left|\widehat{f}_X(z) - f_X(z)\right| = O_{a.s.}\left\{(\log n)^{1/2}(nh^{2\alpha+1})^{-1/2} + h^2\right\}.$$

Proof of Lemma E.4: Since Conditions (A1) to (A4) are satisfied, Proposition F.5 and Lemma F.7 ensure that we have, as $n \to \infty$,

$$\sup_{x\in[a,b]} \max\{|\widehat{g}(x) - g(x)|, |\widehat{H}(x) - H(x)|\} = O_{a.s.}\{(\log n)^{1/2}(nh^{2(\alpha+l)+1})^{-1/2} + h^2\}. \tag{E.7}$$

Moreover, since, under Condition (A4), the right-hand side of (E.7) converges to 0 as $n \to \infty$, and since the fact that H is strictly increasing implies there exists $\epsilon_H > 0$

such that $H(z) > \epsilon_H$ for all $z \in [a,b]$ (recall that $[a,b] \in \mathcal{I}$), we deduce that, for n sufficiently large,

$$\inf_{z\in[a,b]} \widehat{H}(z) \geqslant \inf_{z\in[a,b]} H(z) - \sup_{z\in[a,b]} |\widehat{H}(z) - H(z)| > \epsilon_H/2 \quad \text{a.s.} \tag{E.8}$$

Now, recall that $\widehat{f}_X = \widehat{g}\widehat{H}^{-1}$ and $f_X = gH^{-1}$, so that $\widehat{f}_X - f_X = (\widehat{g} - g)\widehat{H}^{-1} + g(\widehat{H}^{-1} - H^{-1}) = \widehat{H}^{-1}\{(\widehat{g} - g) + f_X(H - \widehat{H})\}$. Using the above result, we have that, as $n \to \infty$, for all $x \in [a,b]$

$$|\widehat{f}_X(x) - f_X(x)| \leqslant |\widehat{H}^{-1}(x)|\big\{|\widehat{g}(x) - g(x)| + f_X(x)|H(x) - \widehat{H}(x)|\big\} \quad a.s.$$

This concludes the proof. □

E.3 Proof of Proposition 4.1

The result of Proposition 4.1 is a consequence of Proposition E.1. The latter implies that under Conditions (A1) to (A4), for any $[a,b] \subset \mathcal{I}$, with $\mathcal{I}$ an open interval on which f_X is bounded away from zero, we have, as $n \to \infty$,

$$\sup_{x\in[a,b]} \left|\widehat{H}_M(x) - H(x)\right| = O_{a.s.}\{(\log n)^{1/2}(nh^{2\alpha+1})^{-1/2} + h^2 + \lambda\}\,.$$

The proof follows from the fact that, under Condition (A4), $\lambda = O(h^2)$.

E.4 Proof of Theorem 4.2

Let $\mathcal{H} = [\widehat{\mathcal{R}}_M(a), \widehat{\mathcal{R}}_M(b)]$. Since $\widehat{\mathcal{R}}_M$ is strictly increasing on $[a,b]$ by construction, the map $x \mapsto \widehat{\mathcal{R}}_M(x)$ is one-to-one from $[a,b]$ to $[\widehat{\mathcal{R}}_M(a), \widehat{\mathcal{R}}_M(b)]$. Therefore for any $t \in \mathcal{H}$, $\widehat{\mathcal{R}}_M^{(-1)}(t) \in [a,b]$. Hence, since from the definition of $\widehat{f}_T$ at (3.21), and the expression of f_T at (3.1), we have

$$\widehat{f}_T(t) - f_T(t) = \{\widehat{\mathcal{R}_S^{(-1)}}\}'(t)\widehat{f}_X\{\widehat{\mathcal{R}}_M^{(-1)}(t)\} - \{\mathcal{R}^{(-1)}(t)\}'f_X\{\mathcal{R}^{(-1)}(t)\}\,,$$

we have

$$\sup_{t\in\mathcal{H}} |\widehat{f}_T(t) - f_T(t)| \leqslant J_1 \times J_2 + J_3 \times (J_4 + J_5) \tag{E.9}$$

where

$$
\begin{aligned}
J_1 &= \sup_{t\in\mathcal{H}} |\{\widehat{\mathcal{R}_S^{(-1)}}\}'(t) - [\mathcal{R}^{(-1)}]'(t)| \leqslant \sup_{t\in\mathcal{H}_H} |\{\widehat{\mathcal{R}_S^{(-1)}}\}'(t) - [\mathcal{R}^{(-1)}]'(t)| \\
J_2 &= \sup_{t\in\mathcal{H}} |\widehat{f}_X\{\widehat{\mathcal{R}}_M^{(-1)}(t)\}| \leqslant \sup_{x\in[a,b]} |\widehat{f}_X(x)| \\
J_3 &= \sup_{t\in\mathcal{H}} |\{\mathcal{R}^{(-1)}\}'(t)| \leqslant \sup_{t\in\mathcal{H}_H} |\{\mathcal{R}^{(-1)}\}'(t)| \\
J_4 &= \sup_{t\in\mathcal{H}} |\widehat{f}_X\{\widehat{\mathcal{R}}_M^{(-1)}(t)\} - f_X\{\widehat{\mathcal{R}}_M^{(-1)}(t)\}| \leqslant \sup_{x\in[a,b]} |\widehat{f}_X(x) - f_X(x)| \\
J_5 &= \sup_{t\in\mathcal{H}} |f_X\{\widehat{\mathcal{R}}_M^{(-1)}(t)\} - f_X\{\mathcal{R}^{(-1)}(t)\}|\,.
\end{aligned}
$$

In view of (E.9), the Theorem will be proved provided we show that

$$
J_j = O_{a.s.}\big[(\log n)^2\{(\log n)^{1/2}(nh^{2\alpha+3})^{-1/2} + h^2\}\big] \quad \text{for } j \in \{1,4,5\} \text{ as } n\to\infty, \tag{E.10}
$$

and that $J_j = O_{a.s.}(1)$ for $j \in \{2,3\}$ as $n \to \infty$.

Starting with J_1, we deduce from Proposition E.3 that, under Conditions (A1) to (A5), we have, as $n \to \infty$,

$$
\begin{aligned}
J_1 &\leqslant \sup_{t\in[\widehat{\mathcal{R}}_M(a),\widehat{\mathcal{R}}_M(b)]} \big|\{\widehat{\mathcal{R}}_S^{(-1)}\}'(t) - \{\mathcal{R}^{(-1)}\}'(t)\big| \\
&= (\log n)\, O_{a.s.}\Big\{\lambda + (\log n)^{3/2}(nh^{2\alpha+3})^{-1/2} + (\log n)h^2\Big\}\,,
\end{aligned}
$$

The proof of (E.10) in the case $j = 1$ follows from Condition (A4), which implies that $\lambda = O(h^2)$.

The proof of (E.10) in the case $j = 4$ follows directly from Lemma E.4.

To prove (E.10) in the case $j = 5$, under Conditions (A1) to (A5), we have from Proposition E.2 that, as $n \to \infty$,

$$
\sup_{t\in[\hat{R}_M(a),\hat{R}_M(b)]} |\widehat{\mathcal{R}}_M^{(-1)}(t) - \mathcal{R}^{(-1)}(t)| = O_{a.s.}\{(\log n)^{1/2}(nh^{2\alpha+1})^{-1/2} + h^2 + \lambda\}\,.
$$

Hence, from the inequality

$$
|f_X\{\widehat{\mathcal{R}}_M^{(-1)}(t)\} - f_X\{\mathcal{R}^{(-1)}(t)\}| \leqslant \|f_X\|_\infty |\widehat{\mathcal{R}}_M^{(-1)}(t) - \mathcal{R}^{(-1)}(t)|\,,
$$

we deduce that it holds, as $n \to \infty$, that

$$J_5 = O_{a.s.}\{(\log n)^{1/2}(nh^{2\alpha+1})^{-1/2} + h^2 + \lambda\}\,.$$

That (E.10) holds in the case $j = 5$ follows from the last equation and Condition (A4), ensuring that $\lambda = O(h^2)$.

From here, to conclude the proof of the Theorem, it remains to show that $J_j = O_{a.s.}(1)$ for $j \in \{2, 3\}$ as $n \to \infty$.

The case $j = 2$ follows directly from the fact that, since $|\widehat{f}_X| \leqslant |\widehat{f}_X - f_X| + f_X$, Lemma E.4 and Condition (A3) allow to deduce that as $n \to \infty$,

$$J_2 \leqslant O_{a.s.}\big\{(\log n)^{1/2}(nh^{2\alpha+1})^{-1/2} + h^2\big\} + \|f_X\|_\infty = O_{a.s.}(1)\,,$$

where, to obtain the last equality, we used the fact that, under Condition (A4), the first term on the right hand side of the inequality converges a.s. to 0 as $n \to \infty$.

For the case $j = 3$, using Proposition E.2, we know that for any $[a, b] \subset \mathcal{I}$, where $\mathcal{I}$ is an open interval on which Condition (A5) is satisfied, we have, as $n \to \infty$,

$$\sup_{x\in[a,b]} \big|\widehat{\mathcal{R}}_M(x) - \mathcal{R}(x)\big| = O_{a.s.}\{(\log n)^{1/2}(nh^{2\alpha+1})^{-1/2} + h^2 + \lambda\} = o_{a.s.}(1)\,,$$

where, to obtain the last equality, we used Condition (A4).

Since $\mathcal{R}$ is strictly increasing on $\mathcal{I}$ (recall the strictly increasing assumption over H and Condition (A5)), then for any sufficiently small $\epsilon > 0$ such that $[a-\epsilon, b+\epsilon] \subset \mathcal{I}$, we have $\mathcal{H} \subset [\mathcal{R}(a-\epsilon), \mathcal{R}(b+\epsilon)]$ a.s. as $n \to \infty$. Since $\{\mathcal{R}^{(-1)}\}'(t) = [\mathcal{R}'\{\mathcal{R}^{(-1)}(t)\}]^{-1}$, we deduce from Conditions (A3) and (A5) that as $n \to \infty$, we have, with probability one,

$$\begin{aligned} J_3 &\leqslant \sup_{x\in[a-\epsilon,b+\epsilon]} \{\mathcal{R}'(x)\}^{-1} \\ &= \sup_{x\in[a-\epsilon,b+\epsilon]} \{H'(x)\mathfrak{h}^{(-1)} * f_U(x) + H(x)[\mathfrak{h}^{(-1)} * f_U]'(x)\} = O(1)\,. \end{aligned}$$

This concludes the proof of the Theorem.

F Results on $\widehat{g}^{(\ell)}$, $\widehat{\mathcal{M}}^{(\ell)}$ and $\widehat{H}^{(\ell)}$

In this section, we derive almost sure bounds for the differences $\widehat{g}^{(\ell)} - g^{(\ell)}$, $\widehat{\mathcal{M}}^{(\ell)} - \mathcal{M}^{(\ell)}$ and $\widehat{H}^{(\ell)} - H^{(\ell)}$ used in Lemma F.7. The proofs of these three results rely on nearly identical technical arguments. Therefore, we provide the full proof for $\widehat{g}^{(\ell)}$ here and state the corresponding results for $\widehat{\mathcal{M}}^{(\ell)}$, which will be used in Lemma F.7.

The following lemma is a slight modification of Lemma 2.2 in Masry (1993).

Lemma F.1. *Under Conditions (A1), (A2), (A3) and (A4), there exists a constant $\eta > 0$ such that, for $\ell \in \{0, 1, 2, 3\}$ and n sufficiently large, we have*

$$|\widehat{g}^{(\ell)}(x) - \widehat{g}^{(\ell)}(y)| \leqslant \eta |x - y| h^{-\ell-1-\alpha}.$$

Proof of Lemma F.1: Using (3.6), the fact that ϕ_K is bounded and vanishes outside of $[-1, 1]$ (see Condition (A2)) and the inequality $|\widehat{\phi}_{\widetilde{W}_+}(t)| \leqslant 1$, we have

$$\begin{aligned} |\widehat{g}^{(\ell)}(x) - \widehat{g}^{(\ell)}(y)| &= \left|(2\pi)^{-1} \int_{-\infty}^{\infty} (-it)^\ell (e^{-itx} - e^{-ity}) \widehat{\phi}_{\widetilde{W}_+}(t) \phi_U^{-1}(t)\, \phi_K(ht)\, \mathrm{d}t \right| \\ &\leqslant (2\pi)^{-1} \int_{-1/h}^{1/h} |t|^\ell |e^{-itx} - e^{-ity}| |\widehat{\phi}_{\widetilde{W}_+}(t)| \phi_U^{-1}(t)\, \phi_K(ht)\, \mathrm{d}t \\ &\leqslant \pi^{-1} \|\phi_K\|_\infty |x - y| \int_0^{1/h} t^\ell \phi_U^{-1}(t)\, \mathrm{d}t\,. \end{aligned} \tag{F.1}$$

To bound the integral on the last line, we proceed as the proof of Theorem 2.1 of Fan (1991a) and deduce that under Condition (A1) there exist constants $M, C > 0$ such that for all $u \in \mathbb{R}$, $\phi_U(u) \geqslant C\mathbb{I}(|u| \leqslant M) + |u|^{-\alpha}(2c_U)^{-1}\mathbb{I}(|u| > M)$. Therefore,

$$\phi_U(u) \geqslant \min\{C, (2c_U)^{-1}|u|^{-\alpha}\} \geqslant \min\{C, (2c_U)^{-1}\} \min(1, |u|^{-\alpha}) \equiv \overline{C}^{-1} \min(1, |u|^{-\alpha})\,. \tag{F.2}$$

Hence, $\int_0^{1/h} t^\ell \phi_U^{-1}(t)\, \mathrm{d}t \leqslant \overline{C}\{1 + (\alpha + 1 + \ell)^{-1} h^{-\alpha-1-\ell})\}$.

The proof follows from the fact that, under Condition (A4), we have $h \to 0$ as $n \to \infty$. □

The following lemma uses arguments that are similar to those used in Fan (1991), Masry (1991) and Camirand Lemyre et al. (2022).

Lemma F.2. *Assume that Conditions (A2) to (A4) are satisfied. Then, for* $\ell \in \{0,1\}$*, as* $n \to \infty$*, we have* $\sup_{x\in\mathbb{R}} |\mathrm{E}\{\widehat{g}^{(\ell)}(x)\} - g^{(\ell)}(x)| = O(h^2)$*.*

Proof of Lemma F.2: Let $K_h(x) = h^{-1}K(x/h)$. From the definition of $\widehat{g}$ at (3.6), since $\mathrm{E}(\widehat{\phi}_{\widetilde{W}_+}) = p_{W_+}\mathrm{E}(e^{it\widetilde{W}_{jk}} \mid W_{jk} > 0) = \phi_{\widetilde{W}_+} = \phi_U\phi_g$, we have

$$\begin{aligned}\mathrm{E}\{\widehat{g}^{(\ell)}(x)\} &= \frac{1}{2\pi}\int_{-\infty}^{\infty}(-it)^{\ell}e^{-itx}\frac{\phi_{\widetilde{W}_+}(t)}{\phi_U(t)}\,\phi_K(ht)\,\mathrm{d}t = \frac{1}{2\pi}\int_{-\infty}^{\infty}(-it)^{\ell}e^{-itx}\phi_g(t)\,\phi_K(ht)\,\mathrm{d}t \\ &= \frac{1}{2\pi}\int_{-\infty}^{\infty}e^{-itx}\phi_{g^{(\ell)}}(t)\,\phi_K(ht)\,\mathrm{d}t \;=\; K_h * g^{(\ell)}(x)\,, \qquad \text{(F.3)}\end{aligned}$$

where we used the fact that $\phi_{g^{(\ell)}}(t) = (-it)^{\ell}\phi_g(t)$ and the Parseval identity.

From there, we proceed as in the proof of Proposition C.4 in Camirand Lemyre et al. (2022). Specifically, it follows from Condition (A2) that K satisfies (a) $\int K(u)\,\mathrm{d}u = 1$, (b) $\int uK(u)\,\mathrm{d}u = 0$, (c) $\int u^2K(u)\,\mathrm{d}u > 0$ and (d) $|K(u)| \leqslant \widetilde{\eta}(1+|u|^4)^{-1}$ for some constant $\widetilde{\eta} > 0$ (see the proof of Proposition C.4 in Camirand Lemyre et al., 2022, taking there $m+1=2$).

Then, using a Taylor expansion with the remainder in the integral form, we have, uniformly in $x \in \mathbb{R}$ and $\ell \in \{0,1\}$,

$$g^{(\ell)}(x+uh) - g^{(\ell)}(x) + g^{(\ell+1)}(x)(uh) = (uh)^2\int_0^1(1-\tau)g^{(\ell+2)}(x+\tau uh)\,\mathrm{d}\tau\,. \qquad \text{(F.4)}$$

Consequently, we deduce from (F.3) and the fact that $\|g^{(\ell+2)}\|_\infty < \infty$ (see Condition (A3)) that for $\ell \in \{0,1\}$,

$$\sup_{x\in\mathbb{R}}\left|\mathrm{E}\{\widehat{g}^{(\ell)}(x)\} - g^{(\ell)}(x)\right| \leqslant h^2\|g^{(\ell+2)}\|_\infty\int u^2|K(u)|\,\mathrm{d}u\,, \qquad \text{(F.5)}$$

where we used (a) and (b) above (F.4). Now, using (d) above (F.4), the left-hand side of (F.5) is $O(h^2)$, which proves the lemma. □

Lemma F.3. *Assume that Conditions (A1) to (A4) are satisfied. Then, for any* $\ell \in \{0,1,2,3\}$*, we have, as* $n \to \infty$*,*

$$nh^{2(\alpha+\ell)+1}\mathrm{Var}\{\widehat{g}^{(\ell)}(x)\} \to c_U^2 f_{\widetilde{W}_{jk}|W_{jk}>0}(x)J^{-1}\pi^{-1}\{\int_0^1 u^{2(\alpha+\ell)}\phi_K(u)\mathrm{d}u\}\,, \qquad \text{(F.6)}$$

where c_U *and* α *are as in Condition (A1) and* J *is the number of replicates.*

Proof of Lemma F.3: We deduce from (3.6) that

$$\widehat{g}^{(\ell)}(x) = \frac{-1}{nJh^{\ell+1}} \sum_{j=1}^{n} \sum_{k=1}^{J} K_U^{(\ell)}\Big(\frac{x - \widetilde{W}_{jk}}{h}\Big)\mathbb{I}(W_{jk} > 0)\,,$$

where we have used the notation

$$K_U(x) = \frac{1}{2\pi}\int_{-\infty}^{\infty} e^{-itx}\frac{\phi_K(t)}{\phi_U(t/h)}\,\mathrm{d}t \;= \frac{1}{2\pi}\int_{-\infty}^{\infty} \cos(tx)\frac{\phi_K(t)}{\phi_U(t/h)}\,\mathrm{d}t\,. \qquad \text{(F.7)}$$

We first show that

$$\lim_{n\to\infty} h^{2\alpha-1}\mathrm{E}\Big[\Big\{K_U^{(\ell)}\big(\frac{x - \widetilde{W}_{jk}}{h}\big)\Big\}^2 \mathbb{I}(W_{jk} > 0)\Big] = f_{\widetilde{W}_{jk}|W_{jk}>0}(x)p_{W_+}\pi^{-1}c_U^{-2}\int_0^1 w^{2(\ell+\alpha)}\phi_K^2(w)\,\mathrm{d}w\,. \qquad \text{(F.8)}$$

To achieve this, we compute that

$$\begin{aligned}\mathrm{E}\Big[\Big\{K_U^{(\ell)}\big(\frac{x - \widetilde{W}_{jk}}{h}\big)\Big\}^2 \mathbb{I}(W_{jk} > 0)\Big] &= \mathrm{E}\Big[\Big\{K_U^{(\ell)}\big(\frac{x - \widetilde{W}_{jk}}{h}\big)\Big\}^2 \mid W_{jk} > 0\Big]p_{W_+} \\ &= p_{W_+}\int \Big\{K_U^{(\ell)}\big(\frac{x - w}{h}\big)\Big\}^2 f_{\widetilde{W}_{jk}|W_{jk}>0}(w)\,\mathrm{d}w\,.\end{aligned}$$

From there, since combining (3.4) with the fact that f_U are g are continuous imply $f_{\widetilde{W}_{ji}|W_{jk}>0}$ is continuous, then, using also Condition (A2), we deduce that (F.8) holds by using Equation (3.3) in the proof of Theorem 2.1 in Fan (1991) .

Second, we show that for $k \neq k'$,

$$\lim_{n\to\infty} h^{2\alpha-1}\mathrm{E}\Big[K_U^{(\ell)}\big(\frac{x - \widetilde{W}_{jk}}{h}\big)K_U^{(\ell)}\big(\frac{x - \widetilde{W}_{jk'}}{h}\big)\mathbb{I}(W_{jk} > 0, W_{jk'} > 0)\Big] = 0\,. \qquad \text{(F.9)}$$

For this, we use the fact that the model assumptions in Section 2 implies $P(W_{ij} > 0, W_{ik} > 0|X_i = x) = H^2(x)$ to deduce that

$$\begin{aligned}&\mathrm{E}\Big[K_U^{(\ell)}\big(\frac{x - \widetilde{W}_{jk}}{h}\big)K_U^{(\ell)}\big(\frac{x - \widetilde{W}_{jk'}}{h}\big)\mathbb{I}(W_{jk} > 0, W_{jk'} > 0)\Big] \\ &= \mathrm{E}\Big\{K_U^{(\ell)}\big(\frac{x - \widetilde{W}_{jk}}{h}\big)K_U^{(\ell)}\big(\frac{x - \widetilde{W}_{jk'}}{h}\big) \mid W_{jk} > 0, W_{jk'} > 0\Big\}p_{W_+,W_+} \\ &= p_{W_+,W_+}\int \Big[\mathrm{E}\Big\{K_U^{(\ell)}\big(\frac{x - w - U_{jk}}{h}\big)\Big\}\Big]^2 H^2(w)f_X(w)\,\mathrm{d}w\,.\end{aligned}$$

Since

$$\begin{aligned}\mathrm{E}\Big\{K_U^{(\ell)}\big(\frac{x-w-U_{jk}}{h}\big)\Big\} &= \frac{1}{2\pi}\int_{-\infty}^{\infty}(-it)^{\ell}e^{-it\frac{x-w}{h}}\,\mathrm{E}\big(e^{-\frac{itU_{jk}}{h}}\big)\frac{\phi_K(t)}{\phi_U(t/h)}\,\mathrm{d}t\\ &= \frac{1}{2\pi}\int_{-\infty}^{\infty}(-it)^{\ell}e^{-it\frac{x-w}{h}}\phi_K(t)\,\mathrm{d}t \;=K^{(\ell)}\big(\frac{x-w}{h}\big)\,,\end{aligned}$$

we deduce from the fact that $K^{(\ell)}$ is bounded (see Conditions (A2)) and that $H \leqslant 1$ (it is a cumulative distribution function) that

$$\begin{aligned}&\mathrm{E}\Big[K_U^{(\ell)}\big(\frac{x-\widetilde{W}_{jk}}{h}\big)K_U^{(\ell)}\big(\frac{x-\widetilde{W}_{jk'}}{h}\big)\mathbb{I}(W_{jk}>0,W_{jk'}>0)\Big]\\ &\qquad\qquad = p_{W_+,W_+}\int\big\{K^{(\ell)}\big(\frac{x-w}{h}\big)\big\}^2H^2(w)f_X(w)\,\mathrm{d}w\\ &\qquad\qquad \lesssim p_{W_+,W_+}\int f_X(w)\,\mathrm{d}w \;=p_{W_+,W_+}\,.\end{aligned}$$

The latter inequality implies (F.9) since $\alpha > 1/2$ (see Condition (A1)) and $h \to 0$ as $n \to \infty$ (see Condition (A4)).

Third, note from (F.3) in the proof of Lemma F.2 that

$$h^{-\ell-1}\mathrm{E}\Big\{K_U^{(\ell)}\Big(\frac{x-\widetilde{W}_{jk}}{h}\Big)\mathbb{I}(W_{jk}>0)\Big\} = \mathrm{E}\{\widehat{g}^{(\ell)}(x)\} = K_h * g^{(\ell)}(x)\,.$$

Now Condition (A3) implies that $g^{(\ell)}$ is bounded. Moreover, under Condition (A2), we have from the proof of Lemma F.2 that $|K(u)| \lesssim (1+|u|^{m+3})^{-1}$, which implies that $\int|K_h| < \infty$. Therefore,

$$\big|h^{-\ell-1}\mathrm{E}\Big\{K_U^{(\ell)}\Big(\frac{x-\widetilde{W}_{jk}}{h}\Big)\mathbb{I}(W_{jk}>0)\Big\}\big| \leqslant \|g^{(\ell)}\|_\infty\int|K_h| \lesssim 1\,.$$

Since Condition (A4) implies that $h \to 0$ as $n \to \infty$, and since $\alpha > 1/2$ (see Condition (A1)), it follows that

$$\lim_{n\to\infty}h^{2\alpha-1}\mathrm{E}\Big\{K_U^{(\ell)}\Big(\frac{x-\widetilde{W}_{jk}}{h}\Big)\mathbb{I}(W_{jk}>0)\Big\}^2 = 0\,. \tag{F.10}$$

Combining (F.8)–(F.10), we deduce that for n sufficiently large and any $1 \leqslant k,k' \leqslant J$, we have

$$\lim_{n\to\infty}h^{2\alpha-1}\mathrm{Cov}\Big\{K_U^{(\ell)}\Big(\frac{x-\widetilde{W}_{jk}}{h}\Big)\mathbb{I}(W_{jk}>0),K_U^{(\ell)}\Big(\frac{x-\widetilde{W}_{jk'}}{h}\Big)\mathbb{I}(W_{jk'}>0)\Big\}$$

$$= \mathbb{I}(k = k') f_{\widetilde{W}_{jk}|W_{jk}>0}(x) p_{W_+} \pi^{-1} c_U^{-2} \int_0^1 w^{2(\ell+\alpha)} \phi_K^2(w)\, \mathrm{d}w \,.$$

The proof follows by combining this equation with the fact that $J < \infty$ (see Section 2), and the equality:

$$nJh^{2(\alpha+\ell)+1}\mathrm{Var}\big\{\widehat{g}^{(\ell)}(x)\big\} = h^{2\alpha-1}\mathrm{Var}\Big\{K_U^{(\ell)}\Big(\frac{x-\widetilde{W}_{jk}}{h}\Big)\mathbb{I}(W_{jk}>0)\Big\}$$
$$+ h^{2\alpha-1}(J-1)\mathrm{Cov}\Big\{K_U^{(\ell)}\Big(\frac{x-\widetilde{W}_{jk}}{h}\Big)\mathbb{I}(W_{jk}>0), K_U^{(\ell)}\Big(\frac{x-\widetilde{W}_{jk'}}{h}\Big)\mathbb{I}(W_{jk'}>0)\Big\}\,.$$

□

The following lemma is a slight modification of Theorem 2.2 in Masry (1993).

Lemma F.4. *Assume that conditions (A1) to (A4) are satisfied. Then, for any* $[a,b] \subset \mathbb{R}$ *and* $\ell \in \{0,1,2,3\}$*, we have, as* $n \to \infty$*,* $\sup_{x\in[a,b]} |\widehat{g}^{(\ell)}(x) - \mathrm{E}\{\widehat{g}^{(\ell)}(x)\}| = O_{a.s.}\{(\log n)^{1/2}(nh^{2(\alpha+\ell)+1})^{-1/2}\}$.

Proof of Lemma F.4: Let $\Upsilon_{n,\ell} = (\log n)^{1/2}(nh^{2(\alpha+\ell)+1})^{-1/2}$. Proceeding as in the proof of Theorem 2.2 in Masry (1993), let $r_{n,\ell} = h^{1+\ell+\alpha}\Upsilon_{n,\ell}$ and put $\mathcal{G}_{r_{n,\ell}} = \{a = x_1 < \ldots < x_{N_{r_{n,\ell}}-1} < b \leqslant x_{N_{r_{n,\ell}}} : x_{k+1} - x_k = r_{n,\ell}\}$, where $N_{r_{n,\ell}} = \lceil (b-a) r_{n,\ell}^{-1} \rceil$. We have

$$\sup_{x\in\mathcal{D}} |\widehat{g}^{(\ell)}(x) - \mathrm{E}\{\widehat{g}^{(\ell)}(x)\}| = \max_{1\leqslant k\leqslant N_{r_{n,\ell}}-1} \sup_{x\in[x_k,x_{k+1}]} |\widehat{g}^{(\ell)}(x) - \mathrm{E}\{\widehat{g}^{(\ell)}(x)\}|$$
$$\leqslant \max_{1\leqslant k\leqslant N_{r_{n,\ell}}-1} \sup_{x\in[x_k,x_{k+1}]} |\widehat{g}^{(\ell)}(x) - \widehat{g}^{(\ell)}(x_k)| + \max_{1\leqslant k\leqslant N_{r_{n,\ell}}} |\widehat{g}^{(\ell)}(x_k) - \mathrm{E}\{\widehat{g}^{(\ell)}(x_k)\}|$$
$$+ \max_{1\leqslant k\leqslant N_{r_{n,\ell}}-1} \sup_{x\in[x_k,x_{k+1}]} |\mathrm{E}\{\widehat{g}^{(\ell)}(x)\} - \mathrm{E}\{\widehat{g}^{(\ell)}(x_k)\}|\,. \tag{F.11}$$

Next we bound each term on the right hand side of the inequality in (F.11).

Using Lemma F.1 and the definition of the x_k's in $\mathcal{G}_{r_{n,\ell}}$ (see above (F.11)), under Conditions (A1), (A2) and (A4), we have, as $n \to \infty$,

$$\max_{1\leqslant k\leqslant N_{r_{n,\ell}}-1} \sup_{x\in[x_k,x_{k+1}]} |\widehat{g}^{(\ell)}(x) - \widehat{g}^{(\ell)}(x_k)| \leqslant O(h^{-1-\ell-\alpha}) \max_{1\leqslant k\leqslant N_{r_{n,\ell}}-1} \sup_{x\in[x_k,x_{k+1}]} |x - x_k|$$

$$= O(h^{-1-\ell-\alpha} r_{n,\ell}) = O(\Upsilon_{n,\ell}) \,. \tag{F.12}$$

Therefore, as $n \to \infty$, the term on the third line of (F.11) satisfies

$$\max_{1 \leqslant k \leqslant N_{r_{n,\ell}}-1} \sup_{x \in [x_k, x_{k+1}]} |\mathrm{E}\{\widehat{g}^{(\ell)}(x)\} - \mathrm{E}\{\widehat{g}^{(\ell)}(x_k)\}| = O(\Upsilon_{n,\ell}) \,. \tag{F.13}$$

From there, to conclude the proof of the lemma, we show that as $n \to \infty$,

$$\max_{1 \leqslant k \leqslant N_{r_{n,\ell}}} |\widehat{g}^{(\ell)}(x_k) - \mathrm{E}\{\widehat{g}^{(\ell)}(x_k)\}| = O_{a.s.}(\Upsilon_n^{(\ell)}(h)) \,. \tag{F.14}$$

To do this, let $\vartheta > 0$ denote a constant to be fixed later, and observe that

$$\mathbb{P}\Big\{ \max_{1 \leqslant k \leqslant N_{r_{n,\ell}}} |\widehat{g}^{(\ell)}(x_k) - \mathrm{E}\{\widehat{g}^{(\ell)}(x_k)\}| > \vartheta \Upsilon_{n,\ell} \Big\}$$
$$\leqslant N_{r_{n,\ell}} \max_{1 \leqslant k \leqslant N_{r_{n,\ell}}} \mathbb{P}\left\{ |\widehat{g}^{(\ell)}(x_k) - \mathrm{E}\{\widehat{g}^{(\ell)}(x_k)\}| > \vartheta \Upsilon_n^{(\ell)}(h) \right\} \,. \tag{F.15}$$

To bound the right hand side of (F.15), we use one of the Bernstein inequalities, which states that if $\xi_1, \ldots, \xi_n$ are i.i.d mean 0 random variables such that $|\xi_i| \leqslant c_\xi$ for some constant $c_\xi > 0$, then $\mathbb{P}(|\sum_{i=1}^n \xi_i| > \epsilon) \leqslant \exp[-\epsilon^2/\{2\sum_{i=1}^n \mathrm{E}(\xi_i^2) + 2c_\xi \epsilon/3\}]$. To apply that inequality to $\widehat{g}^{(\ell)}(x_k) - \mathrm{E}\widehat{g}^{(\ell)}(x_k)$, set

$$\xi_i = (nJh^{\ell+1})^{-1} \sum_{j=1}^J \Big[K_U^{(\ell)}\Big(\frac{\widetilde{W}_{ij} - x_k}{h}\Big) \mathbb{I}(W_{ij} > 0) - \mathrm{E}\Big\{ K_U^{(\ell)}\Big(\frac{\widetilde{W}_{ij} - x_k}{h}\Big) \mathbb{I}(W_{ij} > 0) \Big\} \Big] \,.$$

In this notation, we have $\widehat{g}^{(\ell)}(x_k) - \mathrm{E}\{\widehat{g}^{(\ell)}(x_k)\} = \sum_{i=1}^n \xi_i$. Thus, to use the Bernstein inequality to bound the right hand side of (F.15), we need to show that (i) $|\xi_i|$ is bounded and (ii) $\sum_{i=1}^n \mathrm{E}(\xi_i^2)$ is bounded.

To show (i), since Condition (A1) is satisfied, we deduce from (F.2) in the proof of Lemma F.1 that there exists a constant $\overline{C} > 0$ such that, for all $u \in \mathbb{R}$, $\phi_U(u) \geqslant \overline{C}^{-1} \min(1, |u|^{-\alpha})$. Consequently, since Condition (A2) implies that ϕ_K is bounded and vanishes outside of $[-1, 1]$, we deduce from the definition of K_U at (F.7) that

$$|K_U^{(\ell)}(x)| \leqslant \frac{\|\phi_K\|_\infty}{\pi} \int_0^1 |t|^\ell \phi_U^{-1}(t/h)\, \mathrm{d}t \leqslant \frac{\overline{C}\|\phi_K\|_\infty}{\pi} \Big(\int_0^h |t|^\ell \,\mathrm{d}t + h^{-\alpha} \int_h^1 |t|^{\ell-\alpha} \,\mathrm{d}t \Big) \,.$$

Since Condition (A1) implies that $\alpha > 1$, we deduce from the above equation that $\|K_U^{(\ell)}\|_\infty = O(h^{-\alpha})$. This implies that

$$\Big|J^{-1}\sum_{j=1}^{J} K_U^{(\ell)}\big(\frac{\widetilde{W}_{ij}-x_k}{h}\big)\mathbb{I}(W_{ij}>0)\Big| = O(h^{-\alpha})\,.$$

In view of the above inequality, and since Assumption (A4) implies that $nh \to \infty$, we conclude that there exists a constant $\widetilde{c}_\xi > 0$ such that for n large enough,

$$|\xi_i| \leqslant \widetilde{c}_\xi n^{-1}h^{-\alpha-\ell-1} \leqslant \widetilde{c}_\xi(nh^{2(\alpha+\ell)+1})^{-1/2}(n^{-1}h)^{1/2} < \widetilde{c}_\xi(nh^{2(\alpha+\ell)+1})^{-1/2}\,.$$

To show (ii), we deduce from Lemma F.3 that under conditions (A1) to (A4), $nh^{2(\alpha+\ell)+1}\mathrm{Var}\{\widehat{g}(x_k)\} = nh^{2(\alpha+\ell)+1}\sum_{i=1}^n \mathrm{E}(\xi_i^2) = O(1)$ as $n \to \infty$. Therefore, for n sufficiently large, $\sum_{i=1}^n \mathrm{E}(\xi_i^2) \leqslant Vn^{-1}h^{-2(\alpha+\ell)-1}$, where $V > 0$ is a constant.

Combining (i) and (ii) to the Bernstein inequality, we get, for n sufficiently large,

$$\mathbb{P}\left\{|\widehat{g}(x_k) - \mathrm{E}\{\widehat{g}(x_k)\}| > \vartheta\Upsilon_{n,\ell}\right\} \leqslant \exp\left\{-\frac{\vartheta^2\log(n)}{2V + 2\widetilde{c}_\xi/3}\right\} = n^{-\frac{\theta^2}{2V+2\tilde{c}_\xi/3}}\,.$$

Plugging the previous equation into (F.15) entails

$$\begin{aligned}\mathbb{P}\Big[\max_{1\leqslant k\leqslant N_{r_{n,\ell}}} |\widehat{g}(x_k) - \mathrm{E}\{\widehat{g}(x_k)\}| > \vartheta\Upsilon_{n,\ell}\Big] &\leqslant N_{r_{n,\ell}} n^{-\frac{\theta^2}{2V+2\tilde{c}_\xi/3}} \\ &\leqslant \big\{2(b-a)h^{-1-l-\alpha}(\Upsilon_{n,\ell})^{-1}\big\}n^{-\frac{\vartheta^2}{2V+2\tilde{c}_\xi/3}}\,.\end{aligned}$$

Since $1 \leqslant h^{-1} \leqslant n$ for n sufficiently large (see Condition (A4)), and since $\log n > 1$ when $n > 2$, then $h^{-1-\ell-\alpha}(\Upsilon_{n,\ell})^{-1} \leqslant n^{2+\ell+\alpha}$. Taking $\vartheta = (5+\ell+\alpha)(2V + 2\widetilde{c}_\xi/3)^{1/2}$ implies that the right hand side of the last inequality is bounded by n^{-2} for n sufficiently large. The proof of (F.14) follows from the Borel-Cantelli Theorem.

We conclude the proof of the lemma by combining the inequalities in (F.12), (F.13) and (F.14) . □

Proposition F.5. *Assume that conditions (A1) to (A4) are satisfied. Then, for* $\ell \in \{0,1\}$*, as* $n \to \infty$*, we have* $\sup_{x\in[a,b]}|\widehat{g}^{(\ell)}(x) - g(x)| = O_{a.s.}\{(\log n)^{1/2}(nh^{2(\alpha+\ell)+1})^{-1/2} + h^2\}$*. Moreover, for* $\ell \in \{0,1,2,3\}$*, as* $n \to \infty$*, we have* $\sup_{x\in[a,b]}|\widehat{g}^{(\ell)}(x)| = O_{a.s.}\{1 + (\log n)^{1/2}(nh^{2(\alpha+\ell)+1})^{-1/2}\}$*.*

Proof of Proposition F.5: Since $|\widehat{g}^{(\ell)}(x) - g(x)| \leqslant |\widehat{g}^{(\ell)}(x) - \mathrm{E}\{\widehat{g}^{(\ell)}(x)\}| + |\mathrm{E}\{\widehat{g}^{(\ell)}(x)\} - g^{(\ell)}(x)|$, the proof of the first claim in the statement of the proposition follows from Lemmas F.2 and F.4.

To prove the second claim, Lemma F.4 implies that

$$\sup_{x\in[a,b]} |\widehat{g}^{(\ell)}(x) - \mathrm{E}\{g^{(\ell)}(x)\}| = O_{a.s.}\{(\log n)^{1/2}(nh^{2(\alpha+\ell)+1})^{-1/2}\}\,.$$

Hence, to conclude from there, it suffices to show that $|\mathrm{E}\{g^{(\ell)}(x)\}| = O(1)$ as $n \to \infty$. To do this, since Condition (A3) implies that $g^{(\ell)}$ is bounded for $\ell \in \{0,1,2,3\}$, then we deduce from (F.3) in the proof of Lemma F.2 that $\mathrm{E}\{\widehat{g}^{(\ell)}(x)\} = K_h * g^{(\ell)}(x)$. Moreover, under Condition (A2), using the inequality $|K(u)| \leqslant \widetilde{\eta}(1+|u|^4)^{-1}$ derived in the proof of Lemma F.2 (see below (F.3) therein), with $\widetilde{\eta} > 0$ a constant, we have $\int |K_h| < \infty$. Therefore, $|\mathrm{E}\{\widehat{g}^{(\ell)}(x)\}| \leqslant \|g^{(\ell)}\|_\infty \int |K_h| = O(1)$, which concludes the proof. □

The proof of the following proposition can be derived similarly to that of Proposition F.5, using a sequence of Lemmas whose proofs are very similar to the proofs of Lemmas F.1 to F.4. Hence, its proof is omitted.

Proposition F.6. *Assume that Conditions (A1) to (A4) are satisfied. Then, for* $\ell \in \{0,1\}$*, as* $n \to \infty$ *we have* $\sup_{x\in[a,b]} |\widehat{\mathcal{M}}^{(\ell)}(x) - \mathcal{M}(x)| = O_{a.s.}\{(\log n)^{1/2}(nh^{2(\alpha+\ell)+1})^{-1/2} + h^2\}$*. Moreover, for* $\ell \in \{0,1,2,3\}$*, as* $n \to \infty$ *we have* $\sup_{x\in[a,b]} |\widehat{\mathcal{M}}^{(\ell)}(x)| = O_{a.s.}\{1 + (\log n)^{1/2}(nh^{2(\alpha+\ell)+1})^{-1/2}\}$*.*

Lemma F.7. *Under Conditions (A1) to (A4), for any compact set* $\mathcal{D}$ *such that* f_X *is bounded away from* 0 *on* $\mathcal{D}$*, we have for* $\ell \in \{0,1\}$ *that as* $n \to \infty$*,*

$$\sup_{x\in\mathcal{D}} |\widehat{H}^{(\ell)}(x) - H^{(\ell)}(x)| = O_{a.s.}\{(\log n)^{1/2}(nh^{2(\alpha+\ell)+1})^{-1/2} + h^2\} \quad a.s.\,. \tag{F.16}$$

Moreover, it also holds for $\ell \in \{0,1,2,3\}$ *that*

$$\sup_{x\in\mathcal{D}} |\widehat{H}^{(\ell)}(x)| = O_{a.s.}\{1 + (\log n)^{1/2}(nh^{2(\alpha+\ell)+1})^{-1/2}\} \quad as\ n \to \infty. \tag{F.17}$$

Proof of Lemma F.7: We first prove (F.16) in the case $\ell \in \{0,1\}$. To do this, we start by noting that under the assumptions of the lemma, we have from Propositions F.5 and F.6 that for $\ell \in \{0,1\}$, as $n \to \infty$,

$$\sup_{x\in\mathcal{D}} |\widehat{g}^{(\ell)}(x) - g^{(\ell)}(x)| = O_{a.s.}(\Upsilon_{n,\ell} + h^2) \qquad \sup_{x\in\mathcal{D}} |\widehat{\mathcal{M}}^{(\ell)}(x) - \mathcal{M}^{(\ell)}(x)| = O_{a.s.}(\Upsilon_{n,\ell} + h^2)\,, \tag{F.18}$$

where we use the notation $\Upsilon_{n,\ell} = (\log n)^{1/2}(nh^{2(\alpha+\ell)+1})^{-1/2}$.

Now, Condition (A4) implies that $(\log n)^{1/2}(nh^{2(\alpha+\ell)+1})^{-1/2} \to 0$ and $h \to 0$ as $n \to \infty$ for $\ell \in \{0,1\}$. Moreover, since the model assumptions in Section 2 imply that H is strictly increasing over $\mathbb{R}$, which ensures that $\inf_{x\in\mathcal{D}} H(x) > 0$, we have, from the definition of $\mathcal{D}$ in the statement of the lemma, that $\inf_{x\in\mathcal{D}} g(x) > 0$. Since Condition (A3) implies that $\mathcal{M}, g', \mathcal{M}'$ are bounded on $\mathcal{D}$, we deduce that there exists a constant $\vartheta > 0$ such that for $\ell \in \{0,1\}$ and n sufficiently large, we have, almost surely,

$$\inf_{x\in\mathcal{D}} \min\{\widehat{g}(x), g(x)\} > \vartheta^{-1} \quad \sup_{x\in\mathcal{D}} \max\left\{|\widehat{g}^{(\ell)}(x)|, |g^{(\ell)}(x)|, |\widehat{\mathcal{M}}^{(\ell)}(x)|, |\mathcal{M}^{(\ell)}(x)|\right\} \leqslant \vartheta\,. \tag{F.19}$$

The above inequalities implies that, for n sufficiently large, we have, with probability one,

$$\sup_{x\in\mathcal{D}} |\widehat{g}^{-1}(x) - g^{-1}(x)| \leqslant \vartheta^2 \sup_{x\in\mathcal{D}} |\widehat{g}(x) - g(x)| = O(\Upsilon_{n,0} + h^2)\,. \tag{F.20}$$

We are now ready to prove (F.16). In the case where $\ell = 0$, we have

$$\begin{aligned} |\widehat{H}(x) - H(x)| &= |\widehat{g}^{-1}(x)\widehat{\mathcal{M}}(x) - g^{-1}(x)\mathcal{M}(x)| \\ &\leqslant |\widehat{g}^{-1}(x)||\widehat{\mathcal{M}}(x) - \mathcal{M}(x)| + \mathcal{M}(x)|\widehat{g}^{-1}(x) - g^{-1}(x)|\,. \end{aligned} \tag{F.21}$$

Therefore, the proof of (F.16) in the case where $\ell = 0$ follows from (F.18) to (F.20).

To show (F.16) in the case where $\ell = 1$, first

$$\begin{aligned} \{\widehat{g}^{-1}(x) - g^{-1}(x)\}' &= g^{-2}(x)g'(x) - \widehat{g}^{-2}(x)\widehat{g}'(x) \\ &= g^{-2}(x)\{g'(x) - \widehat{g}'(x)\} \end{aligned}$$

$$+ \widehat{g}'(x)\widehat{g}^{-1}(x)g^{-1}(x)\{g^{-1}(x) + \widehat{g}^{-1}(x)\}\{\widehat{g}(x) - g(x)\}\,.$$

Thus, we deduce from (F.18) to (F.20) that, as $n \to \infty$,

$$\sup_{x\in\mathcal{D}} |\{\widehat{g}^{-1}(x) - g^{-1}(x)\}'| = O_{a.s.}(\Upsilon_{n,0} + \Upsilon_{n,1} + h^2) = O_{a.s.}(\Upsilon_{n,1} + h^2)\,, \qquad \text{(F.22)}$$

where, to obtain the last equality, we used the fact Condition (A4) implies that $h \to 0$ when $n \to \infty$, so that $\Upsilon_{n,0} = O(\Upsilon_{n,1})$.

From there, in view of the equality

$$\widehat{H}'(x) - H'(x) = [\widehat{g}^{-1}(x)\{\widehat{\mathcal{M}}(x) - \mathcal{M}(x)\}]' + [\mathcal{M}(x)\{\widehat{g}^{-1}(x) - g^{-1}(x)\}]'\,,$$

the proof of (F.16) in the case $\ell = 1$ follows from (F.18)–(F.20) and (F.22).

Next, we prove (F.17) in the case where $\ell \in \{0, 1, 2, 3\}$. Since $H \leqslant 1$, and as Condition (A3) ensures H' is bounded on $\mathcal{D}$, the fact that (F.17) holds for $\ell \in \{0, 1\}$ can readily be deduced from the inequality $|\widehat{H}^{(\ell)}| \leqslant |H^{(\ell)}| + |\widehat{H}^{(\ell)} - H^{(\ell)}|$ together with (F.16). Hence, it remains to prove (F.17) for $\ell \in \{2, 3\}$.

To do this, using Propositions F.5 and F.6, we write, for $\ell \in \{0, 1, 2, 3\}$ and as $n \to \infty$,

$$\sup_{x\in\mathcal{D}} |\widehat{g}^{(\ell)}(x)| = O_{a.s.}(1 + \Upsilon_{n,\ell}) \quad \sup_{x\in\mathcal{D}} |\widehat{\mathcal{M}}^{(\ell)}(x) - \mathcal{M}^{(\ell)}(x)| = O_{a.s.}(1 + \Upsilon_{n,\ell})\,. \quad \text{(F.23)}$$

Since

$$\widehat{H}''(x) = \widehat{g}^{-3}(x)\big[\widehat{\mathcal{M}}''(x)\widehat{g}^2(x) + \{\mathcal{M}(x)\widehat{g}''(x) - 2\widehat{\mathcal{M}}'(x)\widehat{g}'(x)\}\widehat{g}(x) - 2\widehat{\mathcal{M}}(x)\{\widehat{g}'(x)\}^2\big]$$

and

$$\begin{aligned}
&\widehat{H}'''(x) \\
&= -3\widehat{g}^{-4}(x)g'(x)\big[\widehat{\mathcal{M}}''(x)\widehat{g}^2(x) + \{\mathcal{M}(x)\widehat{g}''(x) - 2\widehat{\mathcal{M}}'(x)\widehat{g}'(x)\}\widehat{g}(x) - 2\widehat{\mathcal{M}}(x)\{\widehat{g}'(x)\}^2\big] \\
&\quad + \widehat{g}^{-3}(x)\big[\widehat{\mathcal{M}}'''(x)\widehat{g}^2(x) + 2\widehat{\mathcal{M}}''(x)\widehat{g}(x)\widehat{g}'(x) + \{\mathcal{M}(x)\widehat{g}''(x) - 2\widehat{\mathcal{M}}'(x)\widehat{g}'(x)\}\widehat{g}'(x) \\
&\qquad + \{\mathcal{M}'(x)\widehat{g}''(x) + \mathcal{M}(x)\widehat{g}'''(x) - 2\widehat{\mathcal{M}}''(x)\widehat{g}'(x) - 2\widehat{\mathcal{M}}'(x)\widehat{g}''(x)\}\widehat{g}(x) \\
&\qquad - 2\widehat{\mathcal{M}}'(x)\{\widehat{g}'(x)\}^2 - 4\widehat{\mathcal{M}}(x)\{\widehat{g}'(x)\}\widehat{g}''(x)\big]\,,
\end{aligned}$$

the proof follows from (F.19) and (F.23). □

G Results related to the estimation of inverses and their derivative

In this section, we prove three general results which are applied to derive strong consistency rates for estimators of H, $\mathcal{R}^{(-1)}$ and $\{\mathcal{R}^{(-1)}\}'$ for known/unknown f_U. To study properties of the monotonization procedure described in Section 3.2, as well as the estimator of $\{\mathcal{R}^{(-1)}\}'$ at (3.20), let $m : \mathbb{R} \to \mathbb{R}$ denote a strictly increasing function and let $\widehat{m}$ denote an initial estimator of m, to be monotonized over an interval $[a, b] \subset \mathbb{R}$, and constructed from a sample of size $n^+ = \sum_{i=1}^n \sum_{j=1}^J I(W_i > 0)$. Reflecting the procedure described in Section 3.2, let $U_1 = a, U_2, \ldots, U_{n^+-1}, U_{n^+} = b$ equispaced on $[a, b]$, let $t_{\mathrm{L},\widehat{m}} = \min_{x\in[a,b]} \widehat{m}(x)$ and $t_{\mathrm{U},\widehat{m}} = \max_{x\in[a,b]} \widehat{m}(x)$, and define the variables $V_i^{\widehat{m}} = \{\widehat{m}(U_i) - t_{\mathrm{L},\widehat{m}})\}/(t_{\mathrm{U},\widehat{m}} - t_{\mathrm{L},\widehat{m}}) \in [0, 1]$. Then, estimate $m^{(-1)}(t)$ by

$$\widehat{m^{(-1)}}(t) = a + (b - a) \int_{t_{\mathrm{L},\widehat{m}}}^{t} \tilde{f}_V^{\mathrm{LL}}(z)\, \mathrm{d}z\,, \tag{G.1}$$

where we take $\tilde{f}_V^{\mathrm{LL}}(z) = \check{f}_V^{\mathrm{LL}}(z)/\int_{t_{\mathrm{L},\widehat{m}}}^{t_{\mathrm{U},\widehat{m}}} \check{f}_V^{\mathrm{LL}}(z)\mathrm{d}z$, with

$$\begin{aligned}\check{f}_V^{\mathrm{LL}}(z) =& \mathbb{I}(z \in [t_{\mathrm{L},\widehat{m}}, t_{\mathrm{L},\widehat{m}} + \Delta])c_1(z)\\ &+ \mathbb{I}(z \in [t_{\mathrm{L},\widehat{m}} + \Delta, t_{\mathrm{U},\widehat{m}} - \Delta])\widehat{f}_V^{\mathrm{LL}}(z)\\ &+ \mathbb{I}(z \in [t_{\mathrm{U},\widehat{m}} - \Delta, t_{\mathrm{U},\widehat{m}}])c_2(z)\,.\end{aligned}$$

Here, c_1 and c_2 are (bounded) constant or linear extrapolation functions calculated from the values of $\widehat{f}_V^{\mathrm{LL}}$ near the boundaries of $[t_{\mathrm{L},\widehat{m}} + \Delta, t_{\mathrm{U},\widehat{m}} - \Delta]$, and $\Delta = \lambda(t_{\mathrm{U},\widehat{m}} - t_{\mathrm{L},\widehat{m}})$ with $\lambda \in (0, 1/2)$. For simplicity here we consider constant extrapolation function and we set $c_1(z) = \widehat{f}_V^{\mathrm{LL}}(t_{\mathrm{L},\widehat{m}} + \lambda\Delta^{\widehat{m}})$ and $c_2(z) = \widehat{f}_V^{\mathrm{LL}}(t_{\mathrm{U},\widehat{m}} - \lambda\Delta^{\widehat{m}})$, where we used the notation $\Delta^{\widehat{m}} = t_{\mathrm{U},\widehat{m}} - t_{\mathrm{L},\widehat{m}}$. A similar but longer argument would allow to show that the following derivations are also valid for other types of polynomial extrapolation functions (e.g. linear), provided a finite number of values in the vicinity of $t_{\mathrm{L},\widehat{m}}$ and $t_{\mathrm{U},\widehat{m}}$, respectively, are used for its fit.

In the above paragraph, $\widehat{f}_V^{\mathrm{LL}}$ denotes the probit local likelihood estimator of Geenens (2014) computed from $V_1^{\widehat{m}}, \ldots, V_{n^+}^{\widehat{m}}$ using a bandwidth h_S and a Gaussian kernel.

That is, with

$$(\widehat{a}_0(s), \widehat{a}_1(s), \widehat{a}_2(s)) = \arg \max_{a_0,a_1,a_2} \sum_{i=1}^{n} \phi\Big(\frac{V_i^{\widehat{m}} - s}{h_S}\Big)\big\{a_0 + a_1(V_i^{\widehat{m}} - s) + a_2(V_i^{\widehat{m}} - s)^2\big\}$$
$$- n \int \phi\Big(\frac{t-s}{h_S}\Big) \exp\{a_0 + a_1(t-s) + a_2(t-s)^2\}\, dt\,,$$

$\widehat{f}_V^{\mathrm{LL}}$ can be expressed as

$$\widehat{f}_V^{\mathrm{LL}}(x) = \Delta^{\widehat{m}} \phi^{-1}\Big\{\Phi^{(-1)}\Big(\frac{x - t_{\mathrm{L},\widehat{m}}}{\Delta^{\widehat{m}}}\Big)\Big\} f_{S^{\widehat{m}}}\Big\{\Phi^{(-1)}\Big(\frac{x - t_{\mathrm{L},\widehat{m}}}{\Delta^{\widehat{m}}}\Big)\Big\} \tag{G.2}$$

where $f_{S^{\widehat{m}}}$ is the local log-quadratic likelihood density estimator satisfying

$$f_{S^{\widehat{m}}}(s) = \exp\{\widehat{a}_0(s)\}\,. \tag{G.3}$$

Following the procedure described in Section 3.2, we study properties of the monotonized version of $\widehat{m}$ defined by $\widehat{m}_M = \{\widehat{m^{(-1)}}\}^{(-1)}$, and following (3.20), we study properties of the estimator of $\{m^{(-1)}\}'$ defined as

$$\{\widehat{m^{(-1)}}\}' = (b-a)\widetilde{f}_V^{\mathrm{LL}}\,. \tag{G.4}$$

The results to be presented are valid under general regularity conditions on the function m and under assumptions related to the uniform almost sure convergence rates/asymptotic order of $\widehat{m}$ and its derivatives on $[a, b]$. These are described below.

Assumption C

(C1) m is strictly increasing and three times continuously differentiable on $\widetilde{\mathcal{I}}$, where $\widetilde{\mathcal{I}} \subset \mathbb{R}$ is an open set that contains $\mathcal{D} \equiv [a, b]$. Moreover, there exists a constant $c_m > 0$ such that $\inf_{x\in\widetilde{\mathcal{I}}} m'(x) \geqslant c_m$.

(C2) There exist sequences κ_n, a_n and b_n satisfying $\max(\kappa_n, b_n) \to 0$, $a_n \to \infty$ and $\max(\kappa_n, b_n)a_n \to 0$ as $n \to \infty$, such that for $\ell \in \{0, 1\}$, $\sup_{z\in\mathcal{D}} |\widehat{m}^{(\ell)}(z) - m^{(\ell)}(z)| = O_{a.s.}(\kappa_n a_n^\ell + b_n)$ as $n \to \infty$.

(C3) With κ_n and a_n as in Condition (C2), for $\ell \in \{0, 1, 2, 3\}$, we have $\sup_{z\in\mathcal{D}} |\widehat{m}^{(\ell)}(z)| = O_{a.s.}(1 + a_n^\ell \kappa_n)$ as $n \to \infty$.

(C4) As $n \to \infty$, $nh_S^3\{\log(n)\}^{-1/2} \to \infty$ and $h_S \log^2(n) \to 0$. Further, there exists $\epsilon > 0$ such that $h_S^2 n^\epsilon a_n^2 \to 0$ as $n \to \infty$, with a_n as in Condition (C2).

(C5) $\lambda \geqslant \log^{1/2}(nh_S)/nh_S^3$, and as $n \to \infty$, $\lambda \to 0$.

The following propositions are the main results of this section. Their proofs rely on a sequence of lemmas that are proved in subsequent sections.

Proposition G.1. *Assume that m is a function satisfying Condition (C1), and that $\widehat{m}$ is an estimator of m that fulfils Condition (C2). With $\widehat{m^{(-1)}}$ defined as in* (G.1), *with h_S and λ satisfying Conditions (C4) and (C5), then, as $n \to \infty$ we have*

$$\sup_{t\in[t_{L,\widehat{m}},t_{U,\widehat{m}}]} |\{\widehat{m^{(-1)}}\}'(t) - \{m^{(-1)}\}'(t)| = O_{a.s.}\{\lambda + \log(n)(e^{\gamma_n} h_S^2 + \kappa_n a_n + b_n)\}\,,$$

where $\gamma_n = \sqrt{2}\log^{1/2}[nh_S^3\{\sqrt{2\pi}\log^{1/2}(nh_S)\}^{-1}]$.

Proof of Proposition G.1: Recall from (G.2) that

$$\widehat{f}_V^{\mathrm{LL}}(x) = \Delta^{\widehat{m}}\phi^{-1}\Big\{\Phi^{(-1)}\Big(\frac{x - t_{\mathrm{L},\widehat{m}}}{\Delta^{\widehat{m}}}\Big)\Big\} f_{S^{\widehat{m}}}\Big\{\Phi^{(-1)}\Big(\frac{x - t_{\mathrm{L},\widehat{m}}}{\Delta^{\widehat{m}}}\Big)\Big\}\,.$$

Put $\mathfrak{K}_j(u) = u^j\phi(u)$ and let

$$S_{n,j}^{\widehat{m}}(s) = (n^+h_S)^{-1}\sum_{i=1}^{n^+}\mathfrak{K}_j\Bigg(\frac{\Phi^{-1}\Big\{\frac{\widehat{m}(U_i)-t_{\mathrm{L},\widehat{m}}}{\Delta^{\widehat{m}}}\Big\} - s}{h_S}\Bigg)\,. \tag{G.5}$$

In this notation, in view of Lemma G.12 in Section G.3, we can write $f_{S^{\widehat{m}}}$ at (G.3) as

$$f_{S^{\widehat{m}}}(s) = S_{n,0}^{\widehat{m}}(s)\Big\{\frac{\{S_{n,0}^{\widehat{m}}(s)\}^2}{S_{n,2}^{\widehat{m}}(s)S_{n,0}^{\widehat{m}}(s) - \{S_{n,1}^{\widehat{m}}(s)\}^2}\Big\}^{1/2}\exp\Big(\frac{-\{S_{n,1}^{\widehat{m}}(s)\}^2/2}{S_{n,2}^{\widehat{m}}(s)S_{n,0}^{\widehat{m}}(s) - \{S_{n,1}^{\widehat{m}}(s)\}^2}\Big)\,.$$

Since $f_{S^{\widehat{m}}}$ matches the definition of $f^{\widehat{m}}$ in Lemma G.6 in Section G.1, we deduce from that lemma that under Conditions (C1), (C2) and (C4), we have, uniformly in $|s| \leqslant \gamma_n$, that, as $n \to \infty$,

$$|f_{S^{\widehat{m}}}(s) - \Delta^{-1}\widetilde{E}^m(s)| = O_{a.s.}\{\log(n)(e^{\gamma_n}h_S^2 + \kappa_n a_n + b_n)\}\,,$$

where

$$\widetilde{E}^m(s) = \frac{\Delta^{\widehat{m}}\phi(s)}{m'[m^{(-1)}\{\Phi(s)\Delta^{\widehat{m}} + t_{\mathrm{L},\widehat{m}}\}]} = [m^{(-1)}\{\Phi(s)\Delta^{\widehat{m}} + t_{\mathrm{L},\widehat{m}}\}]' \,. \tag{G.6}$$

Hence, since $t \in [t_{\mathrm{L},\widehat{m}} + (nh_S^3)^{-1}\log^{1/2}(nh_S)\Delta^{\widehat{m}}, t_{\mathrm{U},\widehat{m}} - (nh_S^3)^{-1}\log^{1/2}(nh_S)\Delta^{\widehat{m}}]$ implies that $|\Phi^{(-1)}\{(t - t_{\mathrm{L},\widehat{m}})/(\Delta^{\widehat{m}})\}| \leqslant \gamma_n$, and since in view of Condition (C5) we have $[t_{\mathrm{L},\widehat{m}} + \lambda\Delta^{\widehat{m}}, t_{\mathrm{U},\widehat{m}} - \lambda\Delta^{\widehat{m}}] \subset [t_{\mathrm{L},\widehat{m}} + (nh_S^3)^{-1}\log^{1/2}(nh_S)\Delta^{\widehat{m}}, t_{\mathrm{U},\widehat{m}} - (nh_S^3)^{-1}$ $\log^{1/2}(nh_S)\Delta^{\widehat{m}}]$, we deduce from the above discussion that we have, uniformly in $t \in [t_{\mathrm{L},\widehat{m}} + \lambda\Delta^{\widehat{m}}, t_{\mathrm{U},\widehat{m}} - \lambda\Delta^{\widehat{m}}]$,

$$(b-a)\widehat{f}_V^{\mathrm{LL}}(t) = \{m^{(-1)}\}'(t) + O_{a.s.}\{\log(n)(e^{\gamma_n}h_S^2 + \kappa_n a_n + b_n)\} \,.$$

Using Taylor expansions, under Condition (C1) we have that $\{m^{(-1)}\}'(t_{\mathrm{L},\widehat{m}} + \lambda\Delta^{\widehat{m}}) = m^{(-1)}(t) + O_{a.s.}(\lambda)$ uniformly in $t \in [t_{\mathrm{L},\widehat{m}}, t_{\mathrm{L},\widehat{m}} + \lambda\Delta^{\widehat{m}}]$ and $\{m^{(-1)}\}'(t_{\mathrm{U},\widehat{m}} - \lambda\Delta^{\widehat{m}}) = m^{(-1)}(t) + O_{a.s.}(\lambda)$ uniformly in $t \in [t_{\mathrm{U},\widehat{m}} - \lambda\Delta^{\widehat{m}}, t_{\mathrm{U},\widehat{m}}]$. We deduce from the definition of $\check{f}_V^{\mathrm{LL}}$ that we have, uniformly in $[t_{\mathrm{L},\widehat{m}}, t_{\mathrm{U},\widehat{m}}]$,

$$(b-a)\check{f}_V^{\mathrm{LL}}(t) = \{m^{(-1)}\}'(t) + O_{a.s.}\{\lambda + \log(n)(e^{\gamma_n}h_S^2 + \kappa_n a_n + b_n)\} \,.$$

We conclude the proof by noting that

$$\int_{t_{\mathrm{L},\widehat{m}}}^{t_{\mathrm{L},\widehat{m}}} \{m^{(-1)}\}'(s)\mathrm{d}s = m^{(-1)}(t_{\mathrm{L},\widehat{m}}) - m^{(-1)}(t_{\mathrm{U},\widehat{m}}) = b - a + O_{a.s.}(\kappa_n + b_n) \,.$$

□

Proposition G.2. *Assume that m is a function satisfying Condition (C1), and that $\widehat{m}$ is an estimator of m that fulfils Conditions (C2) and (C3). With $\widehat{m^{(-1)}}$ and $\widehat{m}_M$ defined at page 69, with h_S and λ satisfying Conditions (C4) and (C5), and with γ_n as in the statement of Proposition G.1, then, as $n \to \infty$ we have*

$$\max\Big\{\sup_{t\in[t_{L,\widehat{m}}, t_{U,\widehat{m}}]}\big|\widehat{m^{(-1)}}(t) - m^{(-1)}(t)\big|, \sup_{x\in[a,b]}\big|\widehat{m}_M(x) - m(x)\big|\Big\}$$
$$= O_{a.s.}(\kappa_n + b_n + \lambda) + |\Phi^{(-1)}(\lambda)|O_{a.s.}\big[h_S^2 e^{\gamma_n}\big\{\log(n) + \kappa_n a_n^3\big\}\big] \,.$$

Proof of Proposition G.2: Recall from the proof of Proposition G.1 that $f_{S^{\widehat{m}}}$ fits the setup of Lemma G.5 (it matches the definition of $f^{\widehat{m}}$ therein). Hence, we obtain from that lemma that, uniformly in $z, z' \in [-\gamma_n, \gamma_n]$ and under Conditions (C1) to (C4),

$$(b-a)\int_z^{z'} f_{S^{\widehat{m}}}(s)\,\mathrm{d}s = m^{(-1)}\{\Phi(z)\Delta^{\widehat{m}} + t_{\mathrm{L},\widehat{m}})\} - m^{(-1)}\{\Phi(z')\Delta^{\widehat{m}} + t_{\mathrm{L},\widehat{m}}\}$$
$$+ O_{a.s.}(\kappa_n + b_n) + |z - z'| O_{a.s.}\big[h_S^2 e^{\gamma_n}\big\{\log(n) + \kappa_n a_n^3\big\}\big]\,. \quad \text{(G.7)}$$

Now note from (G.1) and the expression of $\widehat{f}_V^{\mathrm{LL}}$ at (G.2) that

$$\int_{t_{\mathrm{L},\widehat{m}}}^{t} \mathbb{I}(z \in [t_{\mathrm{L},\widehat{m}} + \lambda\Delta^{\widehat{m}}, t_{\mathrm{U},\widehat{m}} - \lambda\Delta^{\widehat{m}}])\widehat{f}_V^{\mathrm{LL}}(z)\,\mathrm{d}z$$
$$= \mathbb{I}(t \geqslant t_{\mathrm{L},\widehat{m}} + \Delta^{\widehat{m}}\lambda)\int_{\Phi^{(-1)}(\lambda)}^{\Phi^{(-1)}[\{\min(t, t_{\mathrm{U},\widehat{m}} - \lambda\Delta^{\widehat{m}}) - t_{\mathrm{L},\widehat{m}}\}/\Delta^{\widehat{m}}]} f_{S^{\widehat{m}}}(s)\,\mathrm{d}s\,. \quad \text{(G.8)}$$

From Section 7.1 in Feller (1968), for $x > 1$ we have $\Phi(-x) \leqslant (2\pi)^{-1/2}\exp(-x^2/2)$. Under Condition (C5), we have $nh_S^3 \log^{-1/2}(nh_S) \to \infty$ as $n \to \infty$, which implies that $\gamma_n \to \infty$ as $n \to \infty$. We deduce that, as $n \to \infty$, $\Phi(-\gamma_n) \leqslant (nh_S^3)^{-1}\log^{1/2}(nh_S) \leqslant \lambda$ (see Condition (C5) for the last inequality). Since $\Phi(-x) = 1 - \Phi(x)$, we also have $\Phi(\gamma_n) \geqslant 1 - (nh_S^3)^{-1}\log^{1/2}(nh_S) \geqslant 1 - \lambda$. Therefore, as $n \to \infty$ we have $[\Phi^{(-1)}(\lambda), \Phi^{(-1)}(1-\lambda)] \subseteq [-\gamma_n, \gamma_n]$. Using (G.7) and (G.9), we deduce that, uniformly in $t \in [t_{\mathrm{L},\widehat{m}}, t_{\mathrm{U},\widehat{m}}]$, we have, as $n \to \infty$,

$$(b-a)\int_{t_{\mathrm{L},\widehat{m}}}^{t} \mathbb{I}(z \in [t_{\mathrm{L},\widehat{m}} + \lambda\Delta^{\widehat{m}}, t_{\mathrm{U},\widehat{m}} - \lambda\Delta^{\widehat{m}}])\widehat{f}_V^{\mathrm{LL}}(z)\,\mathrm{d}z$$
$$= \mathbb{I}(t \geqslant t_{\mathrm{L},\widehat{m}} + \Delta^{\widehat{m}}\lambda)\big[m^{(-1)}\{\min(t, t_{\mathrm{U},\widehat{m}} - \lambda\Delta^{\widehat{m}})\} - m^{(-1)}(t_{\mathrm{L},\widehat{m}} + \lambda\Delta^{\widehat{m}})\big]$$
$$+ O_{a.s.}(\kappa_n + b_n) + |\Phi^{(-1)}(\lambda)| O_{a.s.}\big[h_S^2 e^{\gamma_n}\big\{\log(n) + \kappa_n a_n^3\big\}\big]\,. \quad \text{(G.9)}$$

Now Lemma G.11 in Section G.3 implies that $\widehat{m}$ is a.s. strictly increasing for n sufficiently large. In view of Condition (C2), this implies that $t_{\mathrm{L},\widehat{m}} \overset{a.s.}{=} \widehat{m}(b) = L(b) + o_{a.s.}(1)$ and $t_{\mathrm{U},\widehat{m}} \overset{a.s.}{=} \widehat{m}(a) = L(a) + o_{a.s.}(1)$ a.s. We deduce that for any $\epsilon > 0$ and n sufficiently large, we have, for any $z \in [0,1]$, $L(a) - \epsilon \leqslant z\Delta^{\widehat{m}} + t_{\mathrm{L},\widehat{m}} = zL(b) + (1-z)L(a) + o_{a.s.}(1) \leqslant L(b) + \epsilon$. This implies that as $n \to \infty$, $m^{(-1)}(z\Delta^{\widehat{m}} + t_{\mathrm{L},\widehat{m}}) \in \widetilde{\mathcal{I}}$

a.s. for any $z \in [0,1]$, with $\widetilde{\mathcal{I}}$ as in Condition (C1). Hence, since $\lambda \in (0,1/2)$, we deduce from the mean-value theorem that as $n \to \infty$,

$$\begin{aligned} \left|m^{(-1)}(\lambda\Delta^{\widehat{m}} + t_{\mathrm{L},\widehat{m}}) - a\right| &= \left|m^{(-1)}(\lambda\Delta^{\widehat{m}} + t_{\mathrm{L},\widehat{m}}) - m^{(-1)}\{m(a)\}\right| \\ &\leqslant \Big\{\inf_{x\in\widetilde{\mathcal{I}}} m'(x)\Big\}^{-1} |\lambda + t_{\mathrm{L},\widehat{m}} - m(a)| \\ &\leqslant c_m^{-1}\lambda + |t_{\mathrm{L},\widehat{m}} - m(a)| = O_{a.s.}(\kappa_n + b_n + \lambda)\,. \end{aligned} \tag{G.10}$$

To obtain the inequality on the last line we used Condition (C1), which implies that $m' \geqslant c_m > 0$ on $\widetilde{\mathcal{I}}$. To obtain the last equality in (G.10), we used the fact c_m is a constant, and Condition (C2), which implies that $|\widehat{m} - m| = O_{a.s.}(\kappa_n + b_n)$ on $[a,b]$ for n sufficiently large (recall from above (G.10) that $t_{\mathrm{L},\widehat{m}} = \widehat{m}(a)$ a.s. as $n \to \infty$).

Therefore, from (G.9), we obtain that

$$\begin{aligned} &(b-a)\int_{t_{\mathrm{L},\widehat{m}}}^{t} \mathbb{I}(z \in [t_{\mathrm{L},\widehat{m}} + \lambda\Delta^{\widehat{m}}, t_{\mathrm{U},\widehat{m}} - \lambda\Delta^{\widehat{m}}])\widehat{f}_V^{\mathrm{LL}}(z)\,\mathrm{d}z \\ &\quad= \mathbb{I}(t \geqslant t_{\mathrm{L},\widehat{m}} + \Delta^{\widehat{m}}\lambda)\big[m^{(-1)}\{\min(t, t_{\mathrm{U},\widehat{m}} - \lambda\Delta^{\widehat{m}})\} - a\big] \\ &\qquad+ O_{a.s.}(\kappa_n + b_n + \lambda) + |\Phi^{(-1)}(\lambda)| O_{a.s.}\big[h_S^2 e^{\gamma_n}\big\{\log(n) + \kappa_n a_n^3\big\}\big]\,. \end{aligned} \tag{G.11}$$

Since

$$\begin{aligned} \int_{t_{\mathrm{L},\widehat{m}}}^{t} \check{f}_V^{\mathrm{LL}}(z)\,\mathrm{d}z =& \{\min(t, t_{\mathrm{L},\widehat{m}} + \lambda\Delta^{\widehat{m}}) - t_{\mathrm{L},\widehat{m}}\}\widehat{f}_V^{\mathrm{LL}}(t_{\mathrm{L},\widehat{m}} + \lambda\Delta^{\widehat{m}}) \\ &+ \mathbb{I}(t \geqslant t_{\mathrm{L},\widehat{m}} + \lambda\Delta^{\widehat{m}}) \int_{\Phi^{(-1)}(\lambda)}^{\Phi^{(-1)}\{(\min(t, t_{\mathrm{U},\widehat{m}} - \lambda\Delta^{\widehat{m}}) - t_{\mathrm{L},\widehat{m}})/\Delta^{\widehat{m}}\}} f_{S^{\widehat{m}}}(s)\,\mathrm{d}s \\ &+ \mathbb{I}(t \geqslant t_{\mathrm{U},\widehat{m}} + \lambda\Delta^{\widehat{m}})(t - t_{\mathrm{U},\widehat{m}} + \lambda\Delta^{\widehat{m}})\widehat{f}_V^{\mathrm{LL}}(t_{\mathrm{U},\widehat{m}} - \lambda\Delta^{\widehat{m}})\,, \end{aligned} \tag{G.12}$$

and since from the proof of Proposition G.1 we have, uniformly in $t \in [-\gamma_n, \gamma_n]$, that $\Delta\widehat{f}_V^{\mathrm{LL}}(t) = \{m^{(-1)}\}'(t) + O_{a.s.}\{\lambda + \log(n)(e^{\gamma_n} h_S^2 + \kappa_n a_n + b_n)\}$, we obtain from (G.11) and (G.12) that

$$(b-a)\int_{t_{\mathrm{L},\widehat{m}}}^{t} \check{f}_V^{\mathrm{LL}}(z)\,\mathrm{d}z = \tag{G.13}$$

$$
\begin{aligned}
&\{\min(t, t_{\mathrm{L},\widehat{m}} + \lambda\Delta^{\widehat{m}}) - t_{\mathrm{L},\widehat{m}}\}\{m^{(-1)}\}'(t_{\mathrm{L},\widehat{m}} + \lambda\Delta^{\widehat{m}}) \\
&\quad + \mathbb{I}(t \geqslant t_{\mathrm{L},\widehat{m}} + \Delta^{\widehat{m}}\lambda)\big[m^{(-1)}\{\min(t, t_{\mathrm{U},\widehat{m}} - \lambda\Delta^{\widehat{m}})\} - a\big] \\
&\qquad + \mathbb{I}(t \geqslant t_{\mathrm{U},\widehat{m}} + \lambda\Delta^{\widehat{m}})(t_{\mathrm{U},\widehat{m}} - \lambda\Delta^{\widehat{m}} - t)\{m^{(-1)}\}'(t_{\mathrm{U},\widehat{m}} - \lambda\Delta^{\widehat{m}}) \\
&\qquad\quad + O_{a.s.}(\kappa_n + b_n + \lambda) + |\Phi^{(-1)}(\lambda)| O_{a.s.}\big[h_S^2 e^{\gamma_n}\big\{\log(n) + \kappa_n a_n^3\big\}\big]\,. \qquad \text{(G.14)}
\end{aligned}
$$

To obtain (G.13), we also used the result $\lambda O_{a.s.}\{\lambda + \log(n)(e^{\gamma_n}h_S^2 + \kappa_n a_n + b_n)\} = O_{a.s.}(\lambda)$, which follows from our conditions. Moreover, we use the fact that $\Delta^{\widehat{m}} = O_{a.s.}(1)$.

Now, it follows from a Taylor expansion of order two that, uniformly in $t \in [t_{\mathrm{L},\widehat{m}}, t_{\mathrm{L},\widehat{m}} + \lambda\Delta^{\widehat{m}}]$, $m^{(-1)}(t) = m^{(-1)}\{t_{\mathrm{L},\widehat{m}} + \lambda\Delta^{\widehat{m}})\} + m^{(-1)}\{t_{\mathrm{L},\widehat{m}} + \lambda\Delta^{\widehat{m}})\}(t - t_{\mathrm{L},\widehat{m}} - \lambda\Delta^{\widehat{m}}) + O_{a.s.}(\lambda^2)$. We deduce that for any $t \in [t_{\mathrm{L},\widehat{m}}, t_{\mathrm{L},\widehat{m}} + \lambda\Delta^{\widehat{m}}]$, we have, as $n \to \infty$,

$$
(t - t_{\mathrm{L},\widehat{m}})\{m^{(-1)}\}'(t_{\mathrm{L},\widehat{m}} + \lambda\Delta^{\widehat{m}}) = m^{(-1)}(t) - a + O_{a.s.}(\kappa_n + b_n + \lambda)\,,
$$

where we used (G.10).

Using a similar argument, we can prove that $(t_{\mathrm{U},\widehat{m}} - \lambda\Delta^{\widehat{m}} - t)\{m^{(-1)}\}'(t_{\mathrm{U},\widehat{m}} - \lambda\Delta^{\widehat{m}}) = m^{(-1)}(t) - m^{(-1)}\{\min(t, t_{\mathrm{U},\widehat{m}} - \lambda\Delta^{\widehat{m}})\} + O_{a.s.}(\lambda^2)$ uniformly in $t \in [t_{\mathrm{U},\widehat{m}} - \lambda\Delta^{\widehat{m}}, t_{\mathrm{U},\widehat{m}}]$. Therefore, we conclude from (G.13) that we have, uniformly in $t \in [t_{\mathrm{L},\widehat{m}}, t_{\mathrm{U},\widehat{m}}]$,

$$
\begin{aligned}
(b-a)\int_{t_{\mathrm{L},\widehat{m}}}^{t} \check{f}_V^{\mathrm{LL}}(z)\,\mathrm{d}z \;&= m^{(-1)}(t) - a \\
&\qquad + O_{a.s.}(\lambda + \kappa_n + b_n) + |\Phi^{(-1)}(\lambda)| O_{a.s.}\big[h_S^2 e^{\gamma_n}\big\{\log(n) + \kappa_n a_n^3\big\}\big]\,.
\end{aligned}
$$

Hence,

$$
\widehat{m^{(-1)}}(t) - m^{(-1)}(t) = O_{a.s.}(\lambda + \kappa_n + b_n) + |\Phi^{(-1)}(\lambda)| O_{a.s.}\big[h_S^2 e^{\gamma_n}\big\{\log(n) + \kappa_n a_n^3\big\}\big]\,. \qquad \text{(G.15)}
$$

From there, since $\widehat{m^{(-1)}}$ is strictly increasing by construction, and is therefore a one-to-one map on the interval $\mathcal{J} \equiv [t_{\mathrm{L},\widehat{m}}, t_{\mathrm{U},\widehat{m}}]$, we deduce that for the interval $\mathcal{J}_- = [\widehat{m^{(-1)}}(t_{\mathrm{L},\widehat{m}}), \widehat{m^{(-1)}}(t_{\mathrm{U},\widehat{m}})] = [a, b]$, we have, as $n \to \infty$,

$$
\sup_{x \in \mathcal{J}_-} |\widehat{m}_M(x) - m(x)| = \sup_{t \in \mathcal{J}} |t - m\{\widehat{m^{(-1)}}(t)\}|
$$

$$
\begin{aligned}
&= \sup_{t \in \mathcal{J}} |m\{m^{(-1)}(t)\} - m\{\widehat{m^{(-1)}}(t)\}| \\
&\leqslant \sup_{x \in \tilde{\mathcal{I}}} m'(x) \sup_{t \in \mathcal{J}} |m^{(-1)}(t) - \widehat{m^{(-1)}}(t)| \\
&= O_{a.s.}(\lambda + \kappa_n + b_n) + |\Phi^{(-1)}(\lambda)| O_{a.s.}\big[h_S^2 e^{\gamma_n}\big\{\log(n) + \kappa_n a_n^3\big\}\big] .
\end{aligned}
$$

This concludes the proof. □

Proposition G.3. *Assume that m is a function satisfying Condition (C1), and that $\widehat{m}$ is an estimator of m that fulfils Conditions (C2) and (C3). Then, with $\widehat{m}_M$ defined above* (G.1)*, with h_S and λ satisfying Conditions (C4) and (C5), and with γ_n as in the statement of Proposition G.1, as $n \to \infty$ we have that*

$$
\begin{aligned}
&\sup_{x \in [a,b]} \big|\widehat{m}'_M(x) - m'(x)\big| \\
&= O_{a.s.}[\lambda + \log(n)\{\kappa_n a_n + b_n\}] + |\Phi^{(-1)}(\lambda)| O_{a.s.}\big[h_S^2 e^{\gamma_n}\big\{\log(n) + \kappa_n a_n^3\big\}\big] .
\end{aligned}
$$

Proof of Proposition G.3: Since $\widehat{m}_M(x) = \{\widehat{m^{(-1)}}\}^{(-1)}(x)$, we have

$$
\widehat{m}'_M(x) = \frac{1}{\{\widehat{m}^{(-1)}\}'\{\widehat{m}_M(x)\}} .
$$

We deduce from Proposition G.1 that we have,uniformly in $t \in [t_{\mathrm{L},\widehat{m}}, t_{\mathrm{U},\widehat{m}}]$ and as $n \to \infty$,

$$
\{\widehat{m^{(-1)}}\}'(t) - \{m^{(-1)}\}'(t) = O_{a.s.}\{\lambda + \log(n)(e^{\gamma_n} h_S^2 + \kappa_n a_n + b_n)\} ,
$$

where $\{m^{(-1)}\}'(t) = [m'\{m^{(-1)}(x)\}]^{-1} \geqslant c'$ for some $c' > 0$ (see Condition (C1)).

Since the right hand side of the above equation converges to 0 as $n \to \infty$ (see Condition (C4)), we deduce that we have, uniformly in $x \in [a,b]$ and as $n \to \infty$,

$$
\begin{aligned}
\{\widehat{m^{(-1)}}\}'\{\widehat{m}_M(x)\} &\geqslant \{m^{(-1)}\}'\{\widehat{m}_M(x)\} - |\{\widehat{m^{(-1)}}\}'\{\widehat{m}_M(x)\} - \{m^{(-1)}\}'\{\widehat{m}_M(x)\}| \\
&\geqslant c'/2 \quad \text{a.s.}
\end{aligned}
$$

Using

$$
\widehat{m}'_M(x) - m'(x) = \frac{\{m^{(-1)}\}'\{m(x)\} - \{\widehat{m^{(-1)}}\}'\{\widehat{m}_M(x)\}}{\{\widehat{m}^{(-1)}\}'\{\widehat{m}_M(x)\}} ,
$$

we deduce from the above discussion that we have, uniformly in $t \in [t_{\mathrm{L},\widehat{m}}, t_{\mathrm{U},\widehat{m}}]$, and as $n \to \infty$,

$$|\widehat{m}'_M(x) - m'(x)| \leqslant \big|\{m^{(-1)}\}'\{m(x)\} - \{m^{(-1)}\}'\{\widehat{m}_M(x)\}\big| O_{a.s.}(1) + O_{a.s.}\{\lambda + \log(n)(e^{\gamma_n} h_S^2 + \kappa_n a_n + b_n)\}\,.$$

The result follows from the mean value theorem and Proposition G.2, which imply that

$$|\{m^{(-1)}\}'\{m(x)\} - \{m^{(-1)}\}'\{\widehat{m}(x)\}| = O_{a.s.}(\lambda + \kappa_n + b_n) + |\Phi^{(-1)}(\lambda)| O_{a.s.}\big[h_S^2 e^{\gamma_n}\big\{\log(n) + \kappa_n a_n^3\big\}\big]\,.$$

□

G.1 Key Lemmas needed to prove Propositions G.1 to G.3

In this section, we prove three key lemmas for demonstrating Propositions G.1 to G.3. Recall that these results involve a general function $m : \mathbb{R} \to \mathbb{R}$, which is assumed strictly increasing, and an initial estimator $\widehat{m}$ of m to be monotonized (see the beginning of Section G for an introduction to the general setting).

As in the proof of Proposition G.2, put $\mathfrak{K}_j(u) = u^j\phi(u)$ and $\Delta^{\widehat{m}} = t_{\mathrm{U},\widehat{m}} - t_{\mathrm{L},\widehat{m}}$. Also, recall the definition of $S^{\widehat{m}}_{n,j}$ at (G.5) and let

$$E^{\widehat{m}}(s) = \frac{\Delta^{\widehat{m}}\phi(s)}{\widehat{m}'\big[\widehat{m}^{(-1)}\big\{\Phi(s)\Delta^{\widehat{m}} + t_{\mathrm{L},\widehat{m}}\big\}\big]}\,. \tag{G.16}$$

and

$$f^{\widehat{m}}(s) = S^{\widehat{m}}_{n,0}(s)\Big\{\frac{\{S^{\widehat{m}}_{n,0}(s)\}^2}{S^{\widehat{m}}_{n,2}(s)S^{\widehat{m}}_{n,0}(s) - \{S^{\widehat{m}}_{n,1}(s)\}^2}\Big\}^{1/2} \exp\Big(\frac{-\{S^{\widehat{m}}_{n,1}(s)\}^2/2}{S^{\widehat{m}}_{n,2}(s)S^{\widehat{m}}_{n,0}(s) - \{S^{\widehat{m}}_{n,1}(s)\}^2}\Big) \tag{G.17}$$

Lemma G.4. *Assume that m is a function satisfying Condition (C1), and that $\widehat{m}$ is an estimator of m that fulfils Conditions (C2) and (C3). Then, if h_S and λ satisfy*

Conditions (C4) and (C5), as $n \to \infty$ we have

$$\sup_{|s| \leqslant \gamma_n} |f^{\widehat{m}}(s) - (b-a)^{-1} E^{\widehat{m}}(s)| = O_{a.s.}\big[h_S^2 e^{\gamma_n}\big\{\log(n) + \kappa_n a_n^3\big\}\big] ,$$

where $\gamma_n = (2\log[nh_S^3\{\sqrt{2\pi}\log^{1/2}(nh_S)\}^{-1}])^{1/2}$.

Proof of Lemma G.4: From the definition of γ_n in the statement of the lemma, we have

$$|s| \leqslant \gamma_n \quad \Longleftrightarrow \quad h_S^2\phi(s) \geqslant (nh_S)^{-1}\log^{1/2}(n) . \tag{G.18}$$

Let $\widetilde{E}^{\widehat{m}} = (b-a)^{-1}E^{\widehat{m}}$ and that Condition (C4) implies that $\gamma_n \leqslant \log^{1/2}(n)$ as $n \to \infty$. Combining Lemmas G.7 and G.8 in Section G.2, we deduce that, as $n \to \infty$, we have, for $j \in \{0, 2\}$ and uniformly in $|s| \leqslant \gamma_n$,

$$\begin{aligned}\big|S_{n,j}^{\widehat{m}}(s) - \widetilde{E}^{\widehat{m}}(s)\big| &= \phi(|s|-1)O_{a.s.}\big[h_S^2\{1 + (\gamma_n)^2 + \kappa_n a_n^3\}\big] + O_{a.s.}\Big\{\frac{\log^{1/2}(n)}{nh_S}\Big\} \\ &\leqslant \phi(|s|-1)O_{a.s.}\big[h_S^2\big\{\log(n) + \kappa_n a_n^3\big\}\big] + \phi(s)O_{a.s.}(h_S^2) , \end{aligned} \tag{G.19}$$

and

$$\big|S_{n,1}^{\widehat{m}}(s)\big| = O_{a.s.}\Big[h_S\phi(s)\big\{\log^{1/2}(n) + \kappa_n a_n^2\big\} + h_S^2\phi(|s|-1)\big\{\log(n) + \kappa_n a_n^3\big\}\Big] . \tag{G.20}$$

To obtain these results, we used (G.18).

Since $\phi(|s|-1)/\phi(s) = e^{|s|-1/2}$, we have $\sup_{|s|\leqslant\gamma_n}\phi(|s|-1)/\phi(s) \leqslant e^{\gamma_n}$. Hence, we deduce from (G.19) and (G.20) that

$$\big|S_{n,j}^{\widehat{m}}(s) - \widetilde{E}^{\widehat{m}}(s)\big| = \phi(s)O_{a.s.}\big[h_S^2e^{\gamma_n}\big\{\log(n) + \kappa_n a_n^3\big\}\big] \tag{G.21}$$

and that

$$\begin{aligned}\big|S_{n,1}^{\widehat{m}}(s)\big|^2 &= \phi^2(s)O_{a.s.}\Big[h_S^2\big\{\log(n) + \kappa_n^2 a_n^4\big\} + h_S^4 e^{\gamma_n}\big\{\log^2(n) + \kappa_n^2 a_n^6\big\}\Big] \\ &= \phi^2(s)O_{a.s.}\Big[h_S^2\big\{\log(n) + \kappa_n^2 a_n^4\big\}\Big] . \end{aligned} \tag{G.22}$$

To obtain the first line at (G.22), we used the identify $(a+b)^2 = O(a^2+b^2)$. The second line at (G.22) is a consequence of the fact that Condition (A4) implies that $h_S^2 e^{\gamma_n}(\log^2(n)+a_n^2) \to 0$ as $n \to \infty$. Indeed, the fact that $h_S \leqslant 1$ and $\log(n) \geqslant 1$ for n sufficiently large (see Condition (A4)) implies that for any $\epsilon > 0$, $e^{\gamma_n} = (e^{\gamma_n^2/2})^{2/\gamma_n} \leqslant n^{2/\gamma_n} \leqslant n^{\epsilon}$ as $n \to \infty$ (here we used the fact that Condition (A4) implies that $\gamma_n \to \infty$ as $n \to \infty$).

Therefore, combining the inequality $|S_{n,0}^{\widehat{m}} S_{n,2}^{\widehat{m}} - (\widetilde{E}^{\widehat{m}})^2| \leqslant |S_{n,0}^{\widehat{m}} - \widetilde{E}^{\widehat{m}}| S_{n,2}^{\widehat{m}} + |S_{n,2}^{\widehat{m}} - \widetilde{E}^{\widehat{m}}| \widetilde{E}^{\widehat{m}}$ with (G.21) and (G.22) , we deduce that, as $n \to \infty$, we have, uniformly in $|s| \leqslant \gamma_n$,

$$S_{n,0}^{\widehat{m}}(s) S_{n,2}^{\widehat{m}}(s) - \{S_{n,1}^{\widehat{m}}(s)\}^2 = \{\widetilde{E}^{\widehat{m}}(s)\}^2 + \phi^2(s)\xi_n(s)\,,$$

with $\sup_{|s|\leqslant\gamma_n} |\xi_n(s)| = O_{a.s.}(h_S^2 e^{\gamma_n}[\log(n) + \kappa_n a_n^3])$. Here we used the fact that for n sufficiently large, $\widehat{m}' \geqslant \kappa > 0$ a.s. on $[a,b]$ for some $\kappa > 0$ (see Lemma G.11 in Section G.3), which implies that $|\widetilde{E}^{\widehat{m}}(s)| = \phi(s) O_{a.s.}(1)$.

From there, Lemma G.11 in Section G.3 implies the existence of a constant $c > 0$ such that $\widehat{m}' \leqslant c^{-1}$ a.s. on $[a,b]$ and that $\widehat{m}^{(-1)}\{\Phi(s)\Delta^{\widehat{m}} + t_{\mathrm{L},\widehat{m}}\} \in [a,b]$ a.s., which implies that $\widetilde{E}^{\widehat{m}} \geqslant \phi(s)c$ a.s. as $n \to \infty$. Since Condition (C4) implies that $\sup_{|s|\leqslant\gamma_n} |\xi_n(s)| = o_{a.s.}(1)$ as $n \to \infty$, we have $\{\widetilde{E}^{\widehat{m}}(s)\}^2 + \phi^2(s)\xi_n(s) = \{\widetilde{E}^{\widehat{m}}(s)\}^2\{1 + o_{a.s.}(1)\}$ uniformly in $|s| \leqslant \gamma_n$. Therefore, we have, uniformly in $|s| \leqslant \gamma_n$ and as $n \to \infty$,

$$\begin{aligned} S_{n,0}^{\widehat{m}}(s) S_{n,2}^{\widehat{m}}(s) - \{S_{n,1}^{\widehat{m}}(s)\}^2 &= \{\widetilde{E}^{\widehat{m}}(s)\}^2\{1 + o_{a.s.}(1)\} \\ &\geqslant (b-a)^{-2} c^{-2} \phi^2(s)\{1 + o_{a.s.}(1)\}\,. \end{aligned} \tag{G.23}$$

The latter inequality combined with (G.22) implies that, as $n \to \infty$,

$$\sup_{|s|\leqslant\gamma_n} \frac{|S_{n,1}^{\widehat{m}}(s)|^2}{S_{n,0}^{\widehat{m}}(s) S_{n,2}^{\widehat{m}}(s) - \{S_{n,1}^{\widehat{m}}(s)\}^2} = O_{a.s.}\Big(h_S^2\big(\log(n) + \kappa_n^2 a_n^4\big)\Big)\,.$$

Combining the latter result to the mean value theorem proves that, as $n \to \infty$,

$$\sup_{|s|\leqslant\gamma_n} \Big| \exp\Big(\frac{-\{S_{n,1}^{\widehat{m}}(s)\}^2}{2\{S_{n,2}^{\widehat{m}}(s) S_{n,0}^{\widehat{m}}(s) - \{S_{n,1}^{\widehat{m}}(s)\}^2\}}\Big) - 1\Big| = O_{a.s.}\Big(h_S^2\big(\log(n) + \kappa_n^2 a_n^4\big)\Big)\,.$$

Moreover, in view of

$$\frac{\{S^{\widehat{m}}_{n,0}(s)\}^2}{S^{\widehat{m}}_{n,2}(s)S^{\widehat{m}}_{n,0}(s)-\{S^{\widehat{m}}_{n,1}(s)\}^2}=1+\frac{S^{\widehat{m}}_{n,0}(s)\{S^{\widehat{m}}_{n,0}(s)-S^{\widehat{m}}_{n,2}(s)\}+\{S^{\widehat{m}}_{n,1}(s)\}^2}{S^{\widehat{m}}_{n,2}(s)S^{\widehat{m}}_{n,0}(s)-\{S^{\widehat{m}}_{n,1}(s)\}^2}\,,$$

(G.22), (G.21) and (G.23) imply that we have, as $n\to\infty$,

$$\sup_{|s|\leqslant\gamma_n}\left|\frac{\{S^{\widehat{m}}_{n,0}(s)\}^2}{S^{\widehat{m}}_{n,2}(s)S^{\widehat{m}}_{n,0}(s)-\{S^{\widehat{m}}_{n,1}(s)\}^2}-1\right|=O_{a.s.}\big(h_S^2e^{\gamma_n}\big[\log(n)+\kappa_n a_n^3\big]\big)\,,$$

so that, using the mean-value theorem,

$$\sup_{|s|\leqslant\gamma_n}\left|\left\{\frac{\{S^{\widehat{m}}_{n,0}(s)\}^2}{S^{\widehat{m}}_{n,2}(s)S^{\widehat{m}}_{n,0}(s)-\{S^{\widehat{m}}_{n,1}(s)\}^2}\right\}^{1/2}-1\right|=O_{a.s.}\big(h_S^2e^{\gamma_n}\big[\log(n)+\kappa_n a_n^3\big]\big)\,.$$

Combining the latter discussion to (G.19) concludes the proof of the lemma. □

Lemma G.5. *Assume that m is a function satisfying Condition (C1), and that $\widehat{m}$ is an estimator of m that fulfils Conditions (C2) and (C3). Then, if h_S and λ satisfy Conditions (C4) and (C5), and with γ_n as in the statement of Lemma G.4, we have, uniformly in $z,z'\in[-\gamma_n,\gamma_n]$ and as $n\to\infty$,*

$$\begin{aligned}(b-a)\int_z^{z'}f^{\widehat{m}}(s)\,\mathrm{d}s=&m^{(-1)}\{\Phi(z')\Delta^{\widehat{m}}+t_{L,\widehat{m}}\}-m^{(-1)}\{\Phi(z)\Delta^{\widehat{m}}+t_{L,\widehat{m}}\}\\&+O_{a.s.}(\kappa_n+b_n)+|z-z'|O_{a.s.}\big[h_S^2e^{\gamma_n}\{\log(n)+\kappa_n a_n^3\}\big]\,.\end{aligned}$$

Proof of Lemma G.5: From the definition of $E^{\widehat{m}}$ at (G.16) we have

$$\begin{aligned}\int_z^{z'}E^{\widehat{m}}(s)\mathrm{d}s&=\widehat{m}^{(-1)}\{\Phi(z')\Delta^{\widehat{m}}+t_{\mathrm{L},\widehat{m}}\}-\widehat{m}^{(-1)}\{\Phi(z)\Delta^{\widehat{m}}+t_{\mathrm{L},\widehat{m}}\}\\&=m^{(-1)}\{\Phi(z')\Delta^{\widehat{m}}+t_{\mathrm{L},\widehat{m}}\}-m^{(-1)}\{\Phi(z)\Delta^{\widehat{m}}+t_{\mathrm{L},\widehat{m}}\}+O_{a.s.}(\kappa_n+b_n)\,,\qquad\text{(G.24)}\end{aligned}$$

where, to obtain the last line, we used Lemma G.11 in Section G.3, which implies that $\widehat{m}^{(-1)}-m^{(-1)}=O_{a.s.}(\kappa_n+b_n)$ on $[t_{\mathrm{L},\widehat{m}},t_{\mathrm{U},\widehat{m}}]$.

From there, the result follows Lemma G.4, which implies that under Conditions (C1) to (C5), we have, uniformly in $|s|\leqslant\gamma_n$ and as $n\to\infty$,

$$|(b-a)f^{\widehat{m}}(s)-E^{\widehat{m}}(s)|=O_{a.s.}\big(h_S^2e^{\gamma_n}\big[\log(n)+\kappa_n a_n^3\big]\big)\,.$$

□

Recall the definition of $\widetilde{E}^m$ at (G.6).

Lemma G.6. *Assume that m is a function satisfying Condition (C1), and that $\widehat{m}$ is an estimator of m that fulfils Condition (C2). Then, if h_S satisfies Condition (C4), and with γ_n as in the statement of Lemma G.4, as $n \to \infty$ we have*

$$\sup_{|s|\leqslant\gamma_n} |f^{\widehat{m}}(s) - (b-a)^{-1}\widetilde{E}^m(s)| = O_{a.s.}\{\log(n)(e^{\gamma_n}h_S^2 + \kappa_n a_n + b_n)\}\,.$$

Proof of Lemma G.6: The proof is very similar to that of Lemma G.4. Recall from (G.18) in the proof of that lemma that

$$|s| \leqslant \gamma_n \quad \iff \quad h_S^2\phi(s) \geqslant (n^+)^{-1}h_S^{-1}\log^{1/2}(nh_S)\,. \tag{G.25}$$

Next, since

$$(\log n)\phi(s) \geqslant \phi\Big(s\frac{\sqrt{1-h_S^2}}{\sqrt{1+h_S^2}}\Big) \quad \iff \quad |s| \leqslant h_S^{-1}\sqrt{1+h_S^2}\log\log n\,,$$

and since $\gamma_n \leqslant h_S^{-1}\sqrt{1+h_S^2}\log\log n$ as $n \to \infty$ (see Condition (C4)), we deduce from Lemma G.10 that as $n \to \infty$, we have, uniformly in $|s| \leqslant \gamma_n$,

$$\big|\mathcal{J}_j^{\widehat{m}}(s) - \widetilde{\mathcal{J}}_j^m(s)\big| = \phi(s)O_{a.s.}(\kappa_n a_n + b_n)\,.$$

From there, since $|s| \leqslant \gamma_n$ implies that $e^{\gamma_n}\phi(s) \geqslant \phi(|s|-1)$ uniformly in $|s| \leqslant \gamma_n$ (see the argument above (G.21) in the proof of Lemma G.4), and since $\gamma_n \leqslant \log^{1/2}(n)$ as $n \to \infty$, we obtain by combining the above equation with Lemmas G.7 and G.10 in Section G.3 that, as $n \to \infty$, we have, uniformly in $|s| \leqslant \gamma_n$ and for $j \in \{0,2\}$,

$$\begin{aligned}\big|S_{n,j}^{\widehat{m}}(s) - (b-a)^{-1}\widetilde{E}^L(s)\big| &= \phi(s)\log(n)O_{a.s.}(e^{\gamma_n}h_S^2 + \kappa_n a_n + b_n) + O_{a.s.}\Big(\frac{\sqrt{\log(n)}}{nh_S}\Big)\\ &= \phi(s)\log(n)O_{a.s.}(e^{\gamma_n}h_S^2 + \kappa_n a_n + b_n)\,.\end{aligned} \tag{G.26}$$

To obtain the last equality, we used (G.18).

Using similar arguments, we obtain that

$$\big|S_{n,1}^{\widehat{m}}(s)\big| = \phi(s)O_{a.s.}\Big\{h_S\log^{1/2}(n) + \kappa_n a_n + b_n + h_S^2e^{\gamma_n}\log(n)\Big\} + O_{a.s.}\Big(\frac{\sqrt{\log(n)}}{nh_S}\Big)$$

$$= \phi(s) O_{a.s.}\Big\{ h_S \log^{1/2}(n) + \kappa_n a_n + b_n \Big\}\,, \tag{G.27}$$

where we also used Condition (C4), which implies that $h_S e^{\gamma_n} \log^{1/2}(n) \to 0$ as $n \to \infty$.

From there, to conclude the proof of the lemma we can employ the same arguments as those used to conclude the proof of Lemma G.4 (those below (G.20)) with (G.26) and (G.27) instead of (G.19) and (G.20). □

G.2 Technical lemmas required for the results in Section G.1

This section covers four technical lemmas, which are required to demonstrate the results in Section G.1. These results involve a general function $m : \mathbb{R} \to \mathbb{R}$, which is assumed strictly increasing, and an initial estimator $\widehat{m}$ of m to be monotonized (see the beginning of Section G for an introduction to the general setting).

In this section, we will need the following quantity:

$$\mathcal{J}_j^{\widehat{m}}(s) = \frac{1}{(b-a)h_S} \int_a^b \mathfrak{K}_j \Big(\frac{\Phi^{-1}\Big\{ \frac{\widehat{m}(u) - t_{\mathrm{L},\widehat{m}}}{\Delta^{\widehat{m}}} \Big\} - s}{h_S} \Big) \mathrm{d}u\,. \tag{G.28}$$

Lemma G.7. *Assume that m is a function satisfying Condition (C1), and that $\widehat{m}$ is an estimator of m that fulfils Condition (C2). Then, if h_S satisfies Condition (C4), we have uniformly in $|s| \leqslant \sqrt{8\log(nh_S)}$ that*

$$|S_{n,j}^{\widehat{m}}(s) - \mathcal{J}_j^{\widehat{m}}(s)| = O_{a.s.}\{(nh_S)^{-1} \log^{1/2}(n)\} \qquad \text{as } n \to \infty. \tag{G.29}$$

Proof of Lemma G.7: In what follows, to simplify the exposition, we drop the index $+$ of n^+ and write $n^+ \equiv n$.

First, under Conditions (C1) and (C2), Lemma G.11 in Section G.3 implies that for c_m as in Condition (C1), for n sufficiently large we have with probability one that

$$\inf_{x \in [a,b]} \widehat{m}'(x) \geqslant c_m/2\,. \tag{G.30}$$

When (G.30) holds, $\widehat{m}$ is strictly increasing on $[a, b]$, and the inverse of $\widehat{m}$ is a well-defined strictly increasing function on $[\widehat{m}(a), \widehat{m}(b)]$. We will assume through that n is sufficiently large to ensure that (G.30) holds with probability 1.

For $\gamma \in (\sqrt{2}, \sqrt{8\log(nh_S)})$, let

$$\mathcal{J}_j^{\widehat{m}}(s;\gamma) = h_S^{-1} \int_{\widehat{m}^{(-1)}(t_{\mathrm{L},\widehat{m}}+\Delta^{\widehat{m}}\Phi(s-\gamma h_S))}^{\widehat{m}^{(-1)}(t_{\mathrm{L},\widehat{m}}+\Delta^{\widehat{m}}\Phi(s+\gamma h_S))} \mathfrak{K}_j\left[\frac{\Phi^{-1}\left\{\frac{\widehat{m}(u)-t_{\mathrm{L},\widehat{m}}}{\Delta^{\widehat{m}}}\right\} - s}{h_S}\right]\mathrm{d}u\,.$$

To prove (G.29), first for any $j \in \{0,1,2\}$, the map $u \mapsto |u|^j\phi(u)$ is uniformly bounded and decreasing when $u \geqslant \mathbb{I}(j=1) + \mathbb{I}(j=2)\sqrt{2}$. Since $\gamma > \sqrt{2}$ by definition, we have, uniformly in $s \in \mathbb{R}$,

$$\begin{aligned}|\mathcal{J}_j^{\widehat{m}}(s) - \mathcal{J}_j^{\widehat{m}}(s;\gamma)| \leqslant& \big[b - \widehat{m}^{(-1)}\{t_{\mathrm{L},\widehat{m}} + \Delta^{\widehat{m}}\Phi(s+\gamma h_S)\}\big]|\mathfrak{K}_j(\gamma)| \\ &+ \big[\widehat{m}^{(-1)}\{t_{\mathrm{L},\widehat{m}} + \Delta^{\widehat{m}}\Phi(s-\gamma h_S)\} - a\big]|\mathfrak{K}_j(-\gamma)| \\ \leqslant& \, 2(b-a)\mathfrak{K}_j(\gamma) = O\{\gamma^2\exp(-\gamma^2/2)\}\,. \end{aligned} \tag{G.31}$$

Next we show that, as $n \to \infty$,

$$\sup_{s^2 \leqslant 8\log(nh_S)} \left|S_{n,j}^{\widehat{m}}(s) - \mathcal{J}_j^{\widehat{m}}(s;\gamma)\right| = O_{a.s.}\left\{n^{-1}h_S^{-1}\gamma + n^{-1} + h_S^{-1}\gamma\exp(-\gamma^2/2)\right\}. \tag{G.32}$$

Together, (G.32), (G.31) and a suitable choice for γ prove (G.29).

To show (G.32), consider $\mathcal{C}^{\widehat{m}}(s;\gamma) = \{i : |\Phi^{-1}[\{\widehat{m}(U_i) - t_{\mathrm{L},\widehat{m}}\}/\Delta^{\widehat{m}}] - s| \leqslant \gamma h_S\}$, and observe that $i \in \mathcal{C}^{\widehat{m}}(s;\gamma)$ if and only if

$$U_i \in \Big[\widehat{m}^{(-1)}\Big(t_{\mathrm{L},\widehat{m}} + \Delta^{\widehat{m}}\Phi(s-\gamma h_S)\Big), \widehat{m}^{(-1)}\Big(t_{\mathrm{L},\widehat{m}} + \Delta^{\widehat{m}}\Phi(s+\gamma h_S)\Big)\Big]\,. \tag{G.33}$$

Using again the fact the map $u \mapsto |u|^j\phi(u)$ is uniformly bounded and decreasing when $u \geqslant \mathbb{I}(j=1) + \mathbb{I}(j=2)\sqrt{2}$ (see above (G.31)), we deduce that $U_i \notin \mathcal{C}^{\widehat{m}}(s;\gamma)$ implies that

$$\left|\mathfrak{K}_j\left(\frac{\Phi^{-1}\left\{\frac{\widehat{m}(U_i)-t_{\mathrm{L},\widehat{m}}}{\Delta^{\widehat{m}}}\right\} - s}{h_S}\right)\right| = \mathbb{I}(\gamma < \sqrt{2})O(1) + \mathbb{I}(\gamma > \sqrt{2})O\{\gamma\exp(-\gamma^2/2)\}\,. \tag{G.34}$$

Since $\gamma > \sqrt{2}$ by definition, and since the cardinality of $\mathcal{C}^{\widehat{m}}(s;\gamma)$ is bounded by n, then with $S_{n,j}^{\widehat{m},T}(s;\gamma)$ denoting a version of $S_{n,j}^{\widehat{m}}(s)$ at (G.5) where $\sum_{i=1}^n$ is replaced by $\sum_{i\in\mathcal{C}^{\widehat{m}}(s;\gamma)}$, we deduce from (G.34) that, as $n \to \infty$,

$$\left|S_{n,j}^{\widehat{m}}(s) - S_{n,j}^{\widehat{m},T}(s;\gamma)\right| = O\{h_S^{-1}\gamma\exp(-\gamma^2/2)\}\,. \tag{G.35}$$

In view of (G.35), (G.32) is proved if we show that $S_{n,j}^{\widehat{m},T} - \mathcal{J}_j^{\widehat{m}} = O_{a.s.}(n^{-1}h_S^{-1}\gamma)$ uniformly in $|s| \leqslant \sqrt{8\log(nh_S)}$.

For this, we start by deriving a bound for the cardinality of $\mathcal{C}^{\widehat{m}}(s;\gamma)$ (see (G.37) below) which will be useful later in our calculations. To do so, (G.30) implies, as $n \to \infty$, that $\widehat{m}' \geqslant c_m/2 > 0$ a.s. on $[a,b]$, which implies that $\{\widehat{m}^{(-1)}\}' \leqslant 2c_m^{-1}$ on $[t_{\mathrm{L},\widehat{m}}, t_{\mathrm{U},\widehat{m}}]$. Moreover, the fact that $\widehat{m}$ is a.s. strictly increasing on $[a,b]$ combined with Condition (C2) implies that $\Delta^{\widehat{m}} \overset{a.s.}{=} \widehat{m}(b) - \widehat{m}(a) = O_{a.s.}(1)$ as $n \to \infty$. Using the mean-value theorem, we deduce that, as $n \to \infty$.

$$\widehat{m}^{(-1)}\Big(t_{\mathrm{L},\widehat{m}} + \Delta^{\widehat{m}}\Phi(s+\gamma h_S)\Big) - \widehat{m}^{(-1)}\Big(t_{\mathrm{L},\widehat{m}} + \Delta^{\widehat{m}}\Phi(s-\gamma h_S)\Big) = O_{a.s.}(\gamma h_S) \times \sup_{\alpha\in[-1,1]} \phi(s+\alpha\gamma h_S)\,. \quad \text{(G.36)}$$

In view of (G.36), noting that the definition of the U_i's at the beginning of Section G implies that $U_{i+1} - U_i = (b-a)(n-1)^{-1}$, we deduce that, as $n \to \infty$,

$$\mathrm{Card}\{\mathcal{C}^{\widehat{m}}(s;\gamma)\} \leqslant 1 + O_{a.s.}(n\gamma h_S) \sup_{\alpha\in[-1,1]} \phi(s+\alpha\gamma h_S)\,. \quad \text{(G.37)}$$

Now observe that as $U_{i+1} - U_i = (b-a)n^{-1}\{1 + O(n^{-1})\}^{-1}$, we have

$$S_{n,j}^{\widehat{m},T}(s;\gamma) = (nh_S)^{-1} \sum_{i\in\mathcal{C}^{\widehat{m}}(s;\gamma)} \int_{U_i}^{U_{i+1}} \mathfrak{K}_j\Bigg(\frac{\Phi^{-1}\Big\{\frac{\widehat{m}(U_i)-t_{\mathrm{L},\widehat{m}}}{\Delta^{\widehat{m}}}\Big\} - s}{h_S}\Bigg)(U_{i+1}-U_i)^{-1}\mathrm{d}u$$

$$= \frac{\{1+O(n^{-1})\}}{(b-a)h_S} \sum_{i\in\mathcal{C}^{\widehat{m}}(s;\gamma)} \Bigg[\int_{U_i}^{U_{i+1}} \mathfrak{K}_j\Bigg(\frac{\Phi^{-1}\Big\{\frac{\widehat{m}(u)-t_{\mathrm{L},\widehat{m}}}{\Delta^{\widehat{m}}}\Big\} - s}{h_S}\Bigg)\mathrm{d}u + R_i(s)\Bigg]\,, \quad \text{(G.38)}$$

where we have introduced

$$R_i(s) = \int_{U_i}^{U_{i+1}} \mathfrak{K}_j\Bigg(\frac{\Phi^{-1}\Big\{\frac{\widehat{m}(U_i)-t_{\mathrm{L},\widehat{m}}}{\Delta^{\widehat{m}}}\Big\} - s}{h_S}\Bigg) - \mathfrak{K}_j\Bigg(\frac{\Phi^{-1}\Big\{\frac{\widehat{m}(u)-t_{\mathrm{L},\widehat{m}}}{\Delta^{\widehat{m}}}\Big\} - s}{h_S}\Bigg)\mathrm{d}u\,.$$

Since (G.33) and the fact that $U_{i+1} - U_i = O(n^{-1})$ imply that

$$\min_{i\in\mathcal{C}^{\widehat{m}}(s;\gamma)} U_i - \widehat{m}^{(-1)}\Big(t_{\mathrm{L},\widehat{m}} + \Delta^{\widehat{m}}\Phi(s-\gamma h_S)\Big) = O(n^{-1})\,,$$

$$\max_{i\in\mathcal{C}^{\widehat{m}}(s;\gamma)} U_{i+1} - \widehat{m}^{(-1)}\Big(\widehat{L}(x_{\mathrm{L,NP}}) + \Delta^{\widehat{m}}\Phi(s+\gamma h_S)\Big) = O(n^{-1})\,,$$

we deduce from (G.38) that

$$\big|S_{n,j}^{\widehat{m},T}(s;\gamma) - \mathcal{J}_j^{\widehat{m}}(s;\gamma)\big| \leqslant \frac{\{1+O(n^{-1})\}}{(b-a)h_S}\sum_{i\in\mathcal{C}^{\widehat{m}}(s;\gamma)}|R_i(s)| + O(n^{-1}h_S^{-1})\,. \tag{G.39}$$

Here, we also used the fact that the $\mathfrak{K}_j$'s are bounded. Hence, in view of (G.39), to prove (G.32), it suffices to derive a bound for $\sum_{i\in\mathcal{C}^{\widehat{m}}(s;\gamma)}|R_i(s)|$.

To do this, for all $i\in\mathcal{C}^{\widehat{m}}(s;\gamma)$ such that $i+1\in\mathcal{C}^{\widehat{m}}(s;\gamma)$, we have

$$\Big[\Phi^{(-1)}\Big\{\frac{\widehat{m}(U_i)-t_{\mathrm{L},\widehat{m}}}{\Delta^{\widehat{m}}}\Big\},\Phi^{(-1)}\Big\{\frac{\widehat{m}(U_{i+1})-t_{\mathrm{L},\widehat{m}}}{\Delta^{\widehat{m}}}\Big\}\Big] \subset [s-\gamma h_S, s+\gamma h_S]\,.$$

Hence, since $\|\mathfrak{K}_j'\|_\infty<\infty$, using the mean-value theorem we conclude that for any such i's, we have, for n sufficiently large,

$$\begin{aligned}|R_i(s)| &\leqslant \|\mathfrak{K}_j'\|_\infty h_S^{-1}(\Delta^{\widehat{m}})^{-1}\sup_{x\in\mathcal{D}}|\widehat{m}'(x)|\Big\{\inf_{u\in[s-h_S\gamma,s+h_S\gamma]}\phi(u)\Big\}^{-1}(U_{i+1}-U_i)^2 \quad \text{a.s.}\\ &= O_{a.s.}(n^{-2}h_S^{-1})\times\Big\{\inf_{u\in[s-h_S\gamma,s+h_S\gamma]}\phi(u)\Big\}^{-1},\end{aligned}$$

where, to obtain the second line, we used t Condition (C2), which implies that $(\Delta^{\widehat{m}})^{-1}\sup_{x\in\mathcal{D}}|\widehat{m}(x)| = O_{a.s.}(1)$ a.s. as $n\to\infty$.

Since, for any s, we also have $|R_i(s)|\leqslant\|\mathfrak{K}_j\|_\infty(U_{i+1}-U_i) = \|\mathfrak{K}_j\|_\infty n^{-1}\Delta$, we deduce that

$$|R_i(s)| \leqslant \min\Big[(nh_S)^{-1}\Big\{\inf_{u\in[s-h_S\gamma,s+h_S\gamma]}\phi(u)\Big\}^{-1},1\Big]O_{a.s.}(n^{-1})\,.$$

For the unique value of $i\in\mathcal{C}^{\widehat{m}}(s;\gamma)$ such that $i+1\notin\mathcal{C}^{\widehat{m}}(s;\gamma)$ (this value is equal to $\max\{\mathcal{C}^{\widehat{m}}(s;\gamma)\}$), we can use the bound $|R_i(s)|\leqslant(U_{i+1}-U_i)|\mathfrak{K}_j|\lesssim n^{-1}$. Therefore, using the last displayed equation, we deduce that

$$\begin{aligned}|\sum_{i\in\mathcal{C}^{\widehat{m}}(s;\gamma)}R_i(s)| &\leqslant h_S^{-1}\Delta\mathrm{Card}(\mathcal{C}^L(s;\gamma))n^{-2}\Big\{\inf_{u\in[s-h_S\gamma,s+h_S\gamma]}\phi(u)\Big\}^{-1}+n^{-1}\\ &\lesssim n^{-1}\gamma\Big\{\sup_{\alpha\in[-1,1]}\phi(s+\alpha\gamma h_S)\Big\}\Big\{\inf_{u\in[s-h_S\gamma,s+h_S\gamma]}\phi(u)\Big\}^{-1}+n^{-1}\,,\end{aligned}$$

$$
\begin{aligned}
&|\sum_{i\in\mathcal{C}^{\widehat{m}}(s;\gamma)} R_i(s)| \\
&\qquad \leqslant \mathrm{Card}(\mathcal{C}^L(s;\gamma)) \min\big[(nh_S)^{-1}\big\{\inf_{u\in[s-h_S\gamma,s+h_S\gamma]}\phi(u)\big\}^{-1},1\big]O_{a.s.}(n^{-1})+n^{-1} \\
&\qquad = \Big\{1+O_{a.s.}(n\gamma h_S)\sup_{\alpha\in[-1,1]}\phi(s+\alpha\gamma h_S)\Big\} \\
&\qquad\quad \times \min\big[(nh_S)^{-1}\big\{\inf_{u\in[s-h_S\gamma,s+h_S\gamma]}\phi(u)\big\}^{-1},1\big]O_{a.s.}(n^{-1})+n^{-1}\,, \qquad \text{(G.40)}
\end{aligned}
$$

where, to obtain the last line, we used (G.37).

Now for any $[a,b]\subset\mathbb{R}$ we have $\phi(u)\leqslant\phi(a)\mathbb{I}(b\leqslant 0)+\phi(b)\mathbb{I}(a\geqslant 0)+\phi(0)\mathbb{I}(0\in[a,b])$ and $\phi(u)\geqslant\phi(b)\mathbb{I}(b\leqslant 0)+\phi(a)\mathbb{I}(a\geqslant 0)+\min\{\phi(a),\phi(b)\}\mathbb{I}(0\in[a,b])$. Consequently, since $|s|\leqslant\sqrt{8\log(nh_S)}$ implies that $|s|\gamma h_S\lesssim 1$ when n is large enough (recall the definition of γ and recall that we have assumed that $h_S\log^2(nh_S)\to 0$ as $n\to\infty$), we have, for any $|s|\leqslant\sqrt{8\log(nh_S)}$ and as $n\to\infty$,

$$
\begin{aligned}
\frac{\sup_{u\in[s-h_S\gamma,s+h_S\gamma]}\phi(u)}{\inf_{u\in[s-h_S\gamma,s+h_S\gamma]}\phi(u)} &\leqslant \frac{\phi(s+h_S\gamma)}{\phi(s-h_S\gamma)}\mathbb{I}(s+h_S\gamma\leqslant 0)+\frac{\phi(s-h_S\gamma)}{\phi(s+h_S\gamma)}\mathbb{I}(s-h_S\gamma\geqslant 0) \\
&\qquad + \frac{\phi(0)}{\phi(2)}\mathbb{I}(0\in[s-\gamma h,s+\gamma h]) \\
&= \exp(-2h_S\gamma s)\mathbb{I}(s+h_S\gamma\leqslant 0)+\exp(2h_S\gamma s)\mathbb{I}(s+h_S\gamma>0) \\
&\qquad + \frac{\phi(0)}{\phi(2)}\mathbb{I}(0\in[s-\gamma h,s+\gamma h]) \\
&= O(1)\,. \qquad \text{(G.41)}
\end{aligned}
$$

To obtain the second line, we used Condition (C4), which implies that $h_S\log(nh_S)\to 0$ as $n\to\infty$, and the fact that $\gamma\in(\sqrt{2},\sqrt{8\log(nh_S)})$, so that $h_S\gamma\leqslant 1$ as $n\to\infty$. Thus, when $0\in[s-h_S\gamma,s+h_S\gamma]$, we have $[s-h_S\gamma,s+h_S\gamma]\subset[-2,2]$ as $n\to\infty$.

Combining (G.41) to (G.42) yields

$$
\begin{aligned}
|\sum_{i\in\mathcal{C}^{\widehat{m}}(s;\gamma)} R_i(s)| &= \Big\{1+O_{a.s.}(n\gamma h_S)\sup_{\alpha\in[-1,1]}\phi(s+\alpha\gamma h_S)\Big\} \\
&\quad \times \min\big[(nh_S)^{-1}\big\{\sup_{\alpha\in[-1,1]}\phi(s+\alpha\gamma h_S)\big\}^{-1},1\big]O_{a.s.}(n^{-1})+n^{-1} \qquad \text{(G.42)}
\end{aligned}
$$

$$
\begin{aligned}
&= O_{a.s.}(n\gamma h_S) \sup_{\alpha\in[-1,1]} \phi(s+\alpha\gamma h_S) \\
&\times \min\Big[(nh_S)^{-1}\Big\{\sup_{\alpha\in[-1,1]} \phi(s+\alpha\gamma h_S)\Big\}^{-1}, 1\Big] O_{a.s.}(n^{-1}) + O_{a.s.}(n^{-1}) \\
&= O_{a.s.}\{(\gamma+1)n^{-1}\}\,. \qquad \text{(G.43)}
\end{aligned}
$$

Using (G.39) and (G.42) we deduce that for any $|s| \leqslant \sqrt{8\log(nh_S)}$, as $n \to \infty$ we have

$$
\Big|S_{n,j}^{\widehat{m},T}(s;\gamma) - \mathcal{J}_j^{\widehat{m}}(s;\gamma)\Big| = O_{a.s.}\{\gamma(nh_S)^{-1}\}\,.
$$

The last equation can be combined with (G.35) to prove (G.32). Combining the last equation to (G.35) proves (G.32).

To complete the proof of (G.29), Condition (C5) implies that $h_S \to 0$ as $n \to \infty$, so that $\log(nh_S) = O\{\log(n)\}$. Thus, by plugging $\gamma = \sqrt{4\log(nh_S)}$ into (G.31) and (G.32), we obtain (G.29), which concludes the proof of the lemma. □

Lemma G.8. *Assume that m is a function satisfying Condition (C1), and that $\widehat{m}$ is an estimator of m that fulfils Conditions (C2) and (C3). If h_S satisfies Condition (C4), then, as $n \to \infty$, we have uniformly in $s \in \mathbb{R}$ that for $j \in \{0,2\}$,*

$$
\big|(b-a)\mathcal{J}_j^{\widehat{m}}(s) - E^{\widehat{m}}(s)\big| = O_{a.s.}\Big(h_S^2\phi(|s|-1)\big[1+s^2+\kappa_n a_n^3\big]\Big)
$$

and that

$$
\big|\mathcal{J}_1^{\widehat{m}}(s)\big| = O_{a.s.}\Big(h_S\phi(s)\big(1+|s|+\kappa_n a_n^2\big) + h_S^2\phi(|s|-1)\big[1+s^2+\kappa_n a_n^3\big]\Big)\,.
$$

Proof of Lemma G.8: Recall the definition of $E^{\widehat{m}}$ in (G.16). Also, define $\widetilde{m}(z) = \widehat{m}^{(-1)}\{\Phi(z)\Delta^{\widehat{m}} + t_{\mathrm{L},\widehat{m}}\}$. Since Lemma G.11 in Section G.3 implies $\inf_{x\in[a,b]} \widehat{m}'(x) \geqslant c_m/2$ a.s. for n sufficiently large, where c_m is as in Condition (C1), we have, for n is sufficiently large and with probability one,

$$
\begin{aligned}
\widetilde{m}'(z) &= \frac{\Delta^{\widehat{m}}\phi(z)}{\widehat{m}'\big[\widehat{m}^{(-1)}\big\{\Phi(z)\Delta^{\widehat{m}} + t_{\mathrm{L},\widehat{m}}\big\}\big]} = E^{\widehat{m}}(s)\,, \\
\widetilde{m}''(z) &= \frac{-\Delta^{\widehat{m}}\phi(z)}{\widehat{m}'\big[\widehat{m}^{(-1)}\big\{\Phi(z)\Delta^{\widehat{m}} + t_{\mathrm{L},\widehat{m}}\big\}\big]}\left(z + \frac{\widehat{m}''\big[\widehat{m}^{(-1)}\big\{\Phi(z)\Delta^{\widehat{m}} + t_{\mathrm{L},\widehat{m}}\big\}\big]}{\widehat{m}'\big[\widehat{m}^{(-1)}\big\{\Phi(z)\Delta^{\widehat{m}} + t_{\mathrm{L},\widehat{m}}\big\}\big]}\widetilde{m}'(z)\right)
\end{aligned}
$$

$$= -\widetilde{m}'(z)\Big(z + \frac{\widehat{m}''\big[\widehat{m}^{(-1)}\big\{\Phi(z)\Delta^{\widehat{m}} + t_{\mathrm{L},\widehat{m}}\big\}\big]}{\widehat{m}'\big[\widehat{m}^{(-1)}\big\{\Phi(z)\Delta^{\widehat{m}} + t_{\mathrm{L},\widehat{m}}\big\}\big]}\widetilde{m}'(z)\Big)\,,$$

where, to obtain the second equality we used the fact that $\phi'(z) = -z\phi(z)$.

Proceeding similarly, we obtain that

$$\begin{aligned}\widetilde{m}'''(z) = &-\widetilde{m}''(z)\Big(z + \frac{\widehat{m}''\big[\widehat{m}^{(-1)}\big\{\Phi(z)\Delta^{\widehat{m}} + t_{\mathrm{L},\widehat{m}}\big\}\big]}{\widehat{m}'\big[\widehat{m}^{(-1)}\big\{\Phi(z)\Delta^{\widehat{m}} + t_{\mathrm{L},\widehat{m}}\big\}\big]}\widetilde{m}'(z)\Big)\\ &-\widetilde{m}'(z)\Bigg\{1 + \frac{\widehat{m}'''\big[\widehat{m}^{(-1)}\big\{\Phi(z)\Delta^{\widehat{m}} + t_{\mathrm{L},\widehat{m}}\big\}\big]}{\widehat{m}'\big[\widehat{m}^{(-1)}\big\{\Phi(z)\Delta^{\widehat{m}} + t_{\mathrm{L},\widehat{m}}\big\}\big]}\{\widetilde{m}'(z)\}^2\\ &\qquad + \frac{\widehat{m}''\big[\widehat{m}^{(-1)}\big\{\Phi(z)\Delta^{\widehat{m}} + t_{\mathrm{L},\widehat{m}}\big\}\big]}{\widehat{m}'\big[\widehat{m}^{(-1)}\big\{\Phi(z)\Delta^{\widehat{m}} + t_{\mathrm{L},\widehat{m}}\big\}\big]}\widetilde{m}''(z)\\ &\qquad - \Big(\frac{\widehat{m}''\big[\widehat{m}^{(-1)}\big\{\Phi(z)\Delta^{\widehat{m}} + t_{\mathrm{L},\widehat{m}}\big\}\big]}{\widehat{m}'\big[\widehat{m}^{(-1)}\big\{\Phi(z)\Delta^{\widehat{m}} + t_{\mathrm{L},\widehat{m}}\big\}\big]}\widetilde{m}'(z)\Big)^2\Bigg\}\,.\end{aligned}$$

For any $z \in \mathbb{R}$, we have that, for n sufficiently large, $\widehat{m}^{(-1)}\big\{\Phi(z)\Delta^{\widehat{m}} + t_{\mathrm{L},\widehat{m}}\big\} \in [a,b]$ a.s. (recall that what have established above that $\widehat{m}'$ is a.s. strictly positive on $[a,b]$, which implies that $\widehat{m}$ is bijective on $[a,b]$). Therefore, using the above equations and the results $\inf_{x\in[a,b]} \widehat{m}'(x) \geqslant c_m/2$ a.s, $\sup_{x\in[a,b]} |\widehat{m}^{(\ell)}(x)| = O_{a.s.}(1 + \kappa_n a_n^{\ell})$ a.s. (see Condition (C3)) and $\Delta^{\widehat{m}} = O_{a.s.}(1)$, we deduce that

$$\begin{aligned}|\widetilde{m}'(z)| &= \phi(z) \times O_{a.s.}(1)\,,\\ |\widetilde{m}''(z)| &= |\widetilde{m}'(z)|\Big\{|z| + (\kappa_n a_n^2 + 1)|\widetilde{m}'(z)|\Big\} \times O_{a.s.}(1) = \phi(z)O_{a.s.}\big(|z| + \kappa_n a_n^2 + 1\big)\,.\end{aligned}$$

Proceeding similarly, we also get

$$\begin{aligned}|\widetilde{m}'''(z)| &= \phi(z)O_{a.s.}\Big\{|z| + (\kappa_n a_n^2 + 1)\phi(z)\Big\}O_{a.s.}\Big\{|z| + (1 + \kappa_n a_n^2)\phi(z)\Big\}\\ &\quad + \phi(z)O_{a.s.}\Big(1 + (1 + \kappa_n a_n^3)\phi^2(s) + (1 + \kappa_n a_n^2)\phi(z)\Big\{|z| + (\kappa_n a_n^2 + 1)\phi(z)\Big\}\Big)\\ &= \phi(z)O_{a.s.}\Big[(1 + \kappa_n a_n^3) + \big\{|z| + (\kappa_n a_n^2 + 1)\big\}^2\Big]\\ &= \phi(z)O_{a.s.}\big[1 + z^2 + \kappa_n a_n^3\big]\,,\end{aligned}$$

where, to obtain the last line, we used $(a+b)^2 = O(a^2+b^2)$ and Condition (C2), which implies that $\kappa_n a_n \to 0$ as $n \to \infty$,so that, as $n \to \infty$ that $\kappa_n a_n^2\}^2 + \kappa_n a_n^3 = O(\kappa_n a_n^3)$.

Using the definition of $\mathcal{J}_j^{\widehat{m}}(s)$, we also have

$$(b-a)\mathcal{J}_j^{\widehat{m}}(s) = \frac{\Delta^{\widehat{m}}}{h_S}\int \mathfrak{K}_j\Big(\frac{z-s}{h_S}\Big)\widetilde{m}'(z)\,\mathrm{d}z = \Delta^{\widehat{m}}\int \mathfrak{K}_j(u)\widetilde{m}'(s+h_S u)\,\mathrm{d}u\,,$$

and using a Taylor expansion with the integral form of the remainder term, we have

$$\begin{aligned}\Big|\widetilde{m}'(s+h_S u)-\widetilde{m}'(s)-\widetilde{m}''(s)h_S u\Big| &\leqslant u^2h_S^2\Big|\int_0^1 \widetilde{m}'''(s+t)(1-tuh)\,\mathrm{d}t\Big|\\ &= u^2h_S^2\sup_{t\in[0,1]}\phi(s+t)O_{a.s.}\{1+(s+t)^2+\kappa_n a_n^3\}\\ &= u^2h_S^2\phi(|s|-1)O_{a.s.}(1+s^2+\kappa_n a_n^3)\,.\end{aligned}$$

We deduce that we have, uniformly in $s\in\mathbb{R}$,

$$\begin{aligned}\Big|(b-a)\mathcal{J}_j^{\widehat{m}}(s)-&\widetilde{m}'(s)\int\mathfrak{K}_j(u)\,\mathrm{d}u - h_S\widetilde{m}''(s)\int\mathfrak{K}_{j+1}(u)\,\mathrm{d}u\Big|\\ &= h_S^2\phi(|s|-1)O_{a.s.}\big[1+s^2+\kappa_n a_n^3\big]\int|\mathfrak{K}_{j+2}(u)|\,\mathrm{d}u\,.\end{aligned}$$

Therefore, the proof follows from the facts that $\int|\mathfrak{K}_{j+2}|<\infty$, $\int\mathfrak{K}_0=\int\mathfrak{K}_2=1$ and $\int\mathfrak{K}_1=0$. □

The next Lemma establishes an almost sure bound for the uniform difference between $\mathcal{J}_j^{\widehat{m}}$ and

$$\widetilde{\mathcal{J}}_j^m(s)\equiv\frac{1}{(b-a)h_S}\int_a^b\mathfrak{K}_j\Bigg(\frac{\Phi^{-1}\Big\{\frac{m(u)-t_{\mathrm{L},\widehat{m}}}{\Delta^{\widehat{m}}}\Big\}-s}{h_S}\Bigg)\mathrm{d}u\,. \tag{G.44}$$

Lemma G.9. *Assume that m is a function satisfying Condition (C1), and that $\widehat{m}$ is an estimator of m that fulfils Condition (C2). Then, if h_S satisfies Condition (C4), as $n\to\infty$ we have that*

$$\sup_{|s|\leqslant\sqrt{8\log(nh_S)}}\left\{\frac{\big|\mathcal{J}_j^{\widehat{m}}(s)-\widetilde{\mathcal{J}}_j^m(s)\big|}{\phi\Big(s\frac{\sqrt{1-h_S^2}}{\sqrt{1+h_S^2}}\Big)}\right\}=O_{a.s.}(\kappa_n a_n+b_n)\,.$$

Proof of Lemma G.9: Using Lemma G.11 in Section G.3, we know that, for n sufficiently large, $\widehat{m}'$ is a.s. bounded below by a strictly positive constant. Therefore,

$$\begin{aligned}\mathcal{J}_j^{\widehat{m}}(s) &= h_S^{-1}\int_a^b \mathfrak{K}_j\Big(\frac{\Phi^{-1}\big\{\frac{\widehat{m}(u)-t_{\mathrm{L},\widehat{m}}}{\Delta^{\widehat{m}}}\big\}-s}{h_S}\Big)\,\mathrm{d}u \\ &= \frac{\Delta^{\widehat{m}}}{h_S}\int \mathfrak{K}_j\Big(\frac{z-s}{h_S}\Big)\frac{\phi(z)}{\widehat{m}'\big[\widehat{m}^{(-1)}\big\{\Phi(z)\Delta^{\widehat{m}}+t_{\mathrm{L},\widehat{m}}\big\}\big]}\,\mathrm{d}u \quad \text{a.s.}\end{aligned}$$

and

$$\widetilde{\mathcal{J}}_j^{m}(s) = \frac{\Delta^{\widehat{m}}}{h_S}\int \mathfrak{K}_j\Big(\frac{z-s}{h_S}\Big)\frac{\phi(z)}{m'\big[m^{(-1)}\big\{\Phi(z)\Delta^{\widehat{m}}+t_{\mathrm{L},\widehat{m}}\big\}\big]}\,\mathrm{d}u\,.$$

From there, we deduce that

$$|\mathcal{J}_j^{\widehat{m}}(s)-\widetilde{\mathcal{J}}_j^{m}(s)| \leqslant A_0(s)\sup_{z\in\mathbb{R}}|A_1(z)|\,, \tag{G.45}$$

where

$$\begin{aligned}A_0(s) &= h_S^{-1}\int\Big|\mathfrak{K}_j\Big(\frac{z-s}{h_S}\Big)\phi(z)\Big|\,\mathrm{d}z\,,\\ A_1(z) &= \frac{\Delta^{\widehat{m}}}{\widehat{m}'\big[\widehat{m}^{(-1)}\big\{\Phi(z)\Delta^{\widehat{m}}+t_{\mathrm{L},\widehat{m}}\big\}\big]}-\frac{\Delta^{\widehat{m}}}{m'\big[m^{(-1)}\big\{\Phi(z)\Delta^{\widehat{m}}+t_{\mathrm{L},\widehat{m}}\big\}\big]}\,.\end{aligned}$$

From (G.45), to prove the lemma, we derive a bound for A_0 and A_1.

For A_0, as $n\to\infty$, we have, for any $s^2 \leqslant 8\log(nh_S)$,

$$\begin{aligned}A_0(s) &= \int |u|^j\phi(u)\phi(s+h_Su)\,\mathrm{d}u\\ &= (2\pi)^{-1}\exp\Big\{-s^2\frac{1-h_S^2}{2(1+h_S^2)}\Big\}\int |u|^j\exp\Big\{-\Big(\frac{1+h_S^2}{2}\Big)\Big(u+\frac{sh_S}{1+h_S^2}\Big)^2\Big\}\,\mathrm{d}u\\ &= \phi\Big(s\frac{\sqrt{1-h_S^2}}{\sqrt{1+h_S^2}}\Big)(1+h_S^2)^{-1/2}\int |u-\frac{sh_S}{1+h_S^2}|^j\phi(u)\,\mathrm{d}u\\ &= \phi\Big(s\frac{\sqrt{1-h_S^2}}{\sqrt{1+h_S^2}}\Big)\times O(1)\,.\end{aligned}$$

To obtain the last line, we used the fact $h_S\to 0$ as $n\to\infty$, which implies that $h_S\in(0,1/2)$ for n sufficiently large; we also used Condition (C4), which implies that $h_S\log(n)\to 0$ as $n\to\infty$, so that, as $n\to\infty$, $|h_Ss|\leqslant 1$ uniformly in $s^2\leqslant 8\log(nh_S)$.

To bound A_1, since $\Phi(z) \in [0,1]$, we have $\Phi(z)\Delta^{\widehat{m}} + t_{\mathrm{L},\widehat{m}} \in [t_{\mathrm{L},\widehat{m}}, t_{\mathrm{U},\widehat{m}}]$, which implies that

$$\begin{aligned}\sup_{z\in\mathbb{R}} |A_1(z)| &= \Delta^{\widehat{m}} \sup_{t\in[t_{\mathrm{L},\widehat{m}},t_{\mathrm{U},\widehat{m}}]} \left|\frac{1}{\widehat{m}'\{\widehat{m}^{(-1)}(z)\}} - \frac{1}{m'\{m^{(-1)}(z)\}}\right| \\ &= \Delta^{\widehat{m}} \sup_{t\in[t_{\mathrm{L},\widehat{m}},t_{\mathrm{U},\widehat{m}}]} \left|\{\widehat{m}^{(-1)}\}'(t) - [m^{(-1)}]'(t)\right| .\end{aligned}$$

To obtain the last line, we used Lemma G.11 in Section G.3, which implies that when n is large enough, $\inf_{x\in[a,b]} \widehat{m}' \geqslant c_m/2$ a.s., with c_m is as in Condition (C1); this implies that $\widehat{m}^{(-1)}$ is a.s. differentiable on $[t_{\mathrm{L},\widehat{m}}, t_{\mathrm{U},\widehat{m}}]$.

From there, since Condition (C2) implies that $\Delta^{\widehat{m}} = O_{a.s.}(1)$, we deduce from Lemma G.11 in Section G.3 that, as $n \to \infty$,

$$\sup_{z\in\mathbb{R}} |A_1(z)| = O_{a.s.}(\kappa_n a_n + b_n) .$$

We complete the proof by combining the bounds for A_0 and A_1. □

The proof of the following lemma is very similar that of Lemma G.8 and hence is omitted.

Lemma G.10. *Assume that m is a function satisfying Condition (C1), and that $\widehat{m}$ is an estimator of m that fulfils Condition (C2). If h_S satisfies Condition (C4), then, as $n \to \infty$, we have uniformly in $s \in \mathbb{R}$ that for $j \in \{0,2\}$,*

$$\left|(b-a)\widetilde{\mathcal{J}}_j^m(s) - \widetilde{E}^m(s)\right| = \phi(|s|-1)(1+s^2)O(h_S^2)$$

and that

$$\left|\mathcal{J}_1^{\widehat{m}}(s)\right| = O\Big(h_S\phi(s)(1+|s|) + h_S^2\phi(|s|-1)(1+s^2)\Big) .$$

G.3 Auxiliary Results

The following result establishes useful large sample almost sure inequalities satisfied by uniformly convergent estimators of strictly increasing functions.

Lemma G.11. *Assume that m is a function satisfying Condition (C1), and that $\widehat{m}$ is an estimator of m that fulfils Condition (C2). Then, as $n \to \infty$ we have*

$$\inf_{x \in [a,b]} \widehat{m}'(x) \geqslant c_m/2 \quad a.s., \tag{G.46}$$

where c_m is as in Condition (C1). Furthermore, as $n \to \infty$ we have

$$\sup_{z \in [t_{L,\widehat{m}}, t_{U,\widehat{m}}]} |\{\widehat{m}^{(-1)}(z)\}' - \{m^{(-1)}(z)\}'| = O_{a.s.}(\kappa_n a_n + b_n)\,. \tag{G.47}$$

Proof of Lemma G.11: To prove (G.46), note from Condition (C1) that there exists a constant $c_m > 0$ such that $\inf_{x \in \widetilde{\mathcal{I}}} m'(x) \geqslant c_m$, where $\widetilde{\mathcal{I}}$ is an open interval that contains $[a, b]$. Moreover Conditions (C2) and (C4) imply that we have, uniformly on $[a, b]$, $\widehat{m}' \to m'$ a.s., so that $\sup_{x \in [a,b]} |\widehat{m}'(x) - m'(x)| \leqslant c_m/2$ a.s. n sufficiently large. We deduce that, for sufficiently large n, we have with probability one that

$$\inf_{x \in [a,b]} \widehat{m}'(x) \geqslant \inf_{x \in [a,b]} m'(x) - \sup_{x \in \mathcal{D}} |\widehat{m}'(x) - m'(x)| \geqslant c_m/2\,, \tag{G.48}$$

which proves (G.46).

Now to prove (G.47), note when (G.48) holds, the derivative of $\widehat{m}$ is strictly positive on $[a, b]$, which implies that $\widehat{m}$ is strictly increasing on $[a, b]$. It also implies that its inverse is strictly increasing and differentiable. This implies that for n large enough, with probability one we have $[t_{\mathrm{L},\widehat{m}}, t_{\mathrm{U},\widehat{m}}] = [\widehat{m}(a), \widehat{m}(b)]$ and

$$\begin{aligned}\sup_{z \in [t_{\mathrm{L},\widehat{m}}, t_{\mathrm{U},\widehat{m}}]} |\widehat{m}^{(-1)}(z) - m^{(-1)}(z)| &= \sup_{u \in [a,b]} |z - m^{(-1)}\{\widehat{m}(u)\}| \\ &= \sup_{u \in [a,b]} \left| m^{(-1)}\{m(u)\} - m^{(-1)}\{\widehat{m}(u)\} \right|.\end{aligned}$$

Using the mean value theorem, there exists $t_u^\star$ between $\widehat{m}(u)$ and $m(u)$ so that

$$m^{(-1)}\{m(u)\} - m^{(-1)}\{\widehat{m}(u)\} = \frac{1}{m'\{m^{(-1)}(t_u^\star)\}}\{m(u) - \widehat{m}(u)\}\,.$$

Moreover, for any $u \in [a, b]$, the equality $\widehat{m}(u) = m(u) + o_{a.s.}(1)$ and the continuity of $m^{(-1)}$ imply that, for n sufficiently large, we have $m^{(-1)}(t_u^\star) \in \widetilde{\mathcal{I}}$ a.s. Therefore, using

the inequality $\inf_{x\in\widetilde{\mathcal{I}}} m'(x) \geqslant c_m$ (see Condition (C1)) and the above equations, we deduce that, as $n \to \infty$,

$$\sup_{z\in[t_{\mathrm{L},\widehat{m}},t_{\mathrm{U},\widehat{m}}]} |\widehat{m}^{(-1)}(z)-m^{(-1)}(z)| = \sup_{u\in[a,b]} \left|m^{(-1)}\{m(u)\}-m^{(-1)}\{\widehat{m}(u)\}\right| = O_{a.s.}(\kappa_n+b_n)\,, \tag{G.49}$$

where we used Condition (C2), which implies that $\sup_{u\in[a,b]} |m(u)-\widehat{m}(u)| = O_{a.s.}(\kappa_n+b_n)$.

Noting that for any strictly increasing and continuously differentiable functions g_1 and g_2, we have $[g_1^{(-1)} - g_2^{(-1)}]' = (g_2' \circ g_2^{(-1)} - g_1' \circ g_1^{(-1)})/(g_1' \circ g_1^{(-1)} g_2' \circ g_2^{(-1)})$, we deduce that

$$\sup_{z\in[t_{\mathrm{L},\widehat{m}},t_{\mathrm{U},\widehat{m}}]} |[\widehat{m}^{(-1)}(z)]' - [m^{(-1)}(z)]'| \leqslant \Theta_n^{(1)} \times \left\{\Theta_n^{(2)} \times \Theta_n^{(3)}\right\}^{-1}, \tag{G.50}$$

where we have used the notations

$$\Theta_n^{(1)} = \sup_{u\in[a,b]} |\widehat{m}'(u) - m'[m^{(-1)}\{\widehat{m}(u)\}]|\,,$$
$$\Theta_n^{(2)} = \inf_{x\in[a,b]} |\widehat{m}'(x)|\,, \qquad \text{and} \qquad \Theta_n^{(3)} = \inf_{z\in[t_{\mathrm{L},\widehat{m}},t_{\mathrm{U},\widehat{m}}]} m'\{m^{(-1)}(z)\}\,.$$

To bound $\Theta_n^{(1)}$, using Condition (C2) and (G.49), as $n \to \infty$ we have

$$\Theta_n^{(1)} \leqslant \sup_{u\in[a,b]} |\widehat{m}'(u) - m(u)| + \sup_{u\in[a,b]} |m^{(-1)}\{\widehat{m}(u)\} - m^{(-1)}\{m(u)\}| = O_{a.s.}(\kappa_n a_n + b_n)\,,$$

where we also used the fact that $O_{a.s.}\{\kappa_n(a_n+1)\} = O_{a.s.}(\kappa_n a_n)$ (see Condition (C2)).

Next, that $\{\Theta_n^{(2)}\}^{-1} = O_{a.s.}(1)$ follows from (G.46) and Condition (C1). Hence, to show (G.47), it remains to prove that $\{\Theta_n^{(3)}\}^{-1} = O_{a.s}(1)$.

To do this, since L is strictly increasing and continuous and $\widetilde{\mathcal{I}}$ is an open set that contains $[a,b]$ (see Condition (C2)), there exists a constant $\epsilon > 0$ such that $[m^{(-1)}\{L(a)-\epsilon\}, m^{(-1)}\{L(b)+\epsilon\}] \subset \widetilde{\mathcal{I}}$. Moreover, Condition (C2) implies that $\widehat{m}(a) = L(a) + o_{a.s.}(1)$ and $\widehat{m}(b) = L(a) + o_{a.s.}(1)$, which implies that $\widehat{m}(a) \geqslant L(a) - \epsilon$ a.s. and $\widehat{m}(b) \leqslant L(b) + \epsilon$ a.s., as $n \to \infty$. Since we have also proved above that

$(t_{\mathrm{L},\widehat{m}}, t_{\mathrm{U},\widehat{m}}) = (\widehat{m}(a), \widehat{m}(b))$ a.s., we deduce that, as $n \to \infty$,

$$\{\Theta_n^{(3)}\}^{-1} \leqslant \Big\{ \inf_{x \in \widetilde{\mathcal{I}}} m'(x) \Big\}^{-1} \leqslant c_m^{-1} \quad \text{a.s.}, \tag{G.51}$$

where, to obtain the last inequality, we used Condition (C1). This concludes the proof of (G.47) and of the lemma. □

The following result states the analytic expression for the solution of the local the log-quadratic likelihood maximization program under a Gaussian kernel. It can be proved using long but relatively straightforward algebraic manipulations.

Lemma G.12. *Let $V_1, \ldots, V_n$ be real numbers, ϕ be the standard normal density, $h_S > 0$ and $S_{n,j}(s) = (nh_S)^{-1} \sum_{i=1}^n \phi\{(V_i - s)/h_S\}\{(V_i - s)/h_S\}^j$. Set*

$$\begin{aligned}\Upsilon_s(a_0, a_1, a_2) = &\sum_{i=1}^n \phi\Big(\frac{V_i - s}{h_S}\Big)\big\{a_0 + a_1(V_i - s) + a_2(V_i - s)^2\big\} \\ &- n \int \phi\Big(\frac{t - s}{h_S}\Big) \exp\{a_0 + a_1(t - s) + a_2(t - s)^2\}\, dt\,.\end{aligned}$$

Then, for any s such that $S_{n,2}(s)S_{n,0}(s) - S_{n,1}^2(s) \neq 0$, we have, for $(\widehat{a}_0, \widehat{a}_1, \widehat{a}_2)$ such that $\nabla\Upsilon_s(\widehat{a}_0, \widehat{a}_1, \widehat{a}_2) = 0$,

$$\exp(\widehat{a}_0) = S_{n,0}(s)\Big\{\frac{S_{n,0}^2(s)}{S_{n,2}(s)S_{n,0}(s) - S_{n,1}^2(s)}\Big\}^{-1/2} \exp\Big\{\frac{-S_{n,1}^2(s)/2}{S_{n,2}(s)S_{n,0}(s) - S_{n,1}^2(s)}\Big\}\,.$$

References

Camirand Lemyre, F., Carroll, R.J. and Delaigle, A. (2022). Semiparametric estimation of the distribution of episodically consumed foods measured with error. *J. Am. Stat. Assoc.*, **117**, 469–481.

Chang, J., Delaigle, A., Hall, P. and Tang, C-Y. (2018). A frequency domain analysis of the error distribution from noisy high-frequency data. *Biometrika*, **105**, 353–369.

Delaigle, A. (2014). Nonparametric kernel methods with errors-in-variables: constructing estimators, computing them, and avoiding common mistakes. *Australian & New Zealand J. Statist.*, **56**, 105–124.

Dette, H., Neumeyer, N., Pilz, K. F. (2006). A simple nonparametric estimator of a strictly monotone regression function. *Bernoulli*, **12**, 469–490.

Fan, J. (1991). Asymptotic normality for deconvolution kernel density estimators. *Sankhya*, Series A, **53**, 97–110.

Feller, W. (1968). *An Introduction to Probability Theory and Its Applications* (third ed.), Volume 1. New York: Wiley.

Geenens, G. (2014). Probit transformation for kernel density estimation on the unit interval. *J. Am. Stat. Assoc.*, **109**, 346–358.

Loader, C.R. (1996). Local likelihood density estimation, *Ann. Statist.*, **24**, 1602–1618.

Masry, E. (1991). Multivariate probability density deconvolution for stationary random processes. *IEEE Transactions on Information Theory*, **37**, 1105–1115.

Masry, E. (1993). Strong consistency and rates for deconvolution of multivariate densities of stationary processes. *Stochastic Process. Appl.*, **47**, 53–74.

Meister A. (2009). *Deconvolution Problems in Nonparametric Statistics.* Springer.

Tooze, J.A., Kipnis, V., Buckman, D.W., Carroll, R.J., Freedman, L.S., Guenther, P.M., Krebs-Smith, S.M., Subar, A.F. and Dodd, K.W. (2010). A mixed-effects model approach for estimating the distribution of usual intake of nutrients: the NCI method. *Stat. Med.*, **29**, 2857–2868.

Vretblad, Anders. (2003). *Fourier Analysis and its Applications*, Springer.

Yukich, J. (1987). Some limit theorems for the empirical process indexed by functions. *Probab. Theory Rel.*, **74**, 71–90.
