# Supplementary material for: Nonparametric Density Estimation of a Long-Term Trend from Repeated Semicontinuous Data
Source: J Am Stat Assoc. Author manuscript; Available in PMC 2026 Jun 3. (PMC13229538; doi:10.1080/01621459.2025.2555054)
Supplement: Supp 1 [file NIHMS2120148-supplement-Supp_1.zip › ETASDataDictionary.pdf]

Data Dictionary

The data provided in the file `EATS_Data.txt` contain the data values used in our analysis in Section 7. They are derived from the Eating at America's Table Study described in: Subar, A.F., Thompson, F.E., Kipnis, V., Midthune, D., Hurwitz, P., McNutt, S., McIntosh, A. and Rosenfeld, S. (2001). Comparative validation of the Block, Willett and National Cancer Institute food frequency questionnaires: The Eating at America's Table Study. *Am. J. Epid.*, **154**, 1089–1099.

File Structure

- The file contains **965 rows** and **4 columns**.
- Each row corresponds to one individual.
- Each column corresponds to one of four **24-hour recalls**.
- Values in each row are separated by a comma (',').
- For $i = 1, \ldots, 965$ and $j = 1, \ldots, 4$, the value at the ith row and jth column is denoted by $W_{i,j}$, representing the amount of total fruit (in servings) reported by the ith individual during the jth 24-hour dietary recall, divided by their total energy intake (in 1000 kilocalories).
- Fruit consumption amounts below 0.02 servings were truncated to 0 before division by energy intake.

Variable Description

**Column**	**Description**	**Units**	**Type**	**Possible values**
`1`	Amount of total fruit consumed, divided by energy intake, at the first 24-hour dietary recall	Servings per 1000 kilocalories	Continuous	≥ 0
`2`	Same as Column 1, at the second 24-hour dietary recall	Same	Same	Same
`3`	Same as Column 1, at the third 24-hour dietary recall	Same	Same	Same
`4`	Same as Column 1, at the fourth 24-hour dietary recall	Same	Same	Same
