# Supplementary material for: Nonparametric Density Estimation of a Long-Term Trend from Repeated Semicontinuous Data
Source: J Am Stat Assoc. Author manuscript; Available in PMC 2026 Jun 3. (PMC13229538; doi:10.1080/01621459.2025.2555054)
Supplement: Supp 1 [file NIHMS2120148-supplement-Supp_1.zip › SubmittedCode/ReadmeV3.html]

Replication flow for the paper ‘Nonparametric density estimation of a long-term trend from repeated semicontinuous data’


# Replication flow for the paper ‘Nonparametric density estimation of a long-term trend from repeated semicontinuous data’

#### Aurore Delaigle

## 1 Runnning the code on a new dataset

In this section we explain how to run our codes on a new dataset.

### 1.1 Unzip our provided code

Unzip the folder \(\small\verb+Functions+\) of our provided
code. It contains the functions used to compute the estimators
considered in the paper:

- “ComputeEstimatorsfTExample.m” is the main script that performs the
  estimator computations and calls the supporting routines.
- The other provided MATLAB routines are invoked by
  “ComputeEstimatorsfTExample.m”.
- The provided R script is called from within Matlab

### 1.2 Get additional Matlab files from the web:

Download the following Matlab files from the web:

- The function \(\small\verb+bwsjpiSM+\) and its
  dependencies (\(\small\verb+bwosSM+\),\(\small\verb+bwosSM+\),\(\small\verb+lbinrSM+\) and \(\small\verb+rootfSM+\)) from Steve Marron’s
  Smoothing Matlab package available at https://github.com/jsmarron/MarronMatlabSoftware/tree/master/Matlab9/Smoothing/
- The \(\small\verb+outerop+\)
  function at https://au.mathworks.com/matlabcentral/fileexchange/8370-outer-operation?focused=5065519&tab=function

Put those files in the folder \(\small\verb+Functions+\).

### 1.3 Install required R packages

Install the following R packages: “ks”, “MASS”, “locfit”,
“lpdensity”, “DescTools”

### 1.4 Inputs required by the function “ComputeEstimatorsfTExample.m”

- `W`: an \(n \times J\)
  matrix where the \((i,j)\)-th entry
  contains the transformed data value \(h(W\_{ij}) = \widetilde{W}\_{ij}\) as defined
  in the paper, for non-zero components, and 0 for zero components.
- `n`: sample size (number of individuals)
- `nrep`: number \(J\) of
  replicates per individual
- `gridt`: grid of \(t\)
  values at which you wish to estimate \(f\_T(t)\)
- `namedirFct`: Path to the folder where you have unzipped
  the folder \(\small\verb+Functions+\).
  Example: `"C:\Users\yourname\Functions\"`
- `subfolder`: a string specifying the name of a subfolder
  to be created by the code within the \(\small\verb+Functions+\) directory. This
  subfolder is used by the code to store R outputs required for the
  computations. Example `subfolder=['Example1']`.  Important: never run multiple instances of the code
  using the same subfolder name simultaneously, as some files will be
  overwritten. If you need to run several instances of the code at the
  same time, ensure that each uses a unique subfolder name.
- `RscriptPath`: Full path to the Rscript executable on
  your computer. Example:
  `"C:\Program Files\R\R-4.4.0\bin\Rscript"`
- `Htype` (optional): ‘Fnorm’ (for cdf of a normal) or
  ‘Logist’ (for logistic function): parametric form for the function \(H\), which is used for both the parametric
  and semiparametric estimators. This same parametric form is also
  employed when computing the nonparametric estimator of \(H\) in the tails. For the nonparametric
  estimator, the choice of parametric form has little influence on the
  results. The default option is ‘Logist’.
- `KernType` (optional): Kernel to use for deconvolution
  estimator. Don’t change the default unless you’re an expert in
  deconvolution methods.

### 1.5 Outputs of the function “ComputeEstimatorsfTExample.m”

- fThatNPfinal: nonparametric estimator of \(f\_T(t)\) for \(t\) in \(\small\verb+gridt+\).
- fTSP: semiparametric estimator of of \(f\_T(t)\) for \(t\) in \(\small\verb+gridt+\) using the
  semiparametric estimator of \(H\) from
  Camirand Lemyre, Carroll and Delaigle (2022).
- fThatPML: fully parametric ML estimator of \(f\_T(t)\) for \(t\) in \(\small\verb+gridt+\).

### 1.6 Worked out example

A worked example is provided in the file “Example.m” in the folder
\(\verb+SyntheticExample+\), which uses
the supplied data file. The code requires both MATLAB and R to run.

- “DataExample.txt”: contains a synthetic data sample designed to
  replicate the main characteristics of the EATS dataset.
- “Example.m”: runs the code using this example data. It demonstrates
  how to set the required variables, calls “ComputeEstimatorsfTExample.m”
  to perform the computations, and plots the estimated densities.

## 2. Reproducing our simulations

Start by steps 1.1 to 1.3 above. Then follow the following steps.

### 2.0 Read this to prevent errors

To reproduce our simulations, our MATLAB code exchanges data with R
through TXT files that are automatically created during execution in a
subfolder named `subfolder` created during execution within
the \(\small\verb+Functions+\)
directory. Running multiple instances of the code simultaneously
**using the same `subfolder` name** will cause
crucial files to be overwritten, leading to errors.

A unique `subfolder` name is already defined in each of
the 16 simulation Matlab files to prevent conflicts, so that you can run
the 16 different files simultaneously if you wish.

However, if you run multiple instances of
the **same** MATLAB file at the same time, they will share
the same `subfolder`, and files will be overwritten
incorrectly. Therefore, if you need to run multiple instances of the
same simulation Matlab file concurrently, **ensure each instance
uses a unique `subfolder` name**.

### 2.1 Unzip our provided simulation code

Unzip the folder \(\small\verb+Simulations+\) from our
provided code. It contains:

#### (a) MATLAB Simulation Files

12 Matlab files that reproduce the simulations for the 12
combinations of:

- **Density \(f\_X\)**:
  - `XN0` (normal density)
  - `XmixN1` (mixture of two normals)
  - `XmixG1` (mixture of two Gammas)
- **Function \(H\)**:
  - `HLogist` (logistic case)
  - `Hcomplex` (more complex \(H\) example from the paper)
- **Density \(f\_U\)**:
  - `Unorm` (normal density)
  - `ULap` (Laplace density)

The MATLAB files are named following the pattern:
“namefXnamefUnameH.m”. For example “XN0ULapHLogist.m”.

In addition, this folder contains 4 Matlab files for reproducing the
simulation results for the non monotone examples:
“XmixG1ULapHNonMono.m”, “XmixN1ULapHNonMono.m”, “XmixN1ULapHNonMonoV2.m”
and “,”XmixN0ULapHNonMono.m”, which respectively reproduce the
simulation results for all settings, with Laplace error density, of
models (1) and (2) with \(H=H\_3\), (2)
and (3) with \(H=H\_4\).

Each Matlab file runs 1000 simulations for the corresponding setting,
for each combination of:

- **Sample size**: \(n = 250,
  500, 1000\)
- **Number of replicates** (called `nrep` in
  the code, \(J\) in the paper): \(J = 2\) or \(4\)
- **Variance of \(U\)**:
  \(10\%\ \text{var}(X)\) or \(20\%\ \text{var}(X)\)

---

#### (b) R files to reproduce figures and tables of Section 6 (monotone cases)

For each combination of densities \(f\_X\) and \(f\_U\), we provide

- **One R file** for reproducing the numbers shown in the
  tables of the paper for both \(H\)
  functions considered in Section 6 of the paper.
- **One R file** for producing a PDF figure showing the
  true density \(f\_T\) and the quartile
  curves of the estimated densities, for each combination of sample size
  \(n\), number of replicates \(J\), variance of \(U\), function \(H\), estimation method.

**Naming conventions:**

- R files for generating the tables:
  `TableResultsnamefXnamefU.R`  
  Example: `TableResultsXN0ULap.R`
- R files for producing the PDF figures:
  `pdfPlotDensitynamefXnamefU.R`  
  Example: `pdfPlotDensityXN0ULap.R`

In all R files you need to replace \(\verb+C:/YourPath/+\) by the path
`namedir` defined as in Section 2.2 below.

#### (c) R files to reproduce figures and tables of non monotone cases

We also provide 3 R files that can be used to reproduce the numbers
shown in the tables of Appendix B:

- `TableResultsXmixGULapNonMono.R`
- `TableResultsXmixN1ULapNonMono.R`
- `TableResultsXmixN0ULapNonMono.R`

These files respectively reproduce the tables for models (1) with
\(H=H\_3\), (2) with \(H=H\_3\) and with \(H=H\_4\), (3) with \(H=H\_4\).

In that folder, we also provide 3 R files to produce PDF figures
showing the true density \(f\_T\) and
the quartile curves of the estimated densities for each combination of
sample size \(n\), number of replicates
\(J\), variance of \(U\), and estimation method:

- `pdfPlotDensityXmixG1ULapNonMono.R`
- `pdfPlotDensityXmixN1ULapNonMono.R`
- `pdfPlotDensityXmixN0ULapNonMono.R`

These files respectively produce the figures for models (1) with
\(H=H\_3\), (2) with \(H=H\_3\) and with \(H=H\_4\), (3) with \(H=H\_4\).

In all R files you need to replace \(\verb+C:/YourPath/+\) by the path
`namedir` defined as in Section 2.2 below.

---

### 2.2 Inputs for Matlab simulation files at 2.1 (a)

To run the 16 Matlab files, you need to provide the following inputs
within each of them, at the designated input sections already included
in those files:

- `namedirFct`: Path to the folder where you have unzipped
  the folder \(\small\verb+Functions+\).
  Example: `"C:\Users\yourname\Functions\"`
- `RscriptPath`: Full path to the Rscript executable on
  your computer. Example:
  `"C:\Program Files\R\R-4.4.0\bin\Rscript"`
- `namedir`: For our simulations, the code writes outputs
  to TXT files. The variable `namedir` specifies the path to
  the directory where these output files will be saved. Example
  `C:\Users\yourname\Results\`

### 2.3 Outputs from Matlab simulation files at 2.1 (a)

For our simulations, the Matlab code writes output to TXT files in
the folder specified by the input variable `namedir`. The
names of the output TXT files are generated automatically by the code as
described below. For each of the 16 settings, the codes writes the
following outputs to separate TXT files:

- The grid \(\small\verb+gridt+\)
  of points at which the estimators and the true density \(f\_T\) were evaluated. Each gridt file
  contains one row for each combination of sample size and error
  variance.
- the nonparametric, semiparametric and parametric ML estimators of
  \(f\_T(t)\) for \(t\) in \(\small\verb+gridt+\), for each generated
  samples. For each value of `nrep` (i.e., 2 and 4), there is
  one output file per estimation method. Within each file, each line
  corresponds to the estimated density for one sample.
- The integrated squared error (ISE) of the above estimators, for
  each generated samples. For each value of `nrep` (i.e., 2 and
  4), there is one output file per estimation method.

**Naming conventions:**

The names of the output files are constructed by combining the
following components, in this order:

- Name of the distribution of \(X\):
  - \(\small\verb+XNorn0B+\) for the
    normal density,
  - \(\small\verb+XmixNorm1+\) for the
    mixture of two normals,
  - \(\small\verb+XmixGamma+\) for the
    mixture of two Gammas.
- Name of the distribution of \(U\):
  \(\small\verb+Unorm+\) (normal) or
  \(\small\verb+ULap+\) (Laplace)
- Type of function \(H\): \(\small\verb+HLogist+\) (logistic case),
  \(\small\verb+HassumeLogist+\) (more
  complex \(H\) example from the paper)
  or \(\small\verb+HNonMonoassumeLogist+\) and
  \(\small\verb+HNonMonoV2assumeLogist+\)
  (non monotone examples, following names explained in Section 2.1
  above)
- Number \(J\) of replicates: (\(\small\verb+rep2+\) for \(J=2\) or \(\small\verb+rep4+\) for \(J=4\))
- File content type:
  - \(\small\verb+Gridt+\) for a file
    storing the \(\small\verb+gridt+\)
    values,
  - \(\small\verb+TruefT+\) for a file
    storing the true \(f\_T\),
  - \(\small\verb+ISE+\) for a file
    storing the Integrated Squared Errors,
  - \(\small\verb+fT+\) for a file
    storing the estimators
- Name of method (used only for estimator files , not for $ and \(\small\verb+TruefT+\) files):
  - \(\small\verb+NPfinal+\) for the
    proposed nonparametric estimator,
  - \(\small\verb+PML+\) or the maximum
    likelihood estimat
  - \(\small\verb+SP+\) for the
    semiparametric estimator with an incorrectly specified model for \(H\)
  - \(\small\verb+SPC+\) for the
    semiparametric estimator with a correctly specified model for \(H\).

### 2.4 Outputs from R codes for figures and tables, described at 2.1

Outputs from 2.1 (b) are as follows (outputs follow similar
convention for 2.1 (c), but with obvious name changes, such as
`NonMono` added).

#### (a) Table TXT Files

The R scripts generating the simulation tables produce **6 TXT
files**, one for each combination of \(f\_X\) and \(f\_U\).

**Naming convention:**

TXT files produced with tables contain the name of the densities
\(f\_X\) and \(f\_U\) in the following pattern:
“TablenamefXnamefU.txt”, for example “TableXNorm0ULap.txt”.

#### (b) Figure pdf Files

The R scripts generating the estimated density figures produce one
PDF file per estimator per setting. File names include the following
components (in this order):

- Name of the distribution of \(X\):
  - \(\small\verb+XN0B+\) for the
    normal density,
  - \(\small\verb+XN1+\) for the
    mixture of two normals,
  - \(\small\verb+XG1+\) for the
    mixture of two Gammas.
- Name of the distribution of \(U\):
  \(\small\verb+Unorm+\) (normal) or
  \(\small\verb+ULap+\) (Laplace)
- Type of function \(H\): \(\small\verb+H1+\) (logistic case), \(\small\verb+H2+\) (more complex \(H\) example from the paper) or \(\small\verb+HNM+\) and \(\small\verb+HNMV2+\) (for the non monotone
  examples in Appendix B, following names explained in Section 2.1
  above)
- Number \(J\) of replicates: (\(\small\verb+J2+\) for \(J=2\) or \(\small\verb+J4+\) for \(J=4\))
- Name of method:
  - \(\small\verb+hatfTNP+\) for the
    proposed nonparametric estimator
  - \(\small\verb+hatfTM+\) or the
    maximum likelihood estimator
  - \(\small\verb+hatfTSP+\) for the
    semiparametric estimator with an incorrectly specified model for \(H\)
  - \(\small\verb+hatfTSPC+\) for the
    semiparametric estimator with a correctly specified model for \(H\).
- sample size: \(\small\verb+n250+\),
  \(\small\verb+n500+\) or \(\small\verb+n1000+\)
- percentage of error variance:
  - \(\small\verb+nsr10+\) when \(\textrm{NSR}=\textrm{var}(U)/\textrm{var}(X)=10\%\)
  - \(\small\verb+nsr20+\) when \(\textrm{NSR}=\textrm{var}(U)/\textrm{var}(X)=20\%\)

## 3. Data analysis from Section 7

The folder \(\verb+RealData+\)
contains the files for reproducing our real data analysis.

The file \(\small\verb+EATS\_Data.txt+\) contains the
\(W\_{ij}\)’s for the EATS data analysed
in our paper, and described in the file \(\verb+ETASDataDictionary.pdf+\). The file
\(\small\verb+EATSExample.m+\) applies
our procedure to those data, in the exact same way as described in
section 1 above, except that some of the inputs are already defined in
the Matlab file. Specifically, the only inputs you need to provide in
that file are:

- `namedirFct`: Path to the folder where you have unzipped
  the folder \(\small\verb+Functions+\).
  Example: `"C:\Users\yourname\Functions\"`
- `RscriptPath`: Full path to the Rscript executable on
  your computer. Example:
  `"C:\Program Files\R\R-4.4.0\bin\Rscript"`

The outputs are as described in section 1 above. The code also prints
these outputs to txt files, which can be used by the provided R code
\(\verb+plotsEATS.R+\) to reproduce the
figure of the paper.

## Note

The codes for the nonparametric and semiparametric estimators are for
the case where the errors have an unknown distribution. If the error
distribution is known you only need to replace \(\phi\_U\) and \(f\_U\) everywhere they appear in the code by
their known formula. It must satisfy the conditions of the paper, of
which equation (2.2).
